# Neglected tropical diseases: an effective global response to local poverty-related disease priorities

**DOI:** 10.1186/s40249-020-0630-9

**Published:** 2020-01-28

**Authors:** Dirk Engels, Xiao-Nong Zhou

**Affiliations:** 1 Uniting to Combat NTDs Support Centre, Geneva, Switzerland; 2 National Institute of Parasitic Diseases at Chinese Center for Disease Control and Prevention, Chinese Center for Tropical Diseases Research, Shanghai, 200025 People’s Republic of China; 3 World Health Organization Collaborative Centre for Tropical Diseases, Key Laboratory of Parasite and Vector Biology, Ministry of Health of China, Shanghai, 200025 People’s Republic of China; 4 School of Global Health, Chinese Center for Tropical Diseases Research, Jiatong University School of Medicine, Shanghai, 200025 People’s Republic of China

**Keywords:** Neglected tropical diseases, Diseases of poverty, Global health priorities, Integrated control

## Abstract

**Background:**

Neglected tropical diseases (NTDs) have long been overlooked in the global health agenda. They are intimately related to poverty, cause important local burdens of disease, but individually do not represent global priorities. Yet, NTDs were estimated to affect close to 2 billion people at the turn of the millennium, with a collective burden equivalent to HIV/AIDS, tuberculosis, or malaria. A global response was therefore warranted.

**Main text:**

The World Health Organization (WHO) conceived an innovative strategy in the early 2000s to combat NTDs as a group of diseases, based on a combination of five public health interventions. Access to essential NTD medicines has hugely improved thanks to strong public-private partnership involving the pharmaceutical sector. The combination of a WHO NTD roadmap with clear targets to be achieved by 2020 and game-changing partner commitments endorsed in the *London Declaration on Neglected Tropical Diseases*, have led to unprecedented progress in the implementation of large-scale preventive treatment, case management and care of NTDs. The coming decade will see as challenges the mainstreaming of these NTD interventions into Universal Health Coverage and the coordination with other sectors to get to the roots of poverty and scale up transmission-breaking interventions. Chinese expertise with the elimination of multiple NTDs, together with poverty reduction and intersectoral action piloted by municipalities and local governments, can serve as a model for the latter. The international community will also need to keep a specific focus on NTDs in order to further steer this global response, manage the scaling up and sustainment of NTD interventions globally, and develop novel products and implementation strategies for NTDs that are still lagging behind.

**Conclusions:**

The year 2020 will be crucial for the future of the global response to NTDs. Progress against the 2020 roadmap targets will be assessed, a new 2021–2030 NTD roadmap will be launched, and the London Declaration commitments will need to be renewed. It is hoped that during the coming decade the global response will be able to further build on today’s successes, align with the new global health and development frameworks, but also keep focused attention on NTDs and mobilize enough resources to see the effort effectively through to 2030.

## Background

Neglected tropical diseases (NTDs), are a group of diseases that occur under tropical and sub-tropical climate conditions and are intimately linked to poverty [1]. They therefore thrive in areas where access to adequate sanitation, clean water and healthcare is limited, and people live in proximity with animals and infective disease vectors, such as in remote and rural areas, informal settlements or conflict zones. NTDs affect some of the world’s poorest and most marginalized communities, predominantly in Africa, Asia and the Americas [1, 2].

In the early 2000s, the World Health Organization (WHO) had 17 NTDs in its portfolio [2], a diverse group of communicable diseases caused by bacteria, helminths, protozoa or viruses, such as Buruli ulcer, Chagas disease, dengue, dracunculiasis (guinea-worm disease), echinococcosis, foodborne trematodiasis, human African trypanosomiasis (sleeping sickness), leishmaniasis, leprosy, lymphatic filariasis (elephantiasis), onchocerciasis (river blindness), rabies, schistosomiasis (snail fever), soil-transmitted helminthiasis (intestinal worms), taeniasis/cysticercosis (pork tapeworm), blinding trachoma, and yaws [1, 2]. Since 2016, this list was expanded with three groups of diseases to currently include 20 NTDs or groups of NTDs. Those new NTDs include mycetoma, chromoblastomycosis and other deep mycoses; scabies and other ectoparasites; and snakebite envenoming [2].

NTDs do cause important local burdens of disease, but individually none of them represents a global priority in terms of numbers of people affected or disability adjusted life years (DALYs) lost [3]. Moreover, global attention tends to focus on killer diseases, although NTDs disable and disfigure more than they kill. DALYs due to NTDs are constituted for 56% by years lost due to disability (YLD) and for 44% by years of life lost (YLL), as compared to 7% of YLD and 93% of YLL for malaria, for example [4]. As a result, NTDs have remained largely neglected in the global health agenda.

Counted together, however, NTDs were estimated to affect close to 2 billion people at the turn of the millennium, with a collective DALY burden that was equivalent to HIV/AIDS, tuberculosis, or malaria [5]. Research into the more subtle and indirect consequences of NTDs has further revealed that beyond condemning affected people to live long years with disability and stigma, they keep children out of school, adults out of work, burden households with considerable costs to seek health care, trap communities in endless cycles of poverty and cost developing economies billions of dollars every year [4, 6].

For all the above reasons, a global response to NTDs was warranted. The challenge however lay in how to develop a common approach to the 17 very different and complex diseases. This paper gives an account of how the global response to neglected tropical diseases was built, where it stands today, and how it should evolve in the coming decade.

## Main text

### Search strategy

An electronic literature search was performed from 2012 to 2019 throughout the electronic databases of PubMed/MEDLINE. We determined our search strategy with reference to NTD control at the global level and country level, with the following five components: (i) policy with strategy formulation; (ii) innovative research; (iii) programme management; (iv) partner engagement; and (v) evaluation or assessment of the programmes. To maintain the search comprehensiveness, the search was restricted to articles with the following terms in their titles, abstracts and keywords: “NTDs”, or “control”, and “policy” or “strategy”, “innovation “or “research”, “programme” or “management”, “engagement” or “partner”, “evaluation” or “assessment”. We conducted an additional search on paper publications and grey literature (conference proceedings, abstracts, masters and doctoral theses) available in the offices of Uniting to Combat NTDs and the Department of Neglected Tropical Diseases, WHO. No restrictions were made on population and languages for the literature search.

A total of 948 articles were found through the PubMed/Medline from 2012 to 2019, with peak publication years occurring after 2016, while the highest number was in 2017.

Search items most mentioned in the publication titles were programme management and research innovation, followed by evaluation or assessment, strategy or policy, and partner engagement. A similar pattern was observed when searched for items most mentioned in the other search fields, with research innovation and programme management most mentioned, followed by strategy or policy, evaluation or assessment, and partner engagement.

The detailed search results are attached in Additional files 1 and 2.

### An innovative WHO strategy and unprecedented partner support

**Fig. 1:**
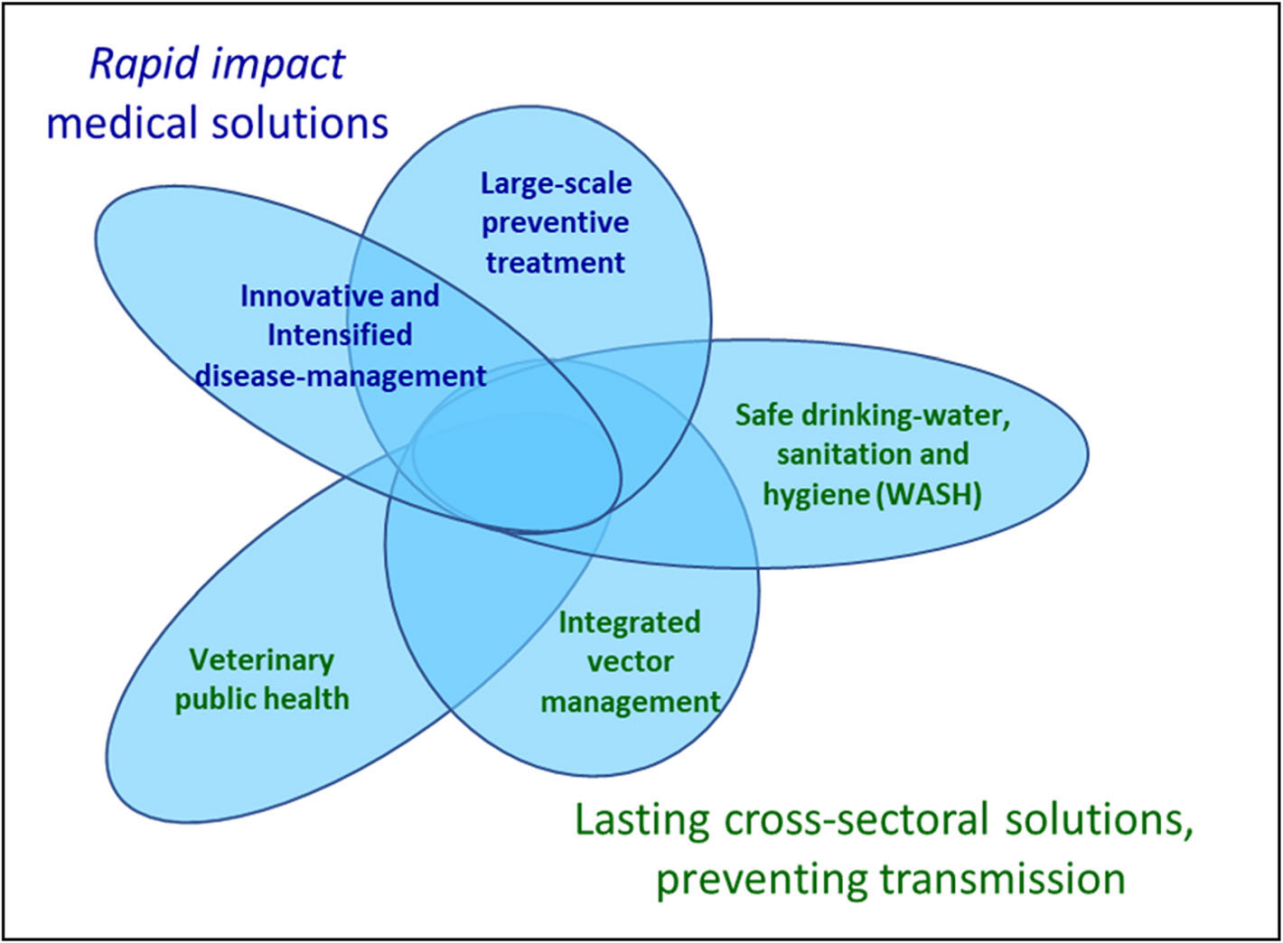
The five public health interventions recommended by WHO to overcome the impact of NTDs. NTDs: Neglected tropical diseases

By 2005, WHO had conceived an innovative strategy to combat NTDs as a group of diseases, based on a combination of five public health interventions: (i) innovative and intensified disease management; (ii) large scale preventive treatment; (iii) integrated vector management; (iv) veterinary public health; and (v) access to water, sanitation and hygiene [7] (Fig. 1). The former two are medical interventions, aimed at curing, easing or preventing acute or chronic disease, while the latter three are cross-sectoral actions aimed at impacting on the root causes of NTDs, i.e. poor living conditions and proximity with animals and vectors. In principle, NTDs can be prevented, controlled, and even eliminated by an appropriate combination of these interventions, as long as appropriate tools and commodities are available. Consequently, WHO’s rationale for including NTDs in its portfolio has been determined by its capacity to (i) make a disease immediately amenable to broad control or elimination by applying one or more of the five interventions; and/or (ii) steer innovation and find solutions in the short-to-medium term to make a disease amenable to broad control. Each of these interventions can be tailored to be effective against a range of NTDs. Multi-disease interventions are more appealing and economical for endemic countries to implement and for other sectors to be engaged in, than several disease-specific programmes being implemented in parallel.

In the early stages of its advocacy, WHO has logically focused on the need to scale up the medical interventions. These were projected to have a rapid impact on the global burden caused by NTDs, especially large-scale preventive treatment with safe, off-patent medicine that could be given in a single dose, without prior diagnosis. For intensified disease management instead, it was anticipated that large scale action would depend on considerable innovative tool development – safer, oral medicines and better diagnostics [8].

Access to essential NTD medicines has been boosted by unprecedented support from the pharmaceutical sector, in the form of large-scale donations of medicines.

The pharmaceutical company that has initiated large scale medicine donations in 1987 was US-based Merck & Co., Inc. that offered ivermectin through the *Mectizan*^*R*^ *Donation Program* for the large-scale treatment of onchocerciasis to prevent river blindness. The GSK albendazole donation for the preventive treatment of lymphatic filariasis in 1997 was the first donation through WHO. Since then, the number and volumes of medicine donations for NTDs have steadily increased [9] to reach a staggering 2.7 billion tablets – good for over 1.7 billion treatments – that were shipped to endemic countries in 2018 alone. Over the years, the donating companies have also integrated their logistics of delivery, for greater efficiency and coordination [10]. Furthermore, they have contributed to the search for improved medicines and medicine combinations, either by opening up their potential product libraries or by actively supporting product development. As a result, public-private Product Development Partnerships (PDPs) such as the Drugs for Neglected Diseases initiative, the Foundation for Innovative New Diagnostics, World Intellectual Property Organization (WIPO) Re: Search, or the Global Health Innovative Technology Fund, have literally boomed over the last 15 years and contributed to substantial progress in providing control tools for previously “non-tool-ready” NTDs [11].

By 2011, despite the innovative strategy and the medicine donations, progress in implementation had remained rather slow [12]. Hence, on 30th January 2012, WHO released an NTD roadmap with bold targets to boost prevention, control, elimination and eradication of NTDs towards 2020 [13]. The principle of the NTD roadmap was to urge wide-scale implementation of the five broad public health interventions to reach very specific targets for each of the NTDs.

**Fig. 2:**
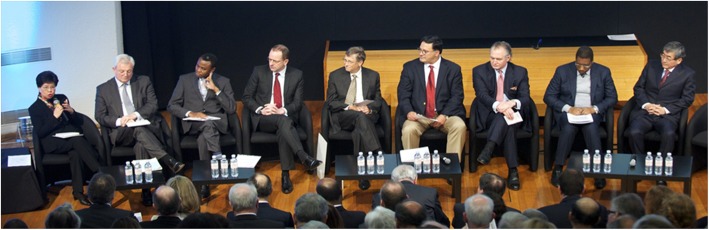
Announcement of the London Declaration on NTDs in January 2012 by representatives of key partner groups in the global effort to combat NTDs, the World Health Organization, bilateral donor agencies (UKAID and USAID), ministries of health of endemic countries (Mozambique and Tanzania), pharmaceutical companies (GSK, Merck KGaA, and Esai), and philanthropic foundations (Bill and Melinda Gates Foundation). NTDs: Neglected tropical diseases

That same day, a community of partners – today collectively known as *Uniting to Combat NTDs* – met in London to support WHO’s initiative and endorse a declaration in support of the NTD roadmap, the *London Declaration on Neglected Tropical Diseases* [14]. This declaration brought together international global health actors, development organisations, endemic countries, pharmaceutical companies and a variety of partners, who all committed to work together to control, eliminate or eradicate 10 of the NTDs in the roadmap. These 10 diseases were targeted for immediate operational support because they had the necessary ingredients to meet their roadmap goals. During the event, pharmaceutical companies have pledged donated medicines for a value of USD17.8 billion for the 10 London Declaration Diseases through to 2020, philanthropic foundations (Bill and Melinda Gates Foundation) and bilateral donors (UKAID and USAID) have pledged support to deliver those medicines to people in need, and endemic governments have committed to the deployment of their health services to make this happen (Fig. 2).

In May 2013, leveraging WHO’s affiliation with its Member States, the World Health Assembly (WHA) has adopted a resolution on all 17 neglected tropical diseases (WHA66.12), encouraging endemic countries to ensure country ownership and predictable long-term financing to integrate control programmes into primary health care and achieve universal access to NTD interventions [15].

### Reaching over 1 billion people every year

The combination of the WHO NTD roadmap and the London Declaration, together with enhanced commitment by endemic countries has been game-changing for the scaling up of NTD interventions. In 2012, 700 million people were reached with NTD treatments, representing less than 37% of the 1.9 billion at risk. By 2015, the number of people reached had increased to 980 million, with the one billion effectively being crossed as of 2016 onwards, for three years in a row. In 2018, 1.12 billion people were reached representing close to 65% of those at risk [16], at which yearly calculation of populations and coverages have taken into account the ongoing population expansion in age groups eligible for treatment.

After several years of high coverage with preventive treatment, NTDs such as lymphatic filariasis, blinding trachoma and onchocerciasis are being eliminated. This has led to already 400 million people being freed of those diseases and not requiring preventive treatments anymore. As a result, 31 countries have eliminated at least one NTD since 2012, as compared to only 13 during the entire pre-London Declaration period [17].

Considerable progress has also been made with mapping of NTDs, which brought up the number of people at risk from 1.5 billion in 2016 to 1.75 billion in 2018 [16]. Mapping efforts included the largest infectious disease survey in history – the global trachoma mapping project. It collected data from 2.6 million people across 29 countries and, in turn, served as a model for region-wide mapping of the other four most prevalent NTDs (lymphatic filariasis, onchocerciasis, soil-transmitted helminthiasis, schistosomiasis and trachoma) in Africa by the Expanded Special Project for the Elimination of Neglected Tropical Diseases (ESPEN) [18]. ESPEN was established in 2016, following the closure of the African Programme for Onchocerciasis Control, in the spirit of public-private partnership between the WHO Regional Office for Africa, endemic countries and NTD partners in an effort to mobilize political, technical and financial resources to accelerate the elimination of Neglected Tropical Diseases in sub-Saharan Africa.

Expanded disease mapping, systematic data collection and monitoring of progress has allowed to know where the worlds neediest are, to track equitable access to preventive treatments, and to hold countries accountable [19, 20]. As an example, a recent collaborative initiative between *Uniting to Combat NTDs,* the WHO including ESPEN, and the Organization of African Unity (OAU), like the Africa Leaders’ Malaria Alliance, has introduced a combined coverage score for the five most prevalent NTDs. This aggregate coverage figure that reflects a country’s performance in implementing large scale preventive treatment is reviewed every year by African Heads of State at the OAU General Assembly. In the three years for which the NTD score has been calculated, 2015, 2016 and 2017, great progress has been achieved, with 15 OAU member countries having achieved the WHO recommended coverage of 75% or more for all five NTDs in 2017, as compared to only 3 in 2015. The number of countries having achieved between 25 and 74% coverage increased from 11 to 14 over the period 2015–2017, while the number of countries with coverage below 25% decreased from 34 to 20 [21].

The innovative and intensified disease management (IDM) diseases, such as African sleeping sickness, Buruli ulcer, Chagas disease, leprosy and leishmaniasis, are NTDs for which cost-effective control tools did not exist and large-scale use of existing tools could not be envisaged. These diseases need to be managed within the primary health-care system [22, 23]. Yet, the recent history of strong public-private partnership driven by novel thinking has proven that these complex diseases can be lifted out of neglect, and ultimately even be eliminated as public health problems [24]. A prime example of the former is Buruli ulcer, for which a good decade ago management consisted solely of aggressive surgery and for which now an early diagnostic and oral treatment are available [25, 26].

The latter is illustrated by the unprecedented progress made with sleeping sickness elimination. Because of a breakdown of systematic control programmes, the disease had resurged from below 5000 reported cases per year in the early 1960s to close to 40 000 cases per year, mostly late-stage disease, by the late 1990s. Partnering between WHO, the pharmaceutical sector, endemic countries, nongovernmental organizations and newly established PDPs has led to transformative action in fast-tracking the development of novel treatments such as NECT (nifurtimox-eflornithine combination treatment, merging two already existing drugs), and later oral fexinidazole [27]. Complex but well-organized logistics have ensured distribution of medicines down to peripheral health care facilities. As a result, the number of reported cases has decreased to 977 cases in 2018, a more than 95% case reduction since 2000. This was accompanied by a substantial decrease in the number of people at high or moderate risk of contracting sleeping sickness in previously highly endemic areas. The 2018 figures are already well below what was targeted by the WHO NTD roadmap in terms of “elimination a public health problem” by the year 2020 [28]. The ongoing development of oral acoziborole that potentially could be given as a single-dose treatment opens perspectives to interrupt transmission of sleeping sickness in the coming decade [29].

Similar principles have shown their power also in sub-regional initiatives such as the elimination of visceral leishmaniasis in South-East Asia. Three high-burden countries — Bangladesh, India and Nepal — have teamed up to eliminate this deadly disease as a public health problem by 2020 [30]. This initiative has been built on robust involvement of national health services and strong international collaboration [31], having taken to scale a combination of early diagnosis and systematic treatment with donated liposomal amphotericin B (Ambisome^R^) and vector control. Even though some challenges remain, the perspective for elimination has become real [32].

### From MDGs to SDGs: new challenges and opportunities for NTDs

Even though NTDs were not specifically mentioned in the Millennium Development Goals (MDGs), the successful scaling up of ‘medical’ NTD interventions was very much in line with progress achieved for HIV/AIDS, tuberculosis and malaria. Sustained implementation and pivotal combination with interventions impacting transmission, as already strongly recommended by WHO in 2005 [8], have highlighted the potential for large scale elimination of NTDs. The international commitment, in 2015, towards a broader development agenda (the Sustainable Development Goals, SDGs) with a strong focus on equity (leaving no one behind), provided new opportunities for NTDs to promote cross-sectoral action and thus fundamentally prevent transmission [33]. The road towards 2030 therefore presents two major challenges for NTDs: (i) mainstreaming the ‘medical’ NTD interventions into primary health care and making sure they are part of the Universal Health Coverage (UHC) package in poverty-stricken areas; and (ii) coordinating with other sectors to scale up transmission-breaking interventions. NTDs are diseases of poverty and efforts to eliminate them need to get to the roots of that poverty [34]. NTDs can therefore be used as an indicator to attract development to where it is most needed and will have most impact. This perspective was strongly voiced during The Second Global Partners Meeting on NTDs in WHO and WHO’s Fourth Report on Neglected Tropical Diseases [35, 36].

**Fig. 3:**
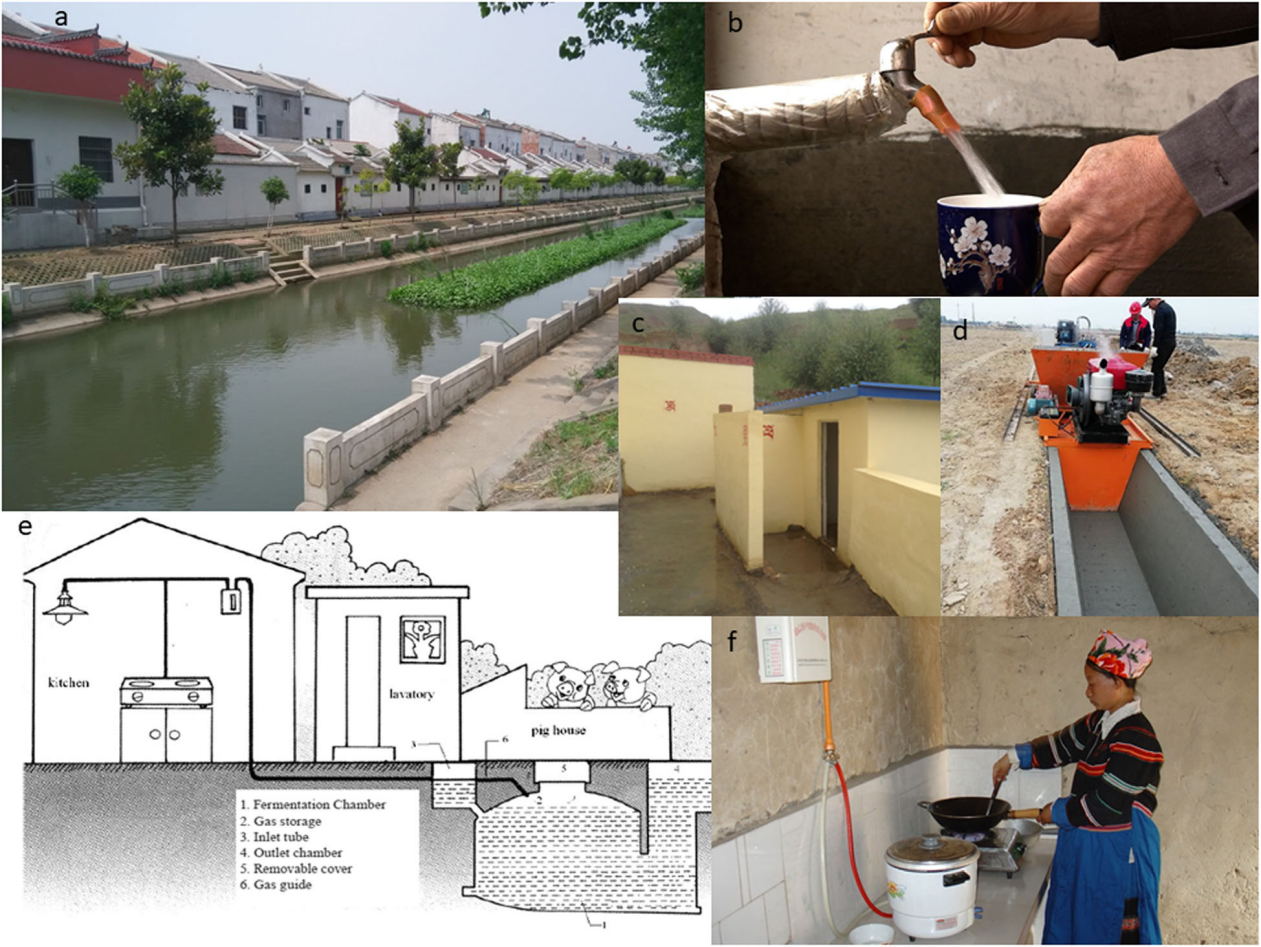
Environmental measures to limit transmission sources of schistosomiasis and soil-transmitted helminthiasis in China (**a** Environmental modification performed by cementing along both sides of a canal where was the habitat of *Oncomelania* snails; **b** Safe water provided to each household by subsidy programmes from local government; **c** This new village latrine was built in a hyper-endemic village of schistosomiasis; **d** New irrigation system being built in farmland that was the habitat of *Oncomelania* snails; **e** The diagram of bio-gas chambers for faecal fermentation to eliminate eggs of schistosomes; **f** A housewife is cooking a meal in her kitchen room with the energy supplied by bio-gas transferred by a tube connected to the faecal fermentation chambers)

Countries that have made major strides against poverty during the MDG era, such as China, have used this approach since the early 2000s. Best documented for the elimination of schistosomiasis, a disease that has had high political priority since the founding of the People’s Republic of China in 1949, such integrated, multidisciplinary approach to disease control combined environmental improvements with industrial, agricultural and water resource development projects to reduce disease transmission while boosting local economic development (Fig. 3) [37–39]. Key elements of success have been: (i) strong national policies in favour of multidisciplinary disease control; (ii) intersectoral action for health and development, piloted by municipalities and local governments; and (iii) a strong emphasis on equity and poverty reduction in areas where it was most needed [40]. Lessons can and should be learned from the Chinese experience in eliminating NTDs [40, 41], including its concern for continuously updating strategies and interventions, and maintaining surveillance-response systems at all levels, if one wishes to achieve sustainable elimination of NTDs in the next decade [42, 43].

### 2020: a pivotal year

The year 2020 will be crucial for the future of the global response to NTDs. Progress against the 2020 NTD roadmap targets will be assessed, a new 2021–2030 NTD roadmap will be launched by WHO — now for the 20 NTDs in its portfolio, and the London Declaration commitments will need to be renewed.

While the new NTD roadmap will need to build further on the potential for elimination with current interventions and an anticipated 90% fewer people requiring NTD interventions by 2030, as specified in the SDGs indicator of NTDs, it will also need to steer the integration of NTD interventions into national systems. NTD interventions will only become an integral part of UHC when it is recognized that they are local priorities of poverty and that the health system needs to be responsive to them. This requires sound technical and managerial knowledge of NTDs at all levels of the health system, that currently still largely needs to be built.

Another challenge is for the health system, under strong national directives, to link up with municipalities and local governments to solicit cross-sectoral action — for basic water supply, adequate sanitary infrastructure and improved housing, for an integrated vector control response, for the coordination of interventions with agriculture and livestock management — to sustain achievements and alleviate the underlying socio-economic determinants of NTDs. All this will require adequate resources and innovative financing mechanisms that will need to be thought of, such as leveraging broader investments in development with small incremental NTD-specific components.

While it is expected that domestic funding for NTDs will increase and approach the levels required to cope with the residual burden of NTDs towards 2030, some earmarked funding for NTDs will remain crucial over the coming decade to further scale up implementation, to build widespread NTD capacity, and to take care of required innovation and product development. And the needs for the latter can’t be underestimated. While there is a recognized need for novel diagnostics across the NTD spectrum [44], there is also a need to further improve large scale access to medicines or commodities — for human or animal use — for diseases such as echinococcosis, foodborne trematodiasis, rabies, scabies and taeniasis/cysticercosis. Some NTDs such as cutaneous leishmaniasis, mycetoma and deep mycoses still have no first line, oral medicines hampering widespread access to early treatment and care. Snakebite envenoming would require a vigorous transformational investment in the development of heat-stable, polyvalent antivenoms to make them fully accessible in resource-poor peripheral settings [45].

In the current global health environment, with NTDs not having a “focused” funding mechanism such as GAVI (The Global Alliance for Vaccines and Immunisation) or the Global Fund Against HIV/AIDS, Tuberculosis and Malaria, and with international donors tending to move away from earmarked funding, the risk is that local priorities of poverty such as NTDs may slide back into neglect, as has been the case during the “Health for All by the year 2000” era.

## Conclusions

The global response to neglected tropical diseases has been mounted to expressly help the poor and pull their poverty-related health problems out of neglect. Driven by novel thinking and boosted by strong public-private partnership the effort has taken proportions that are unmatched in scope today.

Yet there is no room for complacency. All neglect has not been resolved, and despite unprecedented progress the job is not finished. It is hoped that during the coming decade the global response will further be able to build on successes achieved today, align with the new global health and development frameworks while keeping very focused attention on the development of novel products, the filling of knowledge gaps associated with implementation, and the generation of evidence-based science from field and laboratory for NTDs that are still lagging behind. It is also hoped that at the renewal of the London Declaration commitment, enough resources can be mobilized to see this global response in all its aspects through to its projected end in 2030.

## Supplementary information

**Additional file 1.** List of the articles: results from the electronic literature search from PubMed/Medline.

**Additional file 2.** Electronic Literature Searching Strategy and Results.

## Data Availability

Not applicable.

## Abbreviations

DALYs: Disability adjusted life years
ESPEN: Expanded Special Project for the Elimination of Neglected Tropical Diseases
GAVI: The Global Alliance for Vaccines and Immunisation
HIV/AIDS: Human immunodeficiency virus/acquired immunodeficiency syndrome
IDM: Innovative and intensified disease management
MDGs: Millennium Development Goals
NECT: Nifurtimox-Eflornithine Combination Treatment
NTDs: Neglected tropical diseases
OAU: Organization of African Unity
SDGs: Sustainable Development Goals
UHC: Universal Health Coverage
WHA: World Health Assembly
WHO: World Health Organization
WIPO: World Intellectual Property Organization
YLD: Years Lost due to Disability
YLL: Years of life lost
