# Supplementary material for: Neglected tropical diseases: an effective global response to local poverty-related disease priorities
Source: Infect Dis Poverty. 2020 Jan 28;9:10. doi: 10.1186/s40249-020-0630-9 (PMC6986060; doi:10.1186/s40249-020-0630-9)
Supplement: Supplementary file 1 — Additional file 1. List of the articles: results from the electronic literature search from PubMed/Medline. [file 40249_2020_630_MOESM1_ESM.docx]

***S1***

**List of the articles: results from the electronic literature search from PubMed/Medline**

A total of 948 articles were found through the PubMed/Medline from 2012 to 2019 as follows:

1. Zuffo M, Stucchi A, Campos-Salinas J, Cabello-Donayre M, Martinez-Garcia M, Belmonte-Reche E, et al. Carbohydrate-naphthalene diimide conjugates as potential antiparasitic drugs: Synthesis, evaluation and structure-activity studies. Eur J Med Chem. 2019;163:54-66; doi: 10.1016/j.ejmech.2018.11.043. https://www.ncbi.nlm.nih.gov/pubmed/30503943.

2. Zhou XN, Leonardo L, Bergquist R. Preface: Sustained cooperation on research and control of neglected tropical diseases among multisectors and multipartners across borders in Southeast Asia. Adv Parasitol. 2019;105:xi-xiii; doi: 10.1016/S0065-308X(19)30059-4. https://www.ncbi.nlm.nih.gov/pubmed/31530398.

3. Zhao Y, Jiang M, Wu Y, Song F, Cai W, Li H. Mitochondrial genomes of three kissing bugs (Reduviidae: Triatominae) and their phylogenetic implications. Int J Biol Macromol. 2019;134:36-42; doi: 10.1016/j.ijbiomac.2019.05.020. https://www.ncbi.nlm.nih.gov/pubmed/31071392.

4. Zhang H, Zhang C, Zhu Y, Mehmood K, Liu J, McDonough SP, et al. Leptospirosis trends in China, 2007-2018: A retrospective observational study. Transbound Emerg Dis. 2019; doi: 10.1111/tbed.13437. https://www.ncbi.nlm.nih.gov/pubmed/31765064.

5. Zhang C, Xu J, Zhang T, Qiu H, Li Z, Zhang E, et al. Genetic characteristics of pathogenic Leptospira in wild small animals and livestock in Jiangxi Province, China, 2002-2015. PLoS Negl Trop Dis. 2019;13 6:e0007513; doi: 10.1371/journal.pntd.0007513. https://www.ncbi.nlm.nih.gov/pubmed/31233503.

6. Zammarchi L, Gobbi F, Angheben A, Spinicci M, Buonfrate D, Calleri G, et al. Schistosomiasis, strongyloidiasis and Chagas disease: the leading imported Neglected Tropical Diseases in Italy. J Travel Med. 2019; doi: 10.1093/jtm/taz100. https://www.ncbi.nlm.nih.gov/pubmed/31840757.

7. Zahedifard F, Lee H, No JH, Salimi M, Seyed N, Asoodeh A, et al. Comparative study of different forms of Jellein antimicrobial peptide on Leishmania parasite. Exp Parasitol. 2019;209:107823; doi: 10.1016/j.exppara.2019.107823. https://www.ncbi.nlm.nih.gov/pubmed/31862270.

8. Youssefi MR, Moghaddas E, Tabari MA, Moghadamnia AA, Hosseini SM, Farash BRH, et al. In Vitro and In Vivo Effectiveness of Carvacrol, Thymol and Linalool against Leishmania infantum. Molecules. 2019;24 11; doi: 10.3390/molecules24112072. https://www.ncbi.nlm.nih.gov/pubmed/31151304.

9. Wrable M, Kulinkina AV, Liss A, Koch M, Cruz MS, Biritwum NK, et al. The use of remotely sensed environmental parameters for spatial and temporal schistosomiasis prediction across climate zones in Ghana. Environ Monit Assess. 2019;191 Suppl 2:301; doi: 10.1007/s10661-019-7411-6. https://www.ncbi.nlm.nih.gov/pubmed/31254149.

10. Williams DJ, Faiz MA, Abela-Ridder B, Ainsworth S, Bulfone TC, Nickerson AD, et al. Strategy for a globally coordinated response to a priority neglected tropical disease: Snakebite envenoming. PLoS Negl Trop Dis. 2019;13 2:e0007059; doi: 10.1371/journal.pntd.0007059. https://www.ncbi.nlm.nih.gov/pubmed/30789906.

11. Wainwright E, Evans D, Rotondo L, Pou B, Yevstigneyeva V, Zoerhoff KL, et al. The Elimination of Neglected Tropical Diseases (NTDs): A Case Study Exemplifying How Foreign Assistance Funding Can Be Catalytic in Reducing the Burden of Major Global Health Conditions. Clin Infect Dis. 2019; doi: 10.1093/cid/ciz742. https://www.ncbi.nlm.nih.gov/pubmed/31402376.

12. Versteeg L, Almutairi MM, Hotez PJ, Pollet J. Enlisting the mRNA Vaccine Platform to Combat Parasitic Infections. Vaccines (Basel). 2019;7 4; doi: 10.3390/vaccines7040122. https://www.ncbi.nlm.nih.gov/pubmed/31547081.

13. Tamiru HF, Mashalla YJ, Mohammed R, Tshweneagae GT. Cutaneous leishmaniasis a neglected tropical disease: community knowledge, attitude and practices in an endemic area, Northwest Ethiopia. BMC Infect Dis. 2019;19 1:855; doi: 10.1186/s12879-019-4506-1. https://www.ncbi.nlm.nih.gov/pubmed/31619180.

14. Sutherland CS, Tediosi F. Is the elimination of 'sleeping sickness' affordable? Who will pay the price? Assessing the financial burden for the elimination of human African trypanosomiasis Trypanosoma brucei gambiense in sub-Saharan Africa. BMJ Glob Health. 2019;4 2:e001173; doi: 10.1136/bmjgh-2018-001173. https://www.ncbi.nlm.nih.gov/pubmed/31139437.

15. Sunyoto T, Potet J, den Boer M, Ritmeijer K, Postigo JAR, Ravinetto R, et al. Exploring global and country-level barriers to an effective supply of leishmaniasis medicines and diagnostics in eastern Africa: a qualitative study. BMJ Open. 2019;9 5:e029141; doi: 10.1136/bmjopen-2019-029141. https://www.ncbi.nlm.nih.gov/pubmed/31152044.

16. Staniek ME, Sedda L, Gibson TD, de Souza CF, Costa EM, Dillon RJ, et al. eNose analysis of volatile chemicals from dogs naturally infected with Leishmania infantum in Brazil. PLoS Negl Trop Dis. 2019;13 8:e0007599; doi: 10.1371/journal.pntd.0007599. https://www.ncbi.nlm.nih.gov/pubmed/31386662.

17. Sotillo J, Pearson MS, Loukas A. Trematode Genomics and Proteomics. Adv Exp Med Biol. 2019;1154:411-36; doi: 10.1007/978-3-030-18616-6_13. https://www.ncbi.nlm.nih.gov/pubmed/31297769.

18. Smith ME, Bilal S, Lakwo TL, Habomugisha P, Tukahebwa E, Byamukama E, et al. Accelerating river blindness elimination by supplementing MDA with a vegetation "slash and clear" vector control strategy: a data-driven modeling analysis. Sci Rep. 2019;9 1:15274; doi: 10.1038/s41598-019-51835-0. https://www.ncbi.nlm.nih.gov/pubmed/31649285.

19. Siyadatpanah A, Anvari D, Emami Zeydi A, Hosseini SA, Daryani A, Sarvi S, et al. A systematic review and meta-analysis of the genetic characterization of human echinococcosis in Iran, an endemic country. Epidemiol Health. 2019;41:e2019024; doi: 10.4178/epih.e2019024. https://www.ncbi.nlm.nih.gov/pubmed/31208193.

20. Shymanovich T, Faw L, Hajhashemi N, Teague J, Schal C, Ponnusamy L, et al. Diel periodicity and visual cues guide oviposition behavior in Phlebotomus papatasi, vector of old-world cutaneous leishmaniasis. PLoS Negl Trop Dis. 2019;13 3:e0007165; doi: 10.1371/journal.pntd.0007165. https://www.ncbi.nlm.nih.gov/pubmed/30835733.

21. Shumbej T, Girum T. Helminth infections in light of an ongoing intervention in endemic areas of Guragae zone, Southern Ethiopia: an implication for neglected tropical diseases elimination in Ethiopia by 2020. Trop Dis Travel Med Vaccines. 2019;5:8; doi: 10.1186/s40794-019-0083-y. https://www.ncbi.nlm.nih.gov/pubmed/31073412.

22. Shoemaker EA, Dale K, Cohn DA, Kelly MP, Zoerhoff KL, Batcho WE, et al. Gender and neglected tropical disease front-line workers: Data from 16 countries. PLoS One. 2019;14 12:e0224925; doi: 10.1371/journal.pone.0224925. https://www.ncbi.nlm.nih.gov/pubmed/31856174.

23. Serban G. Future Prospects in the Treatment of Parasitic Diseases: 2-Amino-1,3,4-Thiadiazoles in Leishmaniasis. Molecules. 2019;24 8; doi: 10.3390/molecules24081557. https://www.ncbi.nlm.nih.gov/pubmed/31010226.

24. Sato MO, Adsakwattana P, Fontanilla IKC, Kobayashi J, Sato M, Pongvongsa T, et al. Odds, challenges and new approaches in the control of helminthiasis, an Asian study. Parasite Epidemiol Control. 2019;4:e00083; doi: 10.1016/j.parepi.2018.e00083. https://www.ncbi.nlm.nih.gov/pubmed/30662968.

25. Satjawongvanit H, Phumee A, Tiawsirisup S, Sungpradit S, Brownell N, Siriyasatien P, et al. Molecular Analysis of Canine Filaria and Its Wolbachia Endosymbionts in Domestic Dogs Collected from Two Animal University Hospitals in Bangkok Metropolitan Region, Thailand. Pathogens. 2019;8 3; doi: 10.3390/pathogens8030114. https://www.ncbi.nlm.nih.gov/pubmed/31362350.

26. Sanmartino M, Avaria Saavedra A, Gomez IPJ, Albajar-Vinas P. Chagas and health promotion: dialogue inspired by the Curitiba Statement. Health Promot Int. 2019;34 Supplement_1:i82-i91; doi: 10.1093/heapro/day105. https://www.ncbi.nlm.nih.gov/pubmed/30690456.

27. Sanchez-Hernandez D, Aguirre-Salado CA, Sanchez-Diaz G, Aguirre-Salado AI, Soubervielle-Montalvo C, Reyes-Cardenas O, et al. Modeling spatial pattern of dengue in North Central Mexico using survey data and logistic regression. Int J Environ Health Res. 2019:1-17; doi: 10.1080/09603123.2019.1700938. https://www.ncbi.nlm.nih.gov/pubmed/31835907.

28. Salm A, Gertsch J. Cultural perception of triatomine bugs and Chagas disease in Bolivia: a cross-sectional field study. Parasit Vectors. 2019;12 1:291; doi: 10.1186/s13071-019-3546-0. https://www.ncbi.nlm.nih.gov/pubmed/31182163.

29. Sacolo-Gwebu H, Chimbari M, Kalinda C. Prevalence and risk factors of schistosomiasis and soil-transmitted helminthiases among preschool aged children (1-5 years) in rural KwaZulu-Natal, South Africa: a cross-sectional study. Infect Dis Poverty. 2019;8 1:47; doi: 10.1186/s40249-019-0561-5. https://www.ncbi.nlm.nih.gov/pubmed/31202273.

30. Rufai T, Aninagyei E, Sackey SO, Kenu E, Afari EA. Evaluation of Buruli Ulcer Disease Surveillance System in the Ga West Municipality, Ghana, 2011-2015. J Trop Med. 2019;2019:4721236; doi: 10.1155/2019/4721236. https://www.ncbi.nlm.nih.gov/pubmed/31781253.

31. Rivadeneira DJ, Luo H. Jejunal Ulcer Caused by Schistosoma japonicum. Case Rep Gastrointest Med. 2019;2019:8356438; doi: 10.1155/2019/8356438. https://www.ncbi.nlm.nih.gov/pubmed/31049231.

32. Richards L, Erko B, Ponpetch K, Ryan SJ, Liang S. Assessing the nonhuman primate reservoir of Schistosoma mansoni in Africa: a systematic review. Infect Dis Poverty. 2019;8 1:32; doi: 10.1186/s40249-019-0543-7. https://www.ncbi.nlm.nih.gov/pubmed/31077256.

33. Reguera RM, Elmahallawy EK, Garcia-Estrada C, Carbajo-Andres R, Balana-Fouce R. DNA Topoisomerases of Leishmania Parasites; Druggable Targets for Drug Discovery. Curr Med Chem. 2019;26 32:5900-23; doi: 10.2174/0929867325666180518074959. https://www.ncbi.nlm.nih.gov/pubmed/29773051.

34. Rees CA, Hotez PJ, Monuteaux MC, Niescierenko M, Bourgeois FT. Neglected tropical diseases in children: An assessment of gaps in research prioritization. PLoS Negl Trop Dis. 2019;13 1:e0007111; doi: 10.1371/journal.pntd.0007111. https://www.ncbi.nlm.nih.gov/pubmed/30695020.

35. Qiu C, Lu DB, Deng Y, Zou HY, Liang YS, Webster JP. Population genetics of Oncomelania hupensis snails, intermediate hosts of Schistosoma japonium, from emerging, re-emerging or established habitats within China. Acta Trop. 2019;197:105048; doi: 10.1016/j.actatropica.2019.105048. https://www.ncbi.nlm.nih.gov/pubmed/31173738.

36. Qian YJ, Ding W, Wu WP, Bandikhuu A, Damdindorj T, Nyamdorj T, et al. A path to cooperation between China and Mongolia towards the control of echinococcosis under the Belt and Road Initiative. Acta Trop. 2019;195:62-7; doi: 10.1016/j.actatropica.2019.04.022. https://www.ncbi.nlm.nih.gov/pubmed/31009597.

37. Qian MB, Chen J, Bergquist R, Li ZJ, Li SZ, Xiao N, et al. Neglected tropical diseases in the People's Republic of China: progress towards elimination. Infect Dis Poverty. 2019;8 1:86; doi: 10.1186/s40249-019-0599-4. https://www.ncbi.nlm.nih.gov/pubmed/31578147.

38. Pisarski K. The Global Burden of Disease of Zoonotic Parasitic Diseases: Top 5 Contenders for Priority Consideration. Trop Med Infect Dis. 2019;4 1; doi: 10.3390/tropicalmed4010044. https://www.ncbi.nlm.nih.gov/pubmed/30832380.

39. Parker C, Garcia F, Menocal O, Jeer D, Alto B. A Mosquito Workshop and Community Intervention: A Pilot Education Campaign to Identify Risk Factors Associated with Container Mosquitoes in San Pedro Sula, Honduras. Int J Environ Res Public Health. 2019;16 13; doi: 10.3390/ijerph16132399. https://www.ncbi.nlm.nih.gov/pubmed/31284544.

40. Pandarakalam GC, Speake M, McElroy S, Alturkistani A, Philippe L, Pettitt J, et al. A high-throughput screen for the identification of compounds that inhibit nematode gene expression by targeting spliced leader trans-splicing. Int J Parasitol Drugs Drug Resist. 2019;10:28-37; doi: 10.1016/j.ijpddr.2019.04.001. https://www.ncbi.nlm.nih.gov/pubmed/31015150.

41. Palmer JJ. Sensing Sleeping Sickness: Local Symptom-Making in South Sudan. Med Anthropol. 2019:1-17; doi: 10.1080/01459740.2019.1689976. https://www.ncbi.nlm.nih.gov/pubmed/31852244.

42. Otabil KB, Gyasi SF, Awuah E, Obeng-Ofori D, Atta-Nyarko RJ, Andoh D, et al. Prevalence of onchocerciasis and associated clinical manifestations in selected hypoendemic communities in Ghana following long-term administration of ivermectin. BMC Infect Dis. 2019;19 1:431; doi: 10.1186/s12879-019-4076-2. https://www.ncbi.nlm.nih.gov/pubmed/31101085.

43. Osti MH, Sokana O, Phelan S, Marks M, Whitfeld MJ, Gorae C, et al. Prevalence of scabies and impetigo in the Solomon Islands: a school survey. BMC Infect Dis. 2019;19 1:803; doi: 10.1186/s12879-019-4382-8. https://www.ncbi.nlm.nih.gov/pubmed/31519153.

44. Onzo-Aboki A, Ibikounle M, Boko PM, Savassi BS, Doritchamou J, Siko EJ, et al. Human schistosomiasis in Benin: Countrywide evidence of Schistosoma haematobium predominance. Acta Trop. 2019;191:185-97; doi: 10.1016/j.actatropica.2019.01.004. https://www.ncbi.nlm.nih.gov/pubmed/30633895.

45. Oluwole A, Dean L, Lar L, Salami K, Okoko O, Isiyaku S, et al. Optimising the performance of frontline implementers engaged in the NTD programme in Nigeria: lessons for strengthening community health systems for universal health coverage. Hum Resour Health. 2019;17 1:79; doi: 10.1186/s12960-019-0419-8. https://www.ncbi.nlm.nih.gov/pubmed/31675965.

46. Ofon E, Noyes H, Ebo'o Eyanga V, Njiokou F, Koffi M, Fogue P, et al. Association between IL1 gene polymorphism and human African trypanosomiasis in populations of sleeping sickness foci of southern Cameroon. PLoS Negl Trop Dis. 2019;13 3:e0007283; doi: 10.1371/journal.pntd.0007283. https://www.ncbi.nlm.nih.gov/pubmed/30908482.

47. Nieto-Sanchez C, Bates BR, Guerrero D, Jimenez S, Baus EG, Peeters Grietens K, et al. Home improvement and system-based health promotion for sustainable prevention of Chagas disease: A qualitative study. PLoS Negl Trop Dis. 2019;13 6:e0007472; doi: 10.1371/journal.pntd.0007472. https://www.ncbi.nlm.nih.gov/pubmed/31194754.

48. Ngowi HA, Winkler AS, Braae UC, Mdegela RH, Mkupasi EM, Kabululu ML, et al. Taenia solium taeniosis and cysticercosis literature in Tanzania provides research evidence justification for control: A systematic scoping review. PLoS One. 2019;14 6:e0217420; doi: 10.1371/journal.pone.0217420. https://www.ncbi.nlm.nih.gov/pubmed/31166983.

49. Mwandawiro C, Okoyo C, Kihara J, Simiyu E, Kepha S, Campbell SJ, et al. Results of a national school-based deworming programme on soil-transmitted helminths infections and schistosomiasis in Kenya: 2012-2017. Parasit Vectors. 2019;12 1:76; doi: 10.1186/s13071-019-3322-1. https://www.ncbi.nlm.nih.gov/pubmed/30732642.

50. Mutombo N, Landoure A, Man WY, Fenwick A, Dembele R, Sacko M, et al. The association between child Schistosoma spp. infections and morbidity in an irrigated rice region in Mali: A localized study. Acta Trop. 2019;199:105115; doi: 10.1016/j.actatropica.2019.105115. https://www.ncbi.nlm.nih.gov/pubmed/31356787.

51. Murthy PK. Strategies to Control Human Lymphatic Filarial Infection: Tweaking Host's Immune System. Curr Top Med Chem. 2019;19 14:1226-40; doi: 10.2174/1568026619666190618110613. https://www.ncbi.nlm.nih.gov/pubmed/31244425.

52. Mubanga C, Mwape KE, Phiri IK, Trevisan C, Zulu G, Chabala C, et al. Progress on the development of rapid diagnostic tests for foodborne neglected zoonotic helminthiases: A systematic review. Acta Trop. 2019;194:135-47; doi: 10.1016/j.actatropica.2019.03.030. https://www.ncbi.nlm.nih.gov/pubmed/30946810.

53. Mollett G, Bremer Hinckel BC, Bhattacharyya T, Marlais T, Singh OP, Mertens P, et al. Detection of Immunoglobulin G1 Against rK39 Improves Monitoring of Treatment Outcomes in Visceral Leishmaniasis. Clin Infect Dis. 2019;69 7:1130-5; doi: 10.1093/cid/ciy1062. https://www.ncbi.nlm.nih.gov/pubmed/30541022.

54. Marks M, Toloka H, Baker C, Kositz C, Asugeni J, Puiahi E, et al. Randomized Trial of Community Treatment With Azithromycin and Ivermectin Mass Drug Administration for Control of Scabies and Impetigo. Clin Infect Dis. 2019;68 6:927-33; doi: 10.1093/cid/ciy574. https://www.ncbi.nlm.nih.gov/pubmed/29985978.

55. Marks M, Romani L, Sokana O, Neko L, Harrington R, Nasi T, et al. Prevalence of scabies and impetigo three years after mass drug administration with ivermectin and azithromycin. Clin Infect Dis. 2019; doi: 10.1093/cid/ciz444. https://www.ncbi.nlm.nih.gov/pubmed/31131410.

56. Marks M, McVernon J, Engelman D, Kaldor J, Steer A. Insights from mathematical modelling on the proposed WHO 2030 goals for scabies. Gates Open Res. 2019;3:1542; doi: 10.12688/gatesopenres.13064.1. https://www.ncbi.nlm.nih.gov/pubmed/31656953.

57. Malecela MN. Reflections on the decade of the neglected tropical diseases. Int Health. 2019;11 5:338-40; doi: 10.1093/inthealth/ihz048. https://www.ncbi.nlm.nih.gov/pubmed/31529110.

58. Makenga Bof JC, Ntumba Tshitoka F, Muteba D, Mansiangi P, Coppieters Y. Review of the National Program for Onchocerciasis Control in the Democratic Republic of the Congo. Trop Med Infect Dis. 2019;4 2; doi: 10.3390/tropicalmed4020092. https://www.ncbi.nlm.nih.gov/pubmed/31200509.

59. Maier T, Wheeler NJ, Namigai EKO, Tycko J, Grewelle RE, Woldeamanuel Y, et al. Gene drives for schistosomiasis transmission control. PLoS Negl Trop Dis. 2019;13 12:e0007833; doi: 10.1371/journal.pntd.0007833. https://www.ncbi.nlm.nih.gov/pubmed/31856157.

60. Mackinnon E, Ayah R, Taylor R, Owor M, Ssempebwa J, Olago LD, et al. 21st century research in urban WASH and health in sub-Saharan Africa: methods and outcomes in transition. Int J Environ Health Res. 2019;29 4:457-78; doi: 10.1080/09603123.2018.1550193. https://www.ncbi.nlm.nih.gov/pubmed/30545246.

61. Machin L, Tamargo B, Pinon A, Aties RC, Scull R, Setzer WN, et al. Bixa orellana L. (Bixaceae) and Dysphania ambrosioides (L.) Mosyakin & Clemants (Amaranthaceae) Essential Oils Formulated in Nanocochleates against Leishmania amazonensis. Molecules. 2019;24 23; doi: 10.3390/molecules24234222. https://www.ncbi.nlm.nih.gov/pubmed/31757083.

62. Machado G, Alvarez J, Bakka HC, Perez A, Donato LE, de Ferreira Lima Junior FE, et al. Revisiting area risk classification of visceral leishmaniasis in Brazil. BMC Infect Dis. 2019;19 1:2; doi: 10.1186/s12879-018-3564-0. https://www.ncbi.nlm.nih.gov/pubmed/30606104.

63. Macfarlane CL, Dean L, Thomson R, Garner P. Community drug distributors for mass drug administration in neglected tropical disease programmes: systematic review and analysis of policy documents. J Glob Health. 2019;9 2:020414; doi: 10.7189/jogh.09.020414. https://www.ncbi.nlm.nih.gov/pubmed/31662849.

64. Lopes MJ, da Silva ET, Ca J, Goncalves A, Rodrigues A, Mandjuba C, et al. Perceptions, attitudes and practices towards scabies in communities on the Bijagos Islands, Guinea-Bissau. Trans R Soc Trop Med Hyg. 2019; doi: 10.1093/trstmh/trz102. https://www.ncbi.nlm.nih.gov/pubmed/31722016.

65. Lin HC, Huang KY, Chung CH, Lin HA, Chen RM, Tsao CH, et al. Infection with Trichomonas vaginalis increases the risk of psychiatric disorders in women: a nationwide population-based cohort study. Parasit Vectors. 2019;12 1:88; doi: 10.1186/s13071-019-3350-x. https://www.ncbi.nlm.nih.gov/pubmed/30867042.

66. Leonardo L, Hernandez L, Magturo TC, Palasi W, Rubite JM, de Cadiz A, et al. Current Status of Neglected Tropical Diseases (NTDs) in the Philippines. Acta Trop. 2019:105284; doi: 10.1016/j.actatropica.2019.105284. https://www.ncbi.nlm.nih.gov/pubmed/31786109.

67. Lappan R, Classon C, Kumar S, Singh OP, de Almeida RV, Chakravarty J, et al. Meta-taxonomic analysis of prokaryotic and eukaryotic gut flora in stool samples from visceral leishmaniasis cases and endemic controls in Bihar State India. PLoS Negl Trop Dis. 2019;13 9:e0007444; doi: 10.1371/journal.pntd.0007444. https://www.ncbi.nlm.nih.gov/pubmed/31490933.

68. Lankester F, Davis A, Kinung'hi S, Yoder J, Bunga C, Alkara S, et al. An integrated health delivery platform, targeting soil-transmitted helminths (STH) and canine mediated human rabies, results in cost savings and increased breadth of treatment for STH in remote communities in Tanzania. BMC Public Health. 2019;19 1:1398; doi: 10.1186/s12889-019-7737-6. https://www.ncbi.nlm.nih.gov/pubmed/31660915.

69. Kuper H. Neglected tropical diseases and disability-what is the link? Trans R Soc Trop Med Hyg. 2019;113 12:839-44; doi: 10.1093/trstmh/trz001. https://www.ncbi.nlm.nih.gov/pubmed/30892653.

70. Kulinkina AV, Sarkar R, Mohan VR, Walz Y, Kaliappan SP, Ajjampur SSR, et al. Prediction of hookworm prevalence in southern India using environmental parameters derived from Landsat 8 remotely sensed data. Int J Parasitol. 2019; doi: 10.1016/j.ijpara.2019.10.001. https://www.ncbi.nlm.nih.gov/pubmed/31756313.

71. Krauth SJ, Balen J, Gobert GN, Lamberton PHL. A Call for Systems Epidemiology to Tackle the Complexity of Schistosomiasis, Its Control, and Its Elimination. Trop Med Infect Dis. 2019;4 1; doi: 10.3390/tropicalmed4010021. https://www.ncbi.nlm.nih.gov/pubmed/30699922.

72. Konan DO, Mosi L, Fokou G, Dassi C, Narh CA, Quaye C, et al. Buruli ulcer in southern Cote D'ivoire: dynamic schemes of perception and interpretation of modes of transmission. J Biosoc Sci. 2019;51 4:520-33; doi: 10.1017/S0021932018000317. https://www.ncbi.nlm.nih.gov/pubmed/30376901.

73. Klohe K, Amuasi J, Kaducu JM, Haavardsson I, Bogatyreva E, Onarheim KH, et al. The 2017 Oslo conference report on neglected tropical diseases and emerging/re-emerging infectious diseases - focus on populations underserved. Infect Dis Poverty. 2019;8 1:40; doi: 10.1186/s40249-019-0550-8. https://www.ncbi.nlm.nih.gov/pubmed/31138293.

74. Kibira SPS, Ssempebwa JC, Ssenyonga R, Radloff S, Makumbi FE. Schistosomiasis infection in pre-school aged children in Uganda: a qualitative descriptive study to identify routes of exposure. BMC Infect Dis. 2019;19 1:165; doi: 10.1186/s12879-019-3803-z. https://www.ncbi.nlm.nih.gov/pubmed/30764781.

75. Khan MS, Pullan R, Okello G, Nyikuri M, McKee M, Balabanova D. "For how long are we going to take the tablets?" Kenyan stakeholders' views on priority investments to sustainably tackle soil-transmitted helminths. Soc Sci Med. 2019;228:51-9; doi: 10.1016/j.socscimed.2019.02.050. https://www.ncbi.nlm.nih.gov/pubmed/30875544.

76. Khan A, Ayaz R, Mehtab A, Naz K, Haider W, Gondal MA, et al. Knowledge, attitude & practices (KAPs) regarding rabies endemicity among the community members, Pakistan. Acta Trop. 2019;200:105156; doi: 10.1016/j.actatropica.2019.105156. https://www.ncbi.nlm.nih.gov/pubmed/31491398.

77. Kasozi KI, Namayanja M, Gaithuma AK, Mahero M, Matovu E, Yamagishi J, et al. Prevalence of hemoprotozoan parasites in small ruminants along a human-livestock-wildlife interface in western Uganda. Vet Parasitol Reg Stud Reports. 2019;17:100309; doi: 10.1016/j.vprsr.2019.100309. https://www.ncbi.nlm.nih.gov/pubmed/31303220.

78. Kabir H, Hossain S. Knowledge on leprosy and its management among primary healthcare providers in two districts of Bangladesh. BMC Health Serv Res. 2019;19 1:787; doi: 10.1186/s12913-019-4525-z. https://www.ncbi.nlm.nih.gov/pubmed/31679517.

79. Jamani S, Rodriguez C, Rueda MM, Matamoros G, Canales M, Bearman G, et al. Head lice infestations in rural Honduras: the need for an integrated approach to control neglected tropical diseases. Int J Dermatol. 2019;58 5:548-56; doi: 10.1111/ijd.14331. https://www.ncbi.nlm.nih.gov/pubmed/30549003.

80. Huong LT, Hung NH, Dai DN, Tai TA, Hien VT, Satyal P, et al. Chemical Compositions and Mosquito Larvicidal Activities of Essential Oils from Piper Species Growing Wild in Central Vietnam. Molecules. 2019;24 21; doi: 10.3390/molecules24213871. https://www.ncbi.nlm.nih.gov/pubmed/31717867.

81. Hung TY, Janson S, Smith P, Legg A, Baird RW. Declining soil transmitted helminth detections in an Australian tropical region. Pathology. 2019;51 7:737-41; doi: 10.1016/j.pathol.2019.09.002. https://www.ncbi.nlm.nih.gov/pubmed/31635949.

82. Hotez PJ, Fenwick A, Molyneux DH. Collateral Benefits of Preventive Chemotherapy - Expanding the War on Neglected Tropical Diseases. N Engl J Med. 2019;380 25:2389-91; doi: 10.1056/NEJMp1900400. https://www.ncbi.nlm.nih.gov/pubmed/31067365.

83. Hotez PJ, Bottazzi ME, Bethony J, Diemert DD. Advancing the Development of a Human Schistosomiasis Vaccine. Trends Parasitol. 2019;35 2:104-8; doi: 10.1016/j.pt.2018.10.005. https://www.ncbi.nlm.nih.gov/pubmed/30455112.

84. Hotez PJ, Biritwum NK, Fenwick A, Molyneux DH, Sachs JD. Ghana: Accelerating neglected tropical disease control in a setting of economic development. PLoS Negl Trop Dis. 2019;13 1:e0007005; doi: 10.1371/journal.pntd.0007005. https://www.ncbi.nlm.nih.gov/pubmed/30653493.

85. Hotez PJ. Immunizations and vaccines: a decade of successes and reversals, and a call for 'vaccine diplomacy'. Int Health. 2019;11 5:331-3; doi: 10.1093/inthealth/ihz024. https://www.ncbi.nlm.nih.gov/pubmed/31034023.

86. Hotez PJ. The rise or fall of Neglected Tropical Diseases in East Asia Pacific. Acta Trop. 2019:105182; doi: 10.1016/j.actatropica.2019.105182. https://www.ncbi.nlm.nih.gov/pubmed/31550453.

87. Hotez PJ. Whatever happened to China's neglected tropical diseases? Infect Dis Poverty. 2019;8 1:85; doi: 10.1186/s40249-019-0598-5. https://www.ncbi.nlm.nih.gov/pubmed/31578156.

88. Hobbs EC, Trevisan C, Johansen MV, Dorny P, Gabriel S. Value of Electronic Educational Media in Combatting Parasitic Diseases. Trends Parasitol. 2019;35 3:173-6; doi: 10.1016/j.pt.2018.10.001. https://www.ncbi.nlm.nih.gov/pubmed/30360957.

89. helminths NTDMCdgos-t. Insights from quantitative analysis and mathematical modelling on the proposed WHO 2030 goals for soil-transmitted helminths. Gates Open Res. 2019;3:1632; doi: 10.12688/gatesopenres.13077.2. https://www.ncbi.nlm.nih.gov/pubmed/31819925.

90. Hazell L, Braun L, Templeton MR. Ultraviolet sensitivity of WASH (water, sanitation, and hygiene) -related helminths: A systematic review. PLoS Negl Trop Dis. 2019;13 9:e0007777; doi: 10.1371/journal.pntd.0007777. https://www.ncbi.nlm.nih.gov/pubmed/31536504.

91. Hamill LC, Haslam D, Abrahamsson S, Hill B, Dixon R, Burgess H, et al. People are neglected, not diseases: the relationship between disability and neglected tropical diseases. Trans R Soc Trop Med Hyg. 2019;113 12:829-34; doi: 10.1093/trstmh/trz036. https://www.ncbi.nlm.nih.gov/pubmed/31111941.

92. Halilu S, Iliyasu G, Hamza M, Chippaux JP, Kuznik A, Habib AG. Snakebite burden in Sub-Saharan Africa: estimates from 41 countries. Toxicon. 2019;159:1-4; doi: 10.1016/j.toxicon.2018.12.002. https://www.ncbi.nlm.nih.gov/pubmed/30594637.

93. Group NTDMCO. The World Health Organization 2030 goals for onchocerciasis: Insights and perspectives from mathematical modelling: NTD Modelling Consortium Onchocerciasis Group. Gates Open Res. 2019;3:1545; doi: 10.12688/gatesopenres.13067.1. https://www.ncbi.nlm.nih.gov/pubmed/31723729.

94. Group NTDMCS. Insights from quantitative and mathematical modelling on the proposed WHO 2030 goal for schistosomiasis. Gates Open Res. 2019;3:1517; doi: 10.12688/gatesopenres.13052.2. https://www.ncbi.nlm.nih.gov/pubmed/31701091.

95. Grau-Pujol B, Massangaie M, Cano J, Maroto C, Ndeve A, Saute F, et al. Frequency and distribution of neglected tropical diseases in Mozambique: a systematic review. Infect Dis Poverty. 2019;8 1:103; doi: 10.1186/s40249-019-0613-x. https://www.ncbi.nlm.nih.gov/pubmed/31836025.

96. Gonzales M, Baker MC, Celestino A, Santa Morillo D, Chambliss A, Adams S, et al. How lymphatic filariasis was eliminated from an urban poor setting in Santo Domingo, Dominican Republic. Int Health. 2019;11 2:108-18; doi: 10.1093/inthealth/ihy059. https://www.ncbi.nlm.nih.gov/pubmed/30285112.

97. Getaz L, Castro R, Zamora P, Kramer M, Gareca N, Torrico-Espinoza MDC, et al. Epidemiology of Strongyloides stercoralis infection in Bolivian patients at high risk of complications. PLoS Negl Trop Dis. 2019;13 1:e0007028; doi: 10.1371/journal.pntd.0007028. https://www.ncbi.nlm.nih.gov/pubmed/30653489.

98. George S, Joy TM, Kumar A, Panicker KN, George LS, Raj M, et al. Prevalence of Neglected Tropical Diseases (Leishmaniasis and Lymphatic Filariasis) and Malaria Among a Migrant Labour Settlement in Kerala, India. J Immigr Minor Health. 2019;21 3:563-9; doi: 10.1007/s10903-018-0767-9. https://www.ncbi.nlm.nih.gov/pubmed/29860672.

99. Gabriel A, Valerio-Bolas A, Palma-Marques J, Mourata-Goncalves P, Ruas P, Dias-Guerreiro T, et al. Cutaneous Leishmaniasis: The Complexity of Host's Effective Immune Response against a Polymorphic Parasitic Disease. J Immunol Res. 2019;2019:2603730; doi: 10.1155/2019/2603730. https://www.ncbi.nlm.nih.gov/pubmed/31871953.

100. Fernandez MDP, Gaspe MS, Gurtler RE. Inequalities in the social determinants of health and Chagas disease transmission risk in indigenous and creole households in the Argentine Chaco. Parasit Vectors. 2019;12 1:184; doi: 10.1186/s13071-019-3444-5. https://www.ncbi.nlm.nih.gov/pubmed/31029147.

101. Ezeh CO, Onyekwelu KC, Akinwale OP, Shan L, Wei H. Urinary schistosomiasis in Nigeria: a 50 year review of prevalence, distribution and disease burden. Parasite. 2019;26:19; doi: 10.1051/parasite/2019020. https://www.ncbi.nlm.nih.gov/pubmed/30943149.

102. Erguler K, Pontiki I, Zittis G, Proestos Y, Christodoulou V, Tsirigotakis N, et al. A climate-driven and field data-assimilated population dynamics model of sand flies. Sci Rep. 2019;9 1:2469; doi: 10.1038/s41598-019-38994-w. https://www.ncbi.nlm.nih.gov/pubmed/30792449.

103. Engelman D, Cantey PT, Marks M, Solomon AW, Chang AY, Chosidow O, et al. The public health control of scabies: priorities for research and action. Lancet. 2019;394 10192:81-92; doi: 10.1016/S0140-6736(19)31136-5. https://www.ncbi.nlm.nih.gov/pubmed/31178154.

104. Dixon MA, Braae UC, Winskill P, Walker M, Devleesschauwer B, Gabriel S, et al. Strategies for tackling Taenia solium taeniosis/cysticercosis: A systematic review and comparison of transmission models, including an assessment of the wider Taeniidae family transmission models. PLoS Negl Trop Dis. 2019;13 4:e0007301; doi: 10.1371/journal.pntd.0007301. https://www.ncbi.nlm.nih.gov/pubmed/30969966.

105. Diaz-Marrero AR, Lopez-Arencibia A, Bethencout-Estrella CJ, Cen-Pacheco F, Sifaoui I, Hernandez Creus A, et al. Antiprotozoal activities of marine polyether triterpenoids. Bioorg Chem. 2019;92:103276; doi: 10.1016/j.bioorg.2019.103276. https://www.ncbi.nlm.nih.gov/pubmed/31539745.

106. Dawaki S, Al-Mekhlafi HM, Ithoi I. The burden and epidemiology of polyparasitism among rural communities in Kano State, Nigeria. Trans R Soc Trop Med Hyg. 2019;113 4:169-82; doi: 10.1093/trstmh/try128. https://www.ncbi.nlm.nih.gov/pubmed/30551211.

107. Collaborating Group on Chagas Disease M. Insights from quantitative and mathematical modelling on the proposed WHO 2030 goals for Chagas disease. Gates Open Res. 2019;3:1539; doi: 10.12688/gatesopenres.13069.1. https://www.ncbi.nlm.nih.gov/pubmed/31781687.

108. Cohn DA, Kelly MP, Bhandari K, Zoerhoff KL, Batcho WE, Drabo F, et al. Gender equity in mass drug administration for neglected tropical diseases: data from 16 countries. Int Health. 2019;11 5:370-8; doi: 10.1093/inthealth/ihz012. https://www.ncbi.nlm.nih.gov/pubmed/30845318.

109. Chippaux JP, Massougbodji A, Habib AG. The WHO strategy for prevention and control of snakebite envenoming: a sub-Saharan Africa plan. J Venom Anim Toxins Incl Trop Dis. 2019;25:e20190083; doi: 10.1590/1678-9199-JVATITD-2019-0083. https://www.ncbi.nlm.nih.gov/pubmed/31839803.

110. Chandler DJ, Fuller LC. A Review of Scabies: An Infestation More than Skin Deep. Dermatology. 2019;235 2:79-90; doi: 10.1159/000495290. https://www.ncbi.nlm.nih.gov/pubmed/30544123.

111. Chamnanchanunt S, Svasti S, Fucharoen S, Umemura T. Neglected Tropical Diseases: The Potential Application to Monitoring by microRNAs in the Real World. Microrna. 2019; doi: 10.2174/2211536608666190620104308. https://www.ncbi.nlm.nih.gov/pubmed/31218967.

112. Chami GF, Bundy DAP. More medicines alone cannot ensure the treatment of neglected tropical diseases. Lancet Infect Dis. 2019;19 9:e330-e6; doi: 10.1016/S1473-3099(19)30160-4. https://www.ncbi.nlm.nih.gov/pubmed/31160190.

113. Brisse ME, Ly H. Hemorrhagic Fever-Causing Arenaviruses: Lethal Pathogens and Potent Immune Suppressors. Front Immunol. 2019;10:372; doi: 10.3389/fimmu.2019.00372. https://www.ncbi.nlm.nih.gov/pubmed/30918506.

114. Braae UC, Gabriel S, Trevisan C, Thomas LF, Magnussen P, Abela-Ridder B, et al. Stepwise approach for the control and eventual elimination of Taenia solium as a public health problem. BMC Infect Dis. 2019;19 1:182; doi: 10.1186/s12879-019-3812-y. https://www.ncbi.nlm.nih.gov/pubmed/30791888.

115. Bodimeade C, Marks M, Mabey D. Neglected tropical diseases: elimination and eradication. Clin Med (Lond). 2019;19 2:157-60; doi: 10.7861/clinmedicine.19-2-157. https://www.ncbi.nlm.nih.gov/pubmed/30872302.

116. Bartlett S, Haslam D, Bush S, Haddad D. Elimination through collaboration: success factors in a global consortium. Int Health. 2019;11 1:24-9; doi: 10.1093/inthealth/ihy054. https://www.ncbi.nlm.nih.gov/pubmed/30102360.

117. Bah YM, Paye J, Bah MS, Conteh A, Saffa S, Tia A, et al. Schistosomiasis in School Age Children in Sierra Leone After 6 Years of Mass Drug Administration With Praziquantel. Front Public Health. 2019;7:1; doi: 10.3389/fpubh.2019.00001. https://www.ncbi.nlm.nih.gov/pubmed/30809516.

118. Anyan WK, Abonie SD, Aboagye-Antwi F, Tettey MD, Nartey LK, Hanington PC, et al. Concurrent Schistosoma mansoni and Schistosoma haematobium infections in a peri-urban community along the Weija dam in Ghana: A wake up call for effective National Control Programme. Acta Trop. 2019;199:105116; doi: 10.1016/j.actatropica.2019.105116. https://www.ncbi.nlm.nih.gov/pubmed/31356786.

119. Al-Mekhlafi HM, Nasr NA, Lim YAL, Elyana FN, Sady H, Atroosh WM, et al. Prevalence and risk factors of Strongyloides stercoralis infection among Orang Asli schoolchildren: new insights into the epidemiology, transmission and diagnosis of strongyloidiasis in Malaysia. Parasitology. 2019;146 12:1602-14; doi: 10.1017/S0031182019000945. https://www.ncbi.nlm.nih.gov/pubmed/31303180.

120. Al Nasr I, Jentzsch J, Winter I, Schobert R, Ersfeld K, Koko WS, et al. Antiparasitic activities of new lawsone Mannich bases. Arch Pharm (Weinheim). 2019;352 11:e1900128; doi: 10.1002/ardp.201900128. https://www.ncbi.nlm.nih.gov/pubmed/31536649.

121. Akinsolu FT, Nemieboka PO, Njuguna DW, Ahadji MN, Dezso D, Varga O. Emerging Resistance of Neglected Tropical Diseases: A Scoping Review of the Literature. Int J Environ Res Public Health. 2019;16 11; doi: 10.3390/ijerph16111925. https://www.ncbi.nlm.nih.gov/pubmed/31151318.

122. Addisu A, Adriaensen W, Balew A, Asfaw M, Diro E, Garba Djirmay A, et al. Neglected tropical diseases and the sustainable development goals: an urgent call for action from the front line. BMJ Glob Health. 2019;4 1:e001334; doi: 10.1136/bmjgh-2018-001334. https://www.ncbi.nlm.nih.gov/pubmed/30899568.

123. . WHO interim guidelines for the treatment of gambiense human African trypanosomiasis. Geneva; 2019.

124. Zahedifard F, Rafati S. Prospects for antimicrobial peptide-based immunotherapy approaches in Leishmania control. Expert Rev Anti Infect Ther. 2018;16 6:461-9; doi: 10.1080/14787210.2018.1483720. https://www.ncbi.nlm.nih.gov/pubmed/29889579.

125. Yu Q, Liu H, Xiao N. Unmanned aerial vehicles: potential tools for use in zoonosis control. Infect Dis Poverty. 2018;7 1:49; doi: 10.1186/s40249-018-0430-7. https://www.ncbi.nlm.nih.gov/pubmed/29886844.

126. Yi-Ting L, Guang-Hui R, You-Sheng L, Kun Y, Le-Ping S, Shi-Zhu L, et al. [Global burden and challenges of parasitic diseases in Africa]. Zhongguo Xue Xi Chong Bing Fang Zhi Za Zhi. 2018;30 2:226-31; doi: 10.16250/j.32.1374.2018020. https://www.ncbi.nlm.nih.gov/pubmed/29770673.

127. Woode ME, Khan JAM, Thomson R, Niessen LW, Consortium C. Equity and efficiency in the scaled-up implementation of integrated neglected tropical disease control: the health economics protocol of the COUNTDOWN multicountry observational study in Ghana, Cameroon and Liberia. BMJ Open. 2018;8 6:e020113; doi: 10.1136/bmjopen-2017-020113. https://www.ncbi.nlm.nih.gov/pubmed/29961005.

128. Winkler AS, Klohe K, Schmidt V, Haavardsson I, Abraham A, Prodjinotho UF, et al. Neglected tropical diseases - the present and the future. Tidsskr Nor Laegeforen. 2018;138 3; doi: 10.4045/tidsskr.17.0678. https://www.ncbi.nlm.nih.gov/pubmed/29411594.

129. Whiteland HL, Chakroborty A, Forde-Thomas JE, Crusco A, Cookson A, Hollinshead J, et al. An Abies procera-derived tetracyclic triterpene containing a steroid-like nucleus core and a lactone side chain attenuates in vitro survival of both Fasciola hepatica and Schistosoma mansoni. Int J Parasitol Drugs Drug Resist. 2018;8 3:465-74; doi: 10.1016/j.ijpddr.2018.10.009. https://www.ncbi.nlm.nih.gov/pubmed/30399512.

130. Weng HB, Chen HX, Wang MW. Innovation in neglected tropical disease drug discovery and development. Infect Dis Poverty. 2018;7 1:67; doi: 10.1186/s40249-018-0444-1. https://www.ncbi.nlm.nih.gov/pubmed/29950174.

131. Wall RJ, Rico E, Lukac I, Zuccotto F, Elg S, Gilbert IH, et al. Clinical and veterinary trypanocidal benzoxaboroles target CPSF3. Proc Natl Acad Sci U S A. 2018;115 38:9616-21; doi: 10.1073/pnas.1807915115. https://www.ncbi.nlm.nih.gov/pubmed/30185555.

132. Vizzoni AG, Varela MC, Sangenis LHC, Hasslocher-Moreno AM, do Brasil P, Saraiva RM. Ageing with Chagas disease: an overview of an urban Brazilian cohort in Rio de Janeiro. Parasit Vectors. 2018;11 1:354; doi: 10.1186/s13071-018-2929-y. https://www.ncbi.nlm.nih.gov/pubmed/29914550.

133. Vasconcellos AG, Fonseca EFBP, Morel CM. Revisiting the concept of Innovative Developing Countries (IDCs) for its relevance to health innovation and neglected tropical diseases and for the prevention and control of epidemics. PLoS Negl Trop Dis. 2018;12 7:e0006469; doi: 10.1371/journal.pntd.0006469. https://www.ncbi.nlm.nih.gov/pubmed/30001318.

134. Turner HC, Toor J, Hollingsworth TD, Anderson RM. Economic Evaluations of Mass Drug Administration: The Importance of Economies of Scale and Scope. Clin Infect Dis. 2018;66 8:1298-303; doi: 10.1093/cid/cix1001. https://www.ncbi.nlm.nih.gov/pubmed/29126255.

135. Tsegay G, Deribe K, Deyessa N, Addissie A, Davey G, Cooper M, et al. 'I should not feed such a weak woman'. Intimate partner violence among women living with podoconiosis: A qualitative study in northern Ethiopia. PLoS One. 2018;13 12:e0207571; doi: 10.1371/journal.pone.0207571. https://www.ncbi.nlm.nih.gov/pubmed/30521548.

136. Torrecilhas AC, Xander P, Ferreira KS, Batista WL. Alternative Host Models for Testing Anti-Protozoal or Antifungal Compounds and Fungal Infection. Curr Top Med Chem. 2018;18 4:300-11; doi: 10.2174/1568026618666180412154519. https://www.ncbi.nlm.nih.gov/pubmed/29651932.

137. Tiberti N, Sanchez JC. Sleeping Sickness in the 'Omics Era. Proteomics Clin Appl. 2018;12 4:e1700041; doi: 10.1002/prca.201700041. https://www.ncbi.nlm.nih.gov/pubmed/29517161.

138. Thomaz-Soccol V, Goncalves AL, Piechnik CA, Baggio RA, Boeger WA, Buchman TL, et al. Hidden danger: Unexpected scenario in the vector-parasite dynamics of leishmaniases in the Brazil side of triple border (Argentina, Brazil and Paraguay). PLoS Negl Trop Dis. 2018;12 4:e0006336; doi: 10.1371/journal.pntd.0006336. https://www.ncbi.nlm.nih.gov/pubmed/29624586.

139. Thakur L, Singh KK, Shanker V, Negi A, Jain A, Matlashewski G, et al. Atypical leishmaniasis: A global perspective with emphasis on the Indian subcontinent. PLoS Negl Trop Dis. 2018;12 9:e0006659; doi: 10.1371/journal.pntd.0006659. https://www.ncbi.nlm.nih.gov/pubmed/30260957.

140. Tembei AM, Kengne-Ouaffo JA, Ngoh EA, John B, Nji TM, Deribe K, et al. A Comparative Analysis of Economic Cost of Podoconiosis and Leprosy on Affected Households in the Northwest Region of Cameroon. Am J Trop Med Hyg. 2018;98 4:1075-81; doi: 10.4269/ajtmh.17-0931. https://www.ncbi.nlm.nih.gov/pubmed/29460727.

141. Tangkawattana S, Sripa B. Integrative EcoHealth/One Health Approach for Sustainable Liver Fluke Control: The Lawa Model. Adv Parasitol. 2018;102:115-39; doi: 10.1016/bs.apar.2018.07.002. https://www.ncbi.nlm.nih.gov/pubmed/30442307.

142. Sun N, Amon JJ. Addressing Inequity: Neglected Tropical Diseases and Human Rights. Health Hum Rights. 2018;20 1:11-25. https://www.ncbi.nlm.nih.gov/pubmed/30008549.

143. Stroehlein AJ, Gasser RB, Hall RS, Young ND. Interactive online application for the prediction, ranking and prioritisation of drug targets in Schistosoma haematobium. Parasit Vectors. 2018;11 1:605; doi: 10.1186/s13071-018-3197-6. https://www.ncbi.nlm.nih.gov/pubmed/30482220.

144. Standley C, Boyce MR, Klineberg A, Essix G, Katz R. Organization of oversight for integrated control of neglected tropical diseases within Ministries of Health. PLoS Negl Trop Dis. 2018;12 11:e0006929; doi: 10.1371/journal.pntd.0006929. https://www.ncbi.nlm.nih.gov/pubmed/30462639.

145. Silver ZA, Kaliappan SP, Samuel P, Venugopal S, Kang G, Sarkar R, et al. Geographical distribution of soil transmitted helminths and the effects of community type in South Asia and South East Asia - A systematic review. PLoS Negl Trop Dis. 2018;12 1:e0006153; doi: 10.1371/journal.pntd.0006153. https://www.ncbi.nlm.nih.gov/pubmed/29346440.

146. Sigfrid L, Reusken C, Eckerle I, Nussenblatt V, Lipworth S, Messina J, et al. Preparing clinicians for (re-)emerging arbovirus infectious diseases in Europe. Clin Microbiol Infect. 2018;24 3:229-39; doi: 10.1016/j.cmi.2017.05.029. https://www.ncbi.nlm.nih.gov/pubmed/28648861.

147. Shott J, Ducker C, Unnasch TR, Mackenzie CD. Establishing quality assured (QA) laboratory support for onchocerciasis elimination in Africa. Int Health. 2018;10 suppl_1:i33-i9; doi: 10.1093/inthealth/ihx059. https://www.ncbi.nlm.nih.gov/pubmed/29471345.

148. Shirey RJ, Globisch D, Eubanks LM, Hixon MS, Janda KD. Noninvasive Urine Biomarker Lateral Flow Immunoassay for Monitoring Active Onchocerciasis. ACS Infect Dis. 2018;4 10:1423-31; doi: 10.1021/acsinfecdis.8b00163. https://www.ncbi.nlm.nih.gov/pubmed/30141624.

149. Shey RA, Ghogomu SM, Njume FN, Gainkam LOT, Poelvoorde P, Mutesa L, et al. Prediction and validation of the structural features of Ov58GPCR, an immunogenic determinant of Onchocerca volvulus. PLoS One. 2018;13 9:e0202915; doi: 10.1371/journal.pone.0202915. https://www.ncbi.nlm.nih.gov/pubmed/30256790.

150. Savioli L, Daumerie D. WHO leadership is essential for the elimination of NTDs. Lancet. 2018;390 10114:2765; doi: 10.1016/S0140-6736(17)33300-7. https://www.ncbi.nlm.nih.gov/pubmed/29303718.

151. Sato MO, Sato M, Yanagida T, Waikagul J, Pongvongsa T, Sako Y, et al. Taenia solium, Taenia saginata, Taenia asiatica, their hybrids and other helminthic infections occurring in a neglected tropical diseases' highly endemic area in Lao PDR. PLoS Negl Trop Dis. 2018;12 2:e0006260; doi: 10.1371/journal.pntd.0006260. https://www.ncbi.nlm.nih.gov/pubmed/29420601.

152. Sansom C. Neglected tropical diseases: securing sustainability. Lancet Infect Dis. 2018;18 5:502-3; doi: 10.1016/S1473-3099(18)30239-1. https://www.ncbi.nlm.nih.gov/pubmed/29695365.

153. Sangare MB, Coulibaly YI, Coulibaly SY, Coulibaly ME, Traore B, Dicko I, et al. A cross-sectional study of the filarial and Leishmania co-endemicity in two ecologically distinct settings in Mali. Parasit Vectors. 2018;11 1:18; doi: 10.1186/s13071-017-2531-8. https://www.ncbi.nlm.nih.gov/pubmed/29310700.

154. Salle G, Laing R, Cotton JA, Maitland K, Martinelli A, Holroyd N, et al. Transcriptomic profiling of nematode parasites surviving vaccine exposure. Int J Parasitol. 2018;48 5:395-402; doi: 10.1016/j.ijpara.2018.01.004. https://www.ncbi.nlm.nih.gov/pubmed/29534987.

155. Salguero FJ, Garcia-Jimenez WL, Lima I, Seifert K. Histopathological and immunohistochemical characterisation of hepatic granulomas in Leishmania donovani-infected BALB/c mice: a time-course study. Parasit Vectors. 2018;11 1:73; doi: 10.1186/s13071-018-2624-z. https://www.ncbi.nlm.nih.gov/pubmed/29386047.

156. Sakkas H, Bozidis P, Franks A, Papadopoulou C. Oropouche Fever: A Review. Viruses. 2018;10 4; doi: 10.3390/v10040175. https://www.ncbi.nlm.nih.gov/pubmed/29617280.

157. Romani L, Marks M, Sokana O, Nasi T, Kamoriki B, Wand H, et al. Feasibility and safety of mass drug coadministration with azithromycin and ivermectin for the control of neglected tropical diseases: a single-arm intervention trial. Lancet Glob Health. 2018;6 10:e1132-e8; doi: 10.1016/S2214-109X(18)30397-8. https://www.ncbi.nlm.nih.gov/pubmed/30223985.

158. Remenyi R, Gao Y, Hughes RE, Curd A, Zothner C, Peckham M, et al. Persistent Replication of a Chikungunya Virus Replicon in Human Cells Is Associated with Presence of Stable Cytoplasmic Granules Containing Nonstructural Protein 3. J Virol. 2018;92 16; doi: 10.1128/JVI.00477-18. https://www.ncbi.nlm.nih.gov/pubmed/29875241.

159. Reed SL, McKerrow JH. Why Funding for Neglected Tropical Diseases Should Be a Global Priority. Clin Infect Dis. 2018;67 3:323-6; doi: 10.1093/cid/ciy349. https://www.ncbi.nlm.nih.gov/pubmed/29688342.

160. Pagliusi S, Dennehy M, Kim H, Committee DAO. Vaccines, inspiring innovation in health. Vaccine. 2018;36 48:7430-7; doi: 10.1016/j.vaccine.2018.05.035. https://www.ncbi.nlm.nih.gov/pubmed/29789241.

161. Padalino G, Ferla S, Brancale A, Chalmers IW, Hoffmann KF. Combining bioinformatics, cheminformatics, functional genomics and whole organism approaches for identifying epigenetic drug targets in Schistosoma mansoni. Int J Parasitol Drugs Drug Resist. 2018;8 3:559-70; doi: 10.1016/j.ijpddr.2018.10.005. https://www.ncbi.nlm.nih.gov/pubmed/30455056.

162. Osakunor DNM, Sengeh DM, Mutapi F. Coinfections and comorbidities in African health systems: At the interface of infectious and noninfectious diseases. PLoS Negl Trop Dis. 2018;12 9:e0006711; doi: 10.1371/journal.pntd.0006711. https://www.ncbi.nlm.nih.gov/pubmed/30235205.

163. Oliveira WJ, Magalhaes FDC, Elias AMS, de Castro VN, Favero V, Lindholz CG, et al. Evaluation of diagnostic methods for the detection of intestinal schistosomiasis in endemic areas with low parasite loads: Saline gradient, Helmintex, Kato-Katz and rapid urine test. PLoS Negl Trop Dis. 2018;12 2:e0006232; doi: 10.1371/journal.pntd.0006232. https://www.ncbi.nlm.nih.gov/pubmed/29470516.

164. Oliveira AM, Lopez RVM, Dibo MR, Rodas LAC, Guirado MM, Chiaravalloti-Neto F. Dispersion of Lutzomyia longipalpis and expansion of visceral leishmaniasis in Sao Paulo State, Brazil: identification of associated factors through survival analysis. Parasit Vectors. 2018;11 1:503; doi: 10.1186/s13071-018-3084-1. https://www.ncbi.nlm.nih.gov/pubmed/30201037.

165. Okello WO, Okello AL, Inthavong P, Tiemann T, Phengsivalouk A, Devleesschauwer B, et al. Improved methods to capture the total societal benefits of zoonotic disease control: Demonstrating the cost-effectiveness of an integrated control programme for Taenia solium, soil transmitted helminths and classical swine fever in northern Lao PDR. PLoS Negl Trop Dis. 2018;12 9:e0006782; doi: 10.1371/journal.pntd.0006782. https://www.ncbi.nlm.nih.gov/pubmed/30231029.

166. Ngwasiri CA, Abanda MH, Aminde LN. Ivermectin-induced fixed drug eruption in an elderly Cameroonian: a case report. J Med Case Rep. 2018;12 1:254; doi: 10.1186/s13256-018-1801-1. https://www.ncbi.nlm.nih.gov/pubmed/30201032.

167. Munoz J, Ballester MR, Antonijoan RM, Gich I, Rodriguez M, Colli E, et al. Safety and pharmacokinetic profile of fixed-dose ivermectin with an innovative 18mg tablet in healthy adult volunteers. PLoS Negl Trop Dis. 2018;12 1:e0006020; doi: 10.1371/journal.pntd.0006020. https://www.ncbi.nlm.nih.gov/pubmed/29346388.

168. Molyneux DH, Dean L, Adekeye O, Stothard JR, Theobald S. The changing global landscape of health and disease: addressing challenges and opportunities for sustaining progress towards control and elimination of neglected tropical diseases (NTDs). Parasitology. 2018;145 13:1647-54; doi: 10.1017/S0031182018000069. https://www.ncbi.nlm.nih.gov/pubmed/29547362.

169. Mirambo MM, Mgode GF, Malima ZO, John M, Mngumi EB, Mhamphi GG, et al. Seropositivity of Brucella spp. and Leptospira spp. antibodies among abattoir workers and meat vendors in the city of Mwanza, Tanzania: A call for one health approach control strategies. PLoS Negl Trop Dis. 2018;12 6:e0006600; doi: 10.1371/journal.pntd.0006600. https://www.ncbi.nlm.nih.gov/pubmed/29939991.

170. Minet C, Thevenon S, Chantal I, Solano P, Berthier D. Mini-review on CRISPR-Cas9 and its potential applications to help controlling neglected tropical diseases caused by Trypanosomatidae. Infect Genet Evol. 2018;63:326-31; doi: 10.1016/j.meegid.2018.02.030. https://www.ncbi.nlm.nih.gov/pubmed/29486366.

171. Metz T. How to deal with neglected tropical diseases in the light of an African ethic. Dev World Bioeth. 2018;18 3:233-40; doi: 10.1111/dewb.12179. https://www.ncbi.nlm.nih.gov/pubmed/29110410.

172. Mejia A, Matamoros G, Fontecha G, Sosa-Ochoa W. Bionomic aspects of Lutzomyia evansi and Lutzomyia longipalpis, proven vectors of Leishmania infantum in an endemic area of non-ulcerative cutaneous leishmaniasis in Honduras. Parasit Vectors. 2018;11 1:15; doi: 10.1186/s13071-017-2605-7. https://www.ncbi.nlm.nih.gov/pubmed/29304878.

173. Mediannikov O, Ranque S. Mansonellosis, the most neglected human filariasis. New Microbes New Infect. 2018;26:S19-S22; doi: 10.1016/j.nmni.2018.08.016. https://www.ncbi.nlm.nih.gov/pubmed/30402239.

174. Means AR, Krentel A, Theobald S, Dean L, Mbabazi PS, Elphick-Pooley T, et al. Catalyzing NTD gender and equity research: A call for papers. PLoS Negl Trop Dis. 2018;12 10:e0006681; doi: 10.1371/journal.pntd.0006681. https://www.ncbi.nlm.nih.gov/pubmed/30335743.

175. Matilla F, Velleman Y, Harrison W, Nevel M. Animal influence on water, sanitation and hygiene measures for zoonosis control at the household level: A systematic literature review. PLoS Negl Trop Dis. 2018;12 7:e0006619; doi: 10.1371/journal.pntd.0006619. https://www.ncbi.nlm.nih.gov/pubmed/30001331.

176. Martins-Melo FR, Carneiro M, Ramos AN, Jr., Heukelbach J, Ribeiro ALP, Werneck GL. The burden of Neglected Tropical Diseases in Brazil, 1990-2016: A subnational analysis from the Global Burden of Disease Study 2016. PLoS Negl Trop Dis. 2018;12 6:e0006559; doi: 10.1371/journal.pntd.0006559. https://www.ncbi.nlm.nih.gov/pubmed/29864133.

177. Martinez-Parra AG, Pinilla-Alfonso MY, Abadia-Barrero CE. Sociocultural dynamics that influence Chagas disease health care in Colombia. Soc Sci Med. 2018;215:142-50; doi: 10.1016/j.socscimed.2018.09.012. https://www.ncbi.nlm.nih.gov/pubmed/30236829.

178. Martin D, Wiegand R, Goodhew B, Lammie P, Mkocha H, Kasubi M. Impact of Ivermectin Mass Drug Administration for Lymphatic Filariasis on Scabies in Eight Villages in Kongwa District, Tanzania. Am J Trop Med Hyg. 2018;99 4:937-9; doi: 10.4269/ajtmh.18-0018. https://www.ncbi.nlm.nih.gov/pubmed/30062986.

179. Marco-Crespo B, Casapulla S, Nieto-Sanchez C, Urrego JGG, Grijalva MJ. Youth participatory research and evaluation to inform a Chagas disease prevention program in Ecuador. Eval Program Plann. 2018;69:99-108; doi: 10.1016/j.evalprogplan.2018.04.009. https://www.ncbi.nlm.nih.gov/pubmed/29753193.

180. Madon S, Malecela MN, Mashoto K, Donohue R, Mubyazi G, Michael E. The role of community participation for sustainable integrated neglected tropical diseases and water, sanitation and hygiene intervention programs: A pilot project in Tanzania. Soc Sci Med. 2018;202:28-37; doi: 10.1016/j.socscimed.2018.02.016. https://www.ncbi.nlm.nih.gov/pubmed/29501716.

181. Mader P, Rennar GA, Ventura AMP, Grevelding CG, Schlitzer M. Chemotherapy for Fighting Schistosomiasis: Past, Present and Future. ChemMedChem. 2018;13 22:2374-89; doi: 10.1002/cmdc.201800572. https://www.ncbi.nlm.nih.gov/pubmed/30212614.

182. Lustigman S, Makepeace BL, Klei TR, Babayan SA, Hotez P, Abraham D, et al. Onchocerca volvulus: The Road from Basic Biology to a Vaccine. Trends Parasitol. 2018;34 1:64-79; doi: 10.1016/j.pt.2017.08.011. https://www.ncbi.nlm.nih.gov/pubmed/28958602.

183. Longbottom J, Shearer FM, Devine M, Alcoba G, Chappuis F, Weiss DJ, et al. Vulnerability to snakebite envenoming: a global mapping of hotspots. Lancet. 2018;392 10148:673-84; doi: 10.1016/S0140-6736(18)31224-8. https://www.ncbi.nlm.nih.gov/pubmed/30017551.

184. Lin WM, Addiss DG. Sustainable access to deworming drugs in a changing landscape. Lancet Infect Dis. 2018;18 12:e395-e8; doi: 10.1016/S1473-3099(18)30351-7. https://www.ncbi.nlm.nih.gov/pubmed/30122439.

185. Lenk EJ, Redekop WK, Luyendijk M, Fitzpatrick C, Niessen L, Stolk WA, et al. Socioeconomic benefit to individuals of achieving 2020 targets for four neglected tropical diseases controlled/eliminated by innovative and intensified disease management: Human African trypanosomiasis, leprosy, visceral leishmaniasis, Chagas disease. PLoS Negl Trop Dis. 2018;12 3:e0006250; doi: 10.1371/journal.pntd.0006250. https://www.ncbi.nlm.nih.gov/pubmed/29534061.

186. Layden AJ, Tase K, Finkelstein JL. Neglected tropical diseases and vitamin B12: a review of the current evidence. Trans R Soc Trop Med Hyg. 2018;112 10:423-35; doi: 10.1093/trstmh/try078. https://www.ncbi.nlm.nih.gov/pubmed/30165408.

187. Lakwo T, Ukety T, Bakajika D, Tukahebwa E, Awaca P, Amazigo U. "Cross-border collaboration in onchocerciasis elimination in Uganda: progress, challenges and opportunities from 2008 to 2013". Global Health. 2018;14 1:16; doi: 10.1186/s12992-018-0333-1. https://www.ncbi.nlm.nih.gov/pubmed/29409509.

188. Kugo M, Keter L, Maiyo A, Kinyua J, Ndemwa P, Maina G, et al. Fortification of Carica papaya fruit seeds to school meal snacks may aid Africa mass deworming programs: a preliminary survey. BMC Complement Altern Med. 2018;18 1:327; doi: 10.1186/s12906-018-2379-2. https://www.ncbi.nlm.nih.gov/pubmed/30526582.

189. Krolewiecki AJ. Misconceptions and paradoxes in soil-transmitted helminthiases control as a public health problem. PLoS Negl Trop Dis. 2018;12 9:e0006672; doi: 10.1371/journal.pntd.0006672. https://www.ncbi.nlm.nih.gov/pubmed/30212450.

190. Krentel A, Gyapong M, Ogundahunsi O, Amuyunzu-Nyamongo M, McFarland DA. Ensuring no one is left behind: Urgent action required to address implementation challenges for NTD control and elimination. PLoS Negl Trop Dis. 2018;12 6:e0006426; doi: 10.1371/journal.pntd.0006426. https://www.ncbi.nlm.nih.gov/pubmed/29879105.

191. Koudou BG, Kouakou MM, Ouattara AF, Yeo S, Brika P, Meite A, et al. Update on the current status of onchocerciasis in Cote d'Ivoire following 40 years of intervention: Progress and challenges. PLoS Negl Trop Dis. 2018;12 10:e0006897; doi: 10.1371/journal.pntd.0006897. https://www.ncbi.nlm.nih.gov/pubmed/30352058.

192. Khieu V, Or V, Tep C, Odermatt P, Tsuyuoka R, Char MC, et al. How elimination of lymphatic filariasis as a public health problem in the Kingdom of Cambodia was achieved. Infect Dis Poverty. 2018;7 1:15; doi: 10.1186/s40249-018-0394-7. https://www.ncbi.nlm.nih.gov/pubmed/29463307.

193. Khatonier R, Khan AM, Sarmah P, Ahmed GU. Role of IL-21 in host pathogenesis in experimental visceral leishmaniasis. J Parasit Dis. 2018;42 4:500-4; doi: 10.1007/s12639-018-1025-8. https://www.ncbi.nlm.nih.gov/pubmed/30538346.

194. Kelly-Hope LA, Hemingway J, Taylor MJ, Molyneux DH. Increasing evidence of low lymphatic filariasis prevalence in high risk Loa loa areas in Central and West Africa: a literature review. Parasit Vectors. 2018;11 1:349; doi: 10.1186/s13071-018-2900-y. https://www.ncbi.nlm.nih.gov/pubmed/29907117.

195. Kelly-Hope LA, Blundell HJ, Macfarlane CL, Molyneux DH. Innovative Surveillance Strategies to Support the Elimination of Filariasis in Africa. Trends Parasitol. 2018;34 8:694-711; doi: 10.1016/j.pt.2018.05.004. https://www.ncbi.nlm.nih.gov/pubmed/29958813.

196. Karunaweera ND, Ferreira MU. Leishmaniasis: current challenges and prospects for elimination with special focus on the South Asian region. Parasitology. 2018;145 4:425-9; doi: 10.1017/S0031182018000471. https://www.ncbi.nlm.nih.gov/pubmed/29642962.

197. Kariithi HM, Meki IK, Schneider DI, De Vooght L, Khamis FM, Geiger A, et al. Enhancing vector refractoriness to trypanosome infection: achievements, challenges and perspectives. BMC Microbiol. 2018;18 Suppl 1:179; doi: 10.1186/s12866-018-1280-y. https://www.ncbi.nlm.nih.gov/pubmed/30470182.

198. Jacobson J, Bush S. Neglected Tropical Diseases, Neglected Communities, and Conflict: How Do We Leave No One Behind? Trends Parasitol. 2018;34 3:175-7; doi: 10.1016/j.pt.2017.10.013. https://www.ncbi.nlm.nih.gov/pubmed/29162404.

199. Jacob BG, Loum D, Lakwo TL, Katholi CR, Habomugisha P, Byamukama E, et al. Community-directed vector control to supplement mass drug distribution for onchocerciasis elimination in the Madi mid-North focus of Northern Uganda. PLoS Negl Trop Dis. 2018;12 8:e0006702; doi: 10.1371/journal.pntd.0006702. https://www.ncbi.nlm.nih.gov/pubmed/30148838.

200. Ignacio CF, de Lima Barata MM, de Moraes Neto AHA. The Brazilian Family Health Strategy and the management of intestinal parasitic infections. Prim Health Care Res Dev. 2018;19 4:333-43; doi: 10.1017/S146342361700072X. https://www.ncbi.nlm.nih.gov/pubmed/29113608.

201. Ibikounle M, Onzo-Aboki A, Doritchamou J, Tougoue JJ, Boko PM, Savassi BS, et al. Results of the first mapping of soil-transmitted helminths in Benin: Evidence of countrywide hookworm predominance. PLoS Negl Trop Dis. 2018;12 3:e0006241; doi: 10.1371/journal.pntd.0006241. https://www.ncbi.nlm.nih.gov/pubmed/29494579.

202. Hurlimann E, Silue KD, Zouzou F, Ouattara M, Schmidlin T, Yapi RB, et al. Effect of an integrated intervention package of preventive chemotherapy, community-led total sanitation and health education on the prevalence of helminth and intestinal protozoa infections in Cote d'Ivoire. Parasit Vectors. 2018;11 1:115; doi: 10.1186/s13071-018-2642-x. https://www.ncbi.nlm.nih.gov/pubmed/29486790.

203. Huan-Zhang L, Xin-Zhong Z, Men-Bao Q, Jing-Bo X, Chang-Hai Z, Ying-Dan C, et al. [Current status and research progress of cysticercosis]. Zhongguo Xue Xi Chong Bing Fang Zhi Za Zhi. 2018;30 1:99-103; doi: 10.16250/j.32.1374.2018023. https://www.ncbi.nlm.nih.gov/pubmed/29536721.

204. Hotez PJ, Fenwick A, Ray SE, Hay SI, Molyneux DH. "Rapid impact" 10 years after: The first "decade" (2006-2016) of integrated neglected tropical disease control. PLoS Negl Trop Dis. 2018;12 5:e0006137; doi: 10.1371/journal.pntd.0006137. https://www.ncbi.nlm.nih.gov/pubmed/29795551.

205. Hotez PJ. The global fight to develop antipoverty vaccines in the anti-vaccine era. Hum Vaccin Immunother. 2018;14 9:2128-31; doi: 10.1080/21645515.2018.1430542. https://www.ncbi.nlm.nih.gov/pubmed/29393710.

206. Hosseini S, Oliva-Ramirez J, Vazquez-Villegas P, Rodriguez-Garcia A, Munoz-Soto RB, Aghamohammadi N, et al. Dengue Fever: A Worldwide Threat An Overview of the Infection Process, Environmental Factors for a Global Outbreak, Diagnostic Platforms and Vaccine Developments. Curr Top Med Chem. 2018;18 18:1531-49; doi: 10.2174/1568026618666181105130000. https://www.ncbi.nlm.nih.gov/pubmed/30394209.

207. Hollingsworth TD. Counting Down the 2020 Goals for 9 Neglected Tropical Diseases: What Have We Learned From Quantitative Analysis and Transmission Modeling? Clin Infect Dis. 2018;66 suppl_4:S237-S44; doi: 10.1093/cid/ciy284. https://www.ncbi.nlm.nih.gov/pubmed/29860293.

208. He X, Pan W. Role of alarmin cytokines and microRNAs in the host-schistosome interaction. F1000Res. 2018;7; doi: 10.12688/f1000research.15695.1. https://www.ncbi.nlm.nih.gov/pubmed/30345011.

209. Hazra S, Patra S. Alleviating the Neglected Tropical Diseases: Recent Developments in Diagnostics and Detection. Curr Top Med Chem. 2018;18 18:1559-74; doi: 10.2174/1568026618666181106124015. https://www.ncbi.nlm.nih.gov/pubmed/30398115.

210. Gyapong JO, Owusu IO, da-Costa Vroom FB, Mensah EO, Gyapong M. Elimination of lymphatic filariasis: current perspectives on mass drug administration. Res Rep Trop Med. 2018;9:25-33; doi: 10.2147/RRTM.S125204. https://www.ncbi.nlm.nih.gov/pubmed/30050352.

211. Gupta KK, Srivastava M, Sudan V, Singh SK, Choudhury S, Shanker D. Variation in cardiac markers and electrocardiographic alterations in young calves naturally infected with bovine tropical theileriosis. Trop Anim Health Prod. 2018;50 6:1227-30; doi: 10.1007/s11250-018-1548-0. https://www.ncbi.nlm.nih.gov/pubmed/29455427.

212. Grote A, Caffrey CR, Rebello KM, Smith D, Dalton JP, Lustigman S. Cysteine proteases during larval migration and development of helminths in their final host. PLoS Negl Trop Dis. 2018;12 8:e0005919; doi: 10.1371/journal.pntd.0005919. https://www.ncbi.nlm.nih.gov/pubmed/30138448.

213. Gazzinelli-Guimaraes AC, Gazzinelli-Guimaraes PH, Nogueira DS, Oliveira FMS, Barbosa FS, Amorim CCO, et al. IgG Induced by Vaccination With Ascaris suum Extracts Is Protective Against Infection. Front Immunol. 2018;9:2535; doi: 10.3389/fimmu.2018.02535. https://www.ncbi.nlm.nih.gov/pubmed/30473693.

214. Galvis-Ovallos F, Casanova C, Pimentel Bergamaschi D, Bianchi Galati EA. A field study of the survival and dispersal pattern of Lutzomyia longipalpis in an endemic area of visceral leishmaniasis in Brazil. PLoS Negl Trop Dis. 2018;12 4:e0006333; doi: 10.1371/journal.pntd.0006333. https://www.ncbi.nlm.nih.gov/pubmed/29608563.

215. Galgamuwa LS, Dharmaratne SD, Iddawela D. Leishmaniasis in Sri Lanka: spatial distribution and seasonal variations from 2009 to 2016. Parasit Vectors. 2018;11 1:60; doi: 10.1186/s13071-018-2647-5. https://www.ncbi.nlm.nih.gov/pubmed/29370864.

216. Franco JR, Cecchi G, Priotto G, Paone M, Diarra A, Grout L, et al. Monitoring the elimination of human African trypanosomiasis: Update to 2016. PLoS Negl Trop Dis. 2018;12 12:e0006890; doi: 10.1371/journal.pntd.0006890. https://www.ncbi.nlm.nih.gov/pubmed/30521525.

217. Filardy AA, Guimaraes-Pinto K, Nunes MP, Zukeram K, Fliess L, Pereira L, et al. Human Kinetoplastid Protozoan Infections: Where Are We Going Next? Front Immunol. 2018;9:1493; doi: 10.3389/fimmu.2018.01493. https://www.ncbi.nlm.nih.gov/pubmed/30090098.

218. Farley E, Lenglet A, Ariti C, Jiya NM, Adetunji AS, van der Kam S, et al. Risk factors for diagnosed noma in northwest Nigeria: A case-control study, 2017. PLoS Negl Trop Dis. 2018;12 8:e0006631; doi: 10.1371/journal.pntd.0006631. https://www.ncbi.nlm.nih.gov/pubmed/30138374.

219. Famakinde DO. Mosquitoes and the Lymphatic Filarial Parasites: Research Trends and Budding Roadmaps to Future Disease Eradication. Trop Med Infect Dis. 2018;3 1; doi: 10.3390/tropicalmed3010004. https://www.ncbi.nlm.nih.gov/pubmed/30274403.

220. Engelman D, Steer AC. Control Strategies for Scabies. Trop Med Infect Dis. 2018;3 3; doi: 10.3390/tropicalmed3030098. https://www.ncbi.nlm.nih.gov/pubmed/30274494.

221. Engelman D, Fuller LC, Steer AC, International Alliance for the Control of Scabies Delphi p. Consensus criteria for the diagnosis of scabies: A Delphi study of international experts. PLoS Negl Trop Dis. 2018;12 5:e0006549; doi: 10.1371/journal.pntd.0006549. https://www.ncbi.nlm.nih.gov/pubmed/29795566.

222. Eneanya OA, Cano J, Dorigatti I, Anagbogu I, Okoronkwo C, Garske T, et al. Environmental suitability for lymphatic filariasis in Nigeria. Parasit Vectors. 2018;11 1:513; doi: 10.1186/s13071-018-3097-9. https://www.ncbi.nlm.nih.gov/pubmed/30223860.

223. Emmanuel P, Dumre SP, John S, Karbwang J, Hirayama K. Mycetoma: a clinical dilemma in resource limited settings. Ann Clin Microbiol Antimicrob. 2018;17 1:35; doi: 10.1186/s12941-018-0287-4. https://www.ncbi.nlm.nih.gov/pubmed/30097030.

224. Doumbo Safiatou N, Ongoiba A, Doumtabe D, Tran Tuan M, Traore A, Sangala J, et al. [Prevalence of Malaria, Intestinal and Urinary parasite infections in Kalifabougou, Mali]. Mali Med. 2018;33 1:10-5. https://www.ncbi.nlm.nih.gov/pubmed/30484584.

225. Diaz-Albiter HM, Regnault C, Alpizar-Sosa EA, McGuinness D, Barrett M, Dillon RJ. Non-invasive visualisation and identification of fluorescent Leishmania tarentolae in infected sand flies. Wellcome Open Res. 2018;3:160; doi: 10.12688/wellcomeopenres.14910.1. https://www.ncbi.nlm.nih.gov/pubmed/30756095.

226. Di Bella S, Riccardi N, Giacobbe DR, Luzzati R. History of schistosomiasis (bilharziasis) in humans: from Egyptian medical papyri to molecular biology on mummies. Pathog Glob Health. 2018;112 5:268-73; doi: 10.1080/20477724.2018.1495357. https://www.ncbi.nlm.nih.gov/pubmed/30016215.

227. Devi RR, Raju V. Information technology in morbidity management of human lymphatic filariasis-A promising tool in global programme for elimination of lymphatic filariasis. J Vector Borne Dis. 2018;55 1:20-5; doi: 10.4103/0972-9062.234622. https://www.ncbi.nlm.nih.gov/pubmed/29916444.

228. Deribe K, Beng AA, Cano J, Njouendo AJ, Fru-Cho J, Awah AR, et al. Mapping the geographical distribution of podoconiosis in Cameroon using parasitological, serological, and clinical evidence to exclude other causes of lymphedema. PLoS Negl Trop Dis. 2018;12 1:e0006126; doi: 10.1371/journal.pntd.0006126. https://www.ncbi.nlm.nih.gov/pubmed/29324858.

229. Dean L, Njelesani J, Mulamba C, Dacombe R, Mbabazi PS, Bates I. Establishing an international laboratory network for neglected tropical diseases: Understanding existing capacity in five WHO regions. F1000Res. 2018;7:1464; doi: 10.12688/f1000research.16196.4. https://www.ncbi.nlm.nih.gov/pubmed/31119028.

230. De Neve JW, Andriantavison RL, Croke K, Krisam J, Rajoela VH, Rakotoarivony RA, et al. Health, financial, and education gains of investing in preventive chemotherapy for schistosomiasis, soil-transmitted helminthiases, and lymphatic filariasis in Madagascar: A modeling study. PLoS Negl Trop Dis. 2018;12 12:e0007002; doi: 10.1371/journal.pntd.0007002. https://www.ncbi.nlm.nih.gov/pubmed/30589847.

231. de Fuentes-Vicente JA, Gutierrez-Cabrera AE, Flores-Villegas AL, Lowenberger C, Benelli G, Salazar-Schettino PM, et al. What makes an effective Chagas disease vector? Factors underlying Trypanosoma cruzi-triatomine interactions. Acta Trop. 2018;183:23-31; doi: 10.1016/j.actatropica.2018.04.008. https://www.ncbi.nlm.nih.gov/pubmed/29625091.

232. Dacal E, Saugar JM, de Lucio A, Hernandez-de-Mingo M, Robinson E, Koster PC, et al. Prevalence and molecular characterization of Strongyloides stercoralis, Giardia duodenalis, Cryptosporidium spp., and Blastocystis spp. isolates in school children in Cubal, Western Angola. Parasit Vectors. 2018;11 1:67; doi: 10.1186/s13071-018-2640-z. https://www.ncbi.nlm.nih.gov/pubmed/29378626.

233. Da Silva Santos L, Wolff H, Chappuis F, Albajar-Vinas P, Vitoria M, Tran NT, et al. Coinfections between Persistent Parasitic Neglected Tropical Diseases and Viral Infections among Prisoners from Sub-Saharan Africa and Latin America. J Trop Med. 2018;2018:7218534; doi: 10.1155/2018/7218534. https://www.ncbi.nlm.nih.gov/pubmed/30532789.

234. Cunningham LJ, Odoom J, Pratt D, Boatemaa L, Asante-Ntim N, Attiku K, et al. Expanding molecular diagnostics of helminthiasis: Piloting use of the GPLN platform for surveillance of soil transmitted helminthiasis and schistosomiasis in Ghana. PLoS Negl Trop Dis. 2018;12 1:e0006129; doi: 10.1371/journal.pntd.0006129. https://www.ncbi.nlm.nih.gov/pubmed/29370166.

235. Cooper AJ, Hollingsworth TD. The impact of seasonality on the dynamics and control of Ascaris lumbricoides infections. J Theor Biol. 2018;453:96-107; doi: 10.1016/j.jtbi.2018.05.025. https://www.ncbi.nlm.nih.gov/pubmed/29800536.

236. Colebunders R, Basanez MG, Siling K, Post RJ, Rotsaert A, Mmbando B, et al. From river blindness control to elimination: bridge over troubled water. Infect Dis Poverty. 2018;7 1:21; doi: 10.1186/s40249-018-0406-7. https://www.ncbi.nlm.nih.gov/pubmed/29587844.

237. Cohee LM, Chilombe M, Ngwira A, Jemu SK, Mathanga DP, Laufer MK. Pilot Study of the Addition of Mass Treatment for Malaria to Existing School-Based Programs to Treat Neglected Tropical Diseases. Am J Trop Med Hyg. 2018;98 1:95-9; doi: 10.4269/ajtmh.17-0590. https://www.ncbi.nlm.nih.gov/pubmed/29141763.

238. Chandler DJ, Fuller LC. The Skin-A Common Pathway for Integrating Diagnosis and Management of NTDs. Trop Med Infect Dis. 2018;3 3; doi: 10.3390/tropicalmed3030101. https://www.ncbi.nlm.nih.gov/pubmed/30274497.

239. Castilho VVS, Goncalves KCS, Rebello KM, Baptista LPR, Sangenito LS, Santos HLC, et al. Docking simulation between HIV peptidase inhibitors and Trypanosoma cruzi aspartyl peptidase. BMC Res Notes. 2018;11 1:825; doi: 10.1186/s13104-018-3927-z. https://www.ncbi.nlm.nih.gov/pubmed/30463602.

240. Campbell SJ, Biritwum NK, Woods G, Velleman Y, Fleming F, Stothard JR. Tailoring Water, Sanitation, and Hygiene (WASH) Targets for Soil-Transmitted Helminthiasis and Schistosomiasis Control. Trends Parasitol. 2018;34 1:53-63; doi: 10.1016/j.pt.2017.09.004. https://www.ncbi.nlm.nih.gov/pubmed/29055522.

241. Calvopina M, Romero-Alvarez D, Rendon M, Takagi H, Sugiyama H. Hypolobocera guayaquilensis (Decapoda: Pseudothelphusidae): A New Crab Intermediate Host of Paragonimus mexicanus in Manabi Province, Ecuador. Korean J Parasitol. 2018;56 2:189-94; doi: 10.3347/kjp.2018.56.2.189. https://www.ncbi.nlm.nih.gov/pubmed/29742874.

242. Calderon-Anyosa R, Galvez-Petzoldt C, Garcia PJ, Carcamo CP. Housing Characteristics and Leishmaniasis: A Systematic Review. Am J Trop Med Hyg. 2018;99 6:1547-54; doi: 10.4269/ajtmh.18-0037. https://www.ncbi.nlm.nih.gov/pubmed/30382013.

243. Buyon L, Slaven R, Emerson PM, King J, Debrah O, Aboe A, et al. Achieving the endgame: Integrated NTD case searches. PLoS Negl Trop Dis. 2018;12 12:e0006623; doi: 10.1371/journal.pntd.0006623. https://www.ncbi.nlm.nih.gov/pubmed/30571758.

244. Burza S, Croft SL, Boelaert M. Leishmaniasis. Lancet. 2018;392 10151:951-70; doi: 10.1016/S0140-6736(18)31204-2. https://www.ncbi.nlm.nih.gov/pubmed/30126638.

245. Buisson Y. [Scientific Day of the Societe de pathologie exotique. Elimination of Neglected Tropical Diseases: a Francophone Vision?]. Bull Soc Pathol Exot. 2018;111 3:183-8; doi: 10.3166/bspe-2018-0031. https://www.ncbi.nlm.nih.gov/pubmed/30793569.

246. Braun L, Grimes JET, Templeton MR. The effectiveness of water treatment processes against schistosome cercariae: A systematic review. PLoS Negl Trop Dis. 2018;12 4:e0006364; doi: 10.1371/journal.pntd.0006364. https://www.ncbi.nlm.nih.gov/pubmed/29608589.

247. Booth M, Clements A. Neglected Tropical Disease Control - The Case for Adaptive, Location-specific Solutions. Trends Parasitol. 2018;34 4:272-82; doi: 10.1016/j.pt.2018.02.001. https://www.ncbi.nlm.nih.gov/pubmed/29500033.

248. Bi K, Chen Y, Zhao S, Kuang Y, John Wu CH. Current Visceral Leishmaniasis Research: A Research Review to Inspire Future Study. Biomed Res Int. 2018;2018:9872095; doi: 10.1155/2018/9872095. https://www.ncbi.nlm.nih.gov/pubmed/30105272.

249. Bharti B, Bharti S, Khurana S. Worm Infestation: Diagnosis, Treatment and Prevention. Indian J Pediatr. 2018;85 11:1017-24; doi: 10.1007/s12098-017-2505-z. https://www.ncbi.nlm.nih.gov/pubmed/29127616.

250. Bennuru S, O'Connell EM, Drame PM, Nutman TB. Mining Filarial Genomes for Diagnostic and Therapeutic Targets. Trends Parasitol. 2018;34 1:80-90; doi: 10.1016/j.pt.2017.09.003. https://www.ncbi.nlm.nih.gov/pubmed/29031509.

251. Beesley NJ, Caminade C, Charlier J, Flynn RJ, Hodgkinson JE, Martinez-Moreno A, et al. Fasciola and fasciolosis in ruminants in Europe: Identifying research needs. Transbound Emerg Dis. 2018;65 Suppl 1:199-216; doi: 10.1111/tbed.12682. https://www.ncbi.nlm.nih.gov/pubmed/28984428.

252. Barogui YT, Diez G, Anagonou E, Johnson RC, Gomido IC, Amoukpo H, et al. Integrated approach in the control and management of skin neglected tropical diseases in Lalo, Benin. PLoS Negl Trop Dis. 2018;12 6:e0006584; doi: 10.1371/journal.pntd.0006584. https://www.ncbi.nlm.nih.gov/pubmed/29939988.

253. Bardosh KL. Towards a science of global health delivery: A socio-anthropological framework to improve the effectiveness of neglected tropical disease interventions. PLoS Negl Trop Dis. 2018;12 7:e0006537; doi: 10.1371/journal.pntd.0006537. https://www.ncbi.nlm.nih.gov/pubmed/30024887.

254. Ballesteros C, Geary JF, Mackenzie CD, Geary TG. Characterization of Divalent Metal Transporter 1 (DMT1) in Brugia malayi suggests an intestinal-associated pathway for iron absorption. Int J Parasitol Drugs Drug Resist. 2018;8 2:341-9; doi: 10.1016/j.ijpddr.2018.06.003. https://www.ncbi.nlm.nih.gov/pubmed/29957332.

255. Bakhiet SM, Fahal AH, Musa AM, Mohamed ESW, Omer RF, Ahmed ES, et al. A holistic approach to the mycetoma management. PLoS Negl Trop Dis. 2018;12 5:e0006391; doi: 10.1371/journal.pntd.0006391. https://www.ncbi.nlm.nih.gov/pubmed/29746460.

256. Aye NN, Lin Z, Lon KN, Linn NYY, Nwe TW, Mon KM, et al. Mapping and modelling the impact of mass drug adminstration on filariasis prevalence in Myanmar. Infect Dis Poverty. 2018;7 1:56; doi: 10.1186/s40249-018-0420-9. https://www.ncbi.nlm.nih.gov/pubmed/29855355.

257. Awah PK, Boock AU, Mou F, Koin JT, Anye EM, Noumen D, et al. Developing a Buruli ulcer community of practice in Bankim, Cameroon: A model for Buruli ulcer outreach in Africa. PLoS Negl Trop Dis. 2018;12 3:e0006238; doi: 10.1371/journal.pntd.0006238. https://www.ncbi.nlm.nih.gov/pubmed/29584724.

258. Atcheson E, Bauza K, Salman AM, Alves E, Blight J, Viveros-Sandoval ME, et al. Tailoring a Plasmodium vivax Vaccine To Enhance Efficacy through a Combination of a CSP Virus-Like Particle and TRAP Viral Vectors. Infect Immun. 2018;86 9; doi: 10.1128/IAI.00114-18. https://www.ncbi.nlm.nih.gov/pubmed/29986894.

259. Arnold BF, Scobie HM, Priest JW, Lammie PJ. Integrated Serologic Surveillance of Population Immunity and Disease Transmission. Emerg Infect Dis. 2018;24 7:1188-94; doi: 10.3201/eid2407.171928. https://www.ncbi.nlm.nih.gov/pubmed/29912680.

260. Arndt MB, Walson JL. Enteric infection and dysfunction-A new target for PLOS Neglected Tropical Diseases. PLoS Negl Trop Dis. 2018;12 12:e0006906; doi: 10.1371/journal.pntd.0006906. https://www.ncbi.nlm.nih.gov/pubmed/30592716.

261. Allan ERO, Gourbal B, Dores CB, Portet A, Bayne CJ, Blouin MS. Clearance of schistosome parasites by resistant genotypes at a single genomic region in Biomphalaria glabrata snails involves cellular components of the hemolymph. Int J Parasitol. 2018;48 5:387-93; doi: 10.1016/j.ijpara.2017.08.008. https://www.ncbi.nlm.nih.gov/pubmed/29137971.

262. Al-Bajalan MMM, Al-Jaf SMA, Niranji SS, Abdulkareem DR, Al-Kayali KK, Kato H. An outbreak of Leishmania major from an endemic to a non-endemic region posed a public health threat in Iraq from 2014-2017: Epidemiological, molecular and phylogenetic studies. PLoS Negl Trop Dis. 2018;12 3:e0006255; doi: 10.1371/journal.pntd.0006255. https://www.ncbi.nlm.nih.gov/pubmed/29494612.

263. Ahorlu CSK, Okyere D, Ampadu E. Implementing active community-based surveillance-response system for Buruli ulcer early case detection and management in Ghana. PLoS Negl Trop Dis. 2018;12 9:e0006776; doi: 10.1371/journal.pntd.0006776. https://www.ncbi.nlm.nih.gov/pubmed/30208037.

264. Agyemang ANO, Badu K, Baffour-Awuah S, Owusu-Dabo E, Biritwum NK, Garms R, et al. Evaluation of onchocerciasis control in the Upper Denkyira East municipal in the forest area of Ghana: Responses of participants and distributors to the CDTI programme. Acta Trop. 2018;185:357-62; doi: 10.1016/j.actatropica.2018.06.017. https://www.ncbi.nlm.nih.gov/pubmed/29932933.

265. Abbas M, Aloudat T, Bartolomei J, Carballo M, Durieux-Paillard S, Gabus L, et al. Migrant and refugee populations: a public health and policy perspective on a continuing global crisis. Antimicrob Resist Infect Control. 2018;7:113; doi: 10.1186/s13756-018-0403-4. https://www.ncbi.nlm.nih.gov/pubmed/30250735.

266. Yoshioka K, Tercero D, Perez B, Nakamura J, Perez L. Implementing a vector surveillance-response system for chagas disease control: a 4-year field trial in Nicaragua. Infect Dis Poverty. 2017;6 1:18; doi: 10.1186/s40249-016-0225-7. https://www.ncbi.nlm.nih.gov/pubmed/28260529.

267. Wu HW, Ito A, Ai L, Zhou XN, Acosta LP, Lee Willingham A, III. Cysticercosis/taeniasis endemicity in Southeast Asia: Current status and control measures. Acta Trop. 2017;165:121-32; doi: 10.1016/j.actatropica.2016.01.013. https://www.ncbi.nlm.nih.gov/pubmed/26802488.

268. Welburn SC, Coleman PG, Zinsstag J. Rabies Control: Could Innovative Financing Break the Deadlock? Front Vet Sci. 2017;4:32; doi: 10.3389/fvets.2017.00032. https://www.ncbi.nlm.nih.gov/pubmed/28337440.

269. Weerakoon KG, Gordon CA, Williams GM, Cai P, Gobert GN, Olveda RM, et al. Droplet Digital PCR Diagnosis of Human Schistosomiasis: Parasite Cell-Free DNA Detection in Diverse Clinical Samples. J Infect Dis. 2017;216 12:1611-22; doi: 10.1093/infdis/jix521. https://www.ncbi.nlm.nih.gov/pubmed/29029307.

270. Watts C. Neglected tropical diseases: A DFID perspective. PLoS Negl Trop Dis. 2017;11 4:e0005492; doi: 10.1371/journal.pntd.0005492. https://www.ncbi.nlm.nih.gov/pubmed/28426666.

271. Wang X, Wang W, Wang P. Long-term effectiveness of the integrated schistosomiasis control strategy with emphasis on infectious source control in China: a 10-year evaluation from 2005 to 2014. Parasitol Res. 2017;116 2:521-8; doi: 10.1007/s00436-016-5315-8. https://www.ncbi.nlm.nih.gov/pubmed/27812902.

272. Wang W, Chen J, Sheng HF, Wang NN, Yang P, Zhou XN, et al. Infectious Diseases of Poverty, the first five years. Infect Dis Poverty. 2017;6 1:96; doi: 10.1186/s40249-017-0310-6. https://www.ncbi.nlm.nih.gov/pubmed/28472981.

273. Walker M, Stolk WA, Dixon MA, Bottomley C, Diawara L, Traore MO, et al. Modelling the elimination of river blindness using long-term epidemiological and programmatic data from Mali and Senegal. Epidemics. 2017;18:4-15; doi: 10.1016/j.epidem.2017.02.005. https://www.ncbi.nlm.nih.gov/pubmed/28279455.

274. Waite RC, Woods G, Velleman Y, Freeman MC. Collaborating to develop joint water, sanitation and hygiene (WASH) and neglected tropical disease (NTD) sector monitoring: an expert consultation. Int Health. 2017;9 4:215-25; doi: 10.1093/inthealth/ihx008. https://www.ncbi.nlm.nih.gov/pubmed/28407112.

275. Van Leuvenhaege C, Vandelannoote K, Affolabi D, Portaels F, Sopoh G, de Jong BC, et al. Bacterial diversity in Buruli ulcer skin lesions: Challenges in the clinical microbiome analysis of a skin disease. PLoS One. 2017;12 7:e0181994; doi: 10.1371/journal.pone.0181994. https://www.ncbi.nlm.nih.gov/pubmed/28750103.

276. van de Burgwal LHM, Neevel AMG, Pittens C, Osterhaus A, Rupprecht CE, Claassen E. Barriers to innovation in human rabies prophylaxis and treatment: A causal analysis of insights from key opinion leaders and literature. Zoonoses Public Health. 2017;64 8:599-611; doi: 10.1111/zph.12352. https://www.ncbi.nlm.nih.gov/pubmed/28318148.

277. Truscott JE, Gurarie D, Alsallaq R, Toor J, Yoon N, Farrell SH, et al. A comparison of two mathematical models of the impact of mass drug administration on the transmission and control of schistosomiasis. Epidemics. 2017;18:29-37; doi: 10.1016/j.epidem.2017.02.003. https://www.ncbi.nlm.nih.gov/pubmed/28279453.

278. Tibayrenc M, Ayala FJ. Relevant units of analysis for applied and basic research dealing with neglected transmissible diseases: The predominant clonal evolution model of pathogenic microorganisms. PLoS Negl Trop Dis. 2017;11 4:e0005293; doi: 10.1371/journal.pntd.0005293. https://www.ncbi.nlm.nih.gov/pubmed/28448491.

279. Thomas BC, Kollie K, Koudou B, Mackenzie C. Commentary: restarting NTD programme activities after the Ebola outbreak in Liberia. Infect Dis Poverty. 2017;6 1:52; doi: 10.1186/s40249-017-0272-8. https://www.ncbi.nlm.nih.gov/pubmed/28457226.

280. Theobald S, MacPherson EE, Dean L, Jacobson J, Ducker C, Gyapong M, et al. 20 years of gender mainstreaming in health: lessons and reflections for the neglected tropical diseases community. BMJ Glob Health. 2017;2 4:e000512; doi: 10.1136/bmjgh-2017-000512. https://www.ncbi.nlm.nih.gov/pubmed/29177100.

281. Tajebe F, Getahun M, Adem E, Hailu A, Lemma M, Fikre H, et al. Disease severity in patients with visceral leishmaniasis is not altered by co-infection with intestinal parasites. PLoS Negl Trop Dis. 2017;11 7:e0005727; doi: 10.1371/journal.pntd.0005727. https://www.ncbi.nlm.nih.gov/pubmed/28732017.

282. Stothard JR, Kabatereine NB, Archer J, Al-Shehri H, Tchuem-Tchuente LA, Gyapong M, et al. A centenary of Robert T. Leiper's lasting legacy on schistosomiasis and a COUNTDOWN on control of neglected tropical diseases. Parasitology. 2017;144 12:1602-12; doi: 10.1017/S0031182016000998. https://www.ncbi.nlm.nih.gov/pubmed/27363810.

283. Stecher CW, Sacko M, Madsen H, Wilson S, Wejse C, Keita AD, et al. Anemia and growth retardation associated with Schistosoma haematobium infection in Mali: a possible subtle impact of a neglected tropical disease. Trans R Soc Trop Med Hyg. 2017;111 4:144-53; doi: 10.1093/trstmh/trx037. https://www.ncbi.nlm.nih.gov/pubmed/28673023.

284. Stanton MC. The Role of Spatial Statistics in the Control and Elimination of Neglected Tropical Diseases in Sub-Saharan Africa: A Focus on Human African Trypanosomiasis, Schistosomiasis and Lymphatic Filariasis. Adv Parasitol. 2017;97:187-241; doi: 10.1016/bs.apar.2017.01.001. https://www.ncbi.nlm.nih.gov/pubmed/28325371.

285. Spear RC. Review of "Mathematical Models for Neglected Tropical Diseases: Essential Tools for Control and Elimination, Part B" Edited by Maria-Gloria Basanez and Roy M. Anderson. Parasit Vectors. 2017;10 1:38; doi: 10.1186/s13071-017-1974-2. https://www.ncbi.nlm.nih.gov/pubmed/28109302.

286. Song L, Wu X, Ning A, Wu Z. Lessons from a 15-year-old boy with advanced schistosomiasis japonica in China: a case report. Parasitol Res. 2017;116 7:1787-91; doi: 10.1007/s00436-017-5473-3. https://www.ncbi.nlm.nih.gov/pubmed/28508167.

287. Soe HZ, Oo CC, Myat TO, Maung NS. Detection of Schistosoma Antibodies and exploration of associated factors among local residents around Inlay Lake, Southern Shan State, Myanmar. Infect Dis Poverty. 2017;6 1:3; doi: 10.1186/s40249-016-0211-0. https://www.ncbi.nlm.nih.gov/pubmed/28245867.

288. Smitherman S, Hammond T, Goldberg D, Horney J. Developing a CASPER Survey to Assess the Prevalence of Risk Factors for Neglected Tropical Diseases in Texas. Health Secur. 2017;15 3:238-43; doi: 10.1089/hs.2016.0075. https://www.ncbi.nlm.nih.gov/pubmed/28636445.

289. Smith ME, Singh BK, Irvine MA, Stolk WA, Subramanian S, Hollingsworth TD, et al. Predicting lymphatic filariasis transmission and elimination dynamics using a multi-model ensemble framework. Epidemics. 2017;18:16-28; doi: 10.1016/j.epidem.2017.02.006. https://www.ncbi.nlm.nih.gov/pubmed/28279452.

290. Shiff C. Why reinvent the wheel? Lessons in schistosomiasis control from the past. PLoS Negl Trop Dis. 2017;11 10:e0005812; doi: 10.1371/journal.pntd.0005812. https://www.ncbi.nlm.nih.gov/pubmed/29073138.

291. Schwarz NG, Loderstaedt U, Hahn A, Hinz R, Zautner AE, Eibach D, et al. Microbiological laboratory diagnostics of neglected zoonotic diseases (NZDs). Acta Trop. 2017;165:40-65; doi: 10.1016/j.actatropica.2015.09.003. https://www.ncbi.nlm.nih.gov/pubmed/26391646.

292. Savioli L, Albonico M, Colley DG, Correa-Oliveira R, Fenwick A, Green W, et al. Building a global schistosomiasis alliance: an opportunity to join forces to fight inequality and rural poverty. Infect Dis Poverty. 2017;6 1:65; doi: 10.1186/s40249-017-0280-8. https://www.ncbi.nlm.nih.gov/pubmed/28330495.

293. Saha K, Gomes A. Russell's viper venom induced nephrotoxicity, myotoxicity, and hepatotoxicity-Neutralization with gold nanoparticle conjugated 2-hydroxy-4-methoxy benzoic acid in vivo. Indian J Exp Biol. 2017;55 1:7-14. https://www.ncbi.nlm.nih.gov/pubmed/30183223.

294. Rock KS, Pandey A, Ndeffo-Mbah ML, Atkins KE, Lumbala C, Galvani A, et al. Data-driven models to predict the elimination of sleeping sickness in former Equateur province of DRC. Epidemics. 2017;18:101-12; doi: 10.1016/j.epidem.2017.01.006. https://www.ncbi.nlm.nih.gov/pubmed/28279451.

295. Ritter M, Tamadaho RS, Feid J, Vogel W, Wiszniewsky K, Perner S, et al. IL-4/5 signalling plays an important role during Litomosoides sigmodontis infection, influencing both immune system regulation and tissue pathology in the thoracic cavity. Int J Parasitol. 2017;47 14:951-60; doi: 10.1016/j.ijpara.2017.06.009. https://www.ncbi.nlm.nih.gov/pubmed/28859850.

296. Redekop WK, Lenk EJ, Luyendijk M, Fitzpatrick C, Niessen L, Stolk WA, et al. The Socioeconomic Benefit to Individuals of Achieving the 2020 Targets for Five Preventive Chemotherapy Neglected Tropical Diseases. PLoS Negl Trop Dis. 2017;11 1:e0005289; doi: 10.1371/journal.pntd.0005289. https://www.ncbi.nlm.nih.gov/pubmed/28103243.

297. Rebollo MP, Bockarie MJ. Can Lymphatic Filariasis Be Eliminated by 2020? Trends Parasitol. 2017;33 2:83-92; doi: 10.1016/j.pt.2016.09.009. https://www.ncbi.nlm.nih.gov/pubmed/27765440.

298. Rasamoelina T, Raharolahy O, Rakotozandrindrainy N, Ranaivo I, Andrianarison M, Rakotonirina B, et al. Chromoblastomycosis and sporotrichosis, two endemic but neglected fungal infections in Madagascar. J Mycol Med. 2017;27 3:312-24; doi: 10.1016/j.mycmed.2017.08.003. https://www.ncbi.nlm.nih.gov/pubmed/28847419.

299. Qian MB. Neglected tropical diseases and global burden of disease in China. Infect Dis Poverty. 2017;6 1:25; doi: 10.1186/s40249-017-0237-y. https://www.ncbi.nlm.nih.gov/pubmed/28153050.

300. Preston S, Jiao Y, Baell JB, Keiser J, Crawford S, Koehler AV, et al. Screening of the 'Open Scaffolds' collection from Compounds Australia identifies a new chemical entity with anthelmintic activities against different developmental stages of the barber's pole worm and other parasitic nematodes. Int J Parasitol Drugs Drug Resist. 2017;7 3:286-94; doi: 10.1016/j.ijpddr.2017.05.004. https://www.ncbi.nlm.nih.gov/pubmed/28732272.

301. Piseddu T, Brundu D, Stegel G, Loi F, Rolesu S, Masu G, et al. The disease burden of human cystic echinococcosis based on HDRs from 2001 to 2014 in Italy. PLoS Negl Trop Dis. 2017;11 7:e0005771; doi: 10.1371/journal.pntd.0005771. https://www.ncbi.nlm.nih.gov/pubmed/28746395.

302. Pinsent A, Gambhir M. Improving our forecasts for trachoma elimination: What else do we need to know? PLoS Negl Trop Dis. 2017;11 2:e0005378; doi: 10.1371/journal.pntd.0005378. https://www.ncbi.nlm.nih.gov/pubmed/28182664.

303. Pennington PM, Juarez JG, Arrivillaga MR, De Urioste-Stone SM, Doktor K, Bryan JP, et al. Towards Chagas disease elimination: Neonatal screening for congenital transmission in rural communities. PLoS Negl Trop Dis. 2017;11 9:e0005783; doi: 10.1371/journal.pntd.0005783. https://www.ncbi.nlm.nih.gov/pubmed/28892479.

304. Peeling RW, Boeras DI, Nkengasong J. Re-imagining the future of diagnosis of Neglected Tropical Diseases. Comput Struct Biotechnol J. 2017;15:271-4; doi: 10.1016/j.csbj.2017.02.003. https://www.ncbi.nlm.nih.gov/pubmed/28352456.

305. Pando-Robles V, Batista CV. Aedes-Borne Virus-Mosquito Interactions: Mass Spectrometry Strategies and Findings. Vector Borne Zoonotic Dis. 2017;17 6:361-75; doi: 10.1089/vbz.2016.2040. https://www.ncbi.nlm.nih.gov/pubmed/28192064.

306. Ortu G, Williams O. Neglected tropical diseases: exploring long term practical approaches to achieve sustainable disease elimination and beyond. Infect Dis Poverty. 2017;6 1:147; doi: 10.1186/s40249-017-0361-8. https://www.ncbi.nlm.nih.gov/pubmed/28950893.

307. Omura S, Crump A. Ivermectin and malaria control. Malar J. 2017;16 1:172; doi: 10.1186/s12936-017-1825-9. https://www.ncbi.nlm.nih.gov/pubmed/28438169.

308. Omondi D, Masiga DK, Fielding BC, Kariuki E, Ajamma YU, Mwamuye MM, et al. Molecular Detection of Tick-Borne Pathogen Diversities in Ticks from Livestock and Reptiles along the Shores and Adjacent Islands of Lake Victoria and Lake Baringo, Kenya. Front Vet Sci. 2017;4:73; doi: 10.3389/fvets.2017.00073. https://www.ncbi.nlm.nih.gov/pubmed/28620610.

309. Okello AL, Thomas LF. Human taeniasis: current insights into prevention and management strategies in endemic countries. Risk Manag Healthc Policy. 2017;10:107-16; doi: 10.2147/RMHP.S116545. https://www.ncbi.nlm.nih.gov/pubmed/28615981.

310. Okello AL, Thomas L, Inthavong P, Ash A, Khamlome B, Keokamphet C, et al. Reprint of "Assessing the impact of a joint human-porcine intervention package for Taenia solium control: Results of a pilot study from northern Lao PDR". Acta Trop. 2017;165:261-7; doi: 10.1016/j.actatropica.2016.11.010. https://www.ncbi.nlm.nih.gov/pubmed/27887694.

311. O'Hara GA, McNaughton AL, Maponga T, Jooste P, Ocama P, Chilengi R, et al. Hepatitis B virus infection as a neglected tropical disease. PLoS Negl Trop Dis. 2017;11 10:e0005842; doi: 10.1371/journal.pntd.0005842. https://www.ncbi.nlm.nih.gov/pubmed/28981505.

312. Nyundo AA, Munisi DZ, Gesase AP. Prevalence and Correlates of Intestinal Parasites among Patients Admitted to Mirembe National Mental Health Hospital, Dodoma, Tanzania. J Parasitol Res. 2017;2017:5651717; doi: 10.1155/2017/5651717. https://www.ncbi.nlm.nih.gov/pubmed/28611925.

313. Njim T, Aminde LN. An appraisal of the neglected tropical diseases control program in Cameroon: the case of the national program against onchocerciasis. BMC Public Health. 2017;17 1:103; doi: 10.1186/s12889-017-4037-x. https://www.ncbi.nlm.nih.gov/pubmed/28109269.

314. Netshikweta R, Garira W. A Multiscale Model for the World's First Parasitic Disease Targeted for Eradication: Guinea Worm Disease. Comput Math Methods Med. 2017;2017:1473287; doi: 10.1155/2017/1473287. https://www.ncbi.nlm.nih.gov/pubmed/28808479.

315. Negussu N, Mengistu B, Kebede B, Deribe K, Ejigu E, Tadesse G, et al. Ethiopia Schistosomiasis and Soil-Transmitted Helminthes Control Programme: Progress and Prospects. Ethiop Med J. 2017;55 Suppl 1:75-80. https://www.ncbi.nlm.nih.gov/pubmed/28878432.

316. Nana-Djeunga HC, Tchouakui M, Njitchouang GR, Tchatchueng-Mbougua JB, Nwane P, Domche A, et al. First evidence of lymphatic filariasis transmission interruption in Cameroon: Progress towards elimination. PLoS Negl Trop Dis. 2017;11 6:e0005633; doi: 10.1371/journal.pntd.0005633. https://www.ncbi.nlm.nih.gov/pubmed/28662054.

317. Mota FB, Fonseca B, Galina AC, Silva RMD. Mapping the dengue scientific landscape worldwide: a bibliometric and network analysis. Mem Inst Oswaldo Cruz. 2017;112 5:354-63; doi: 10.1590/0074-02760160423. https://www.ncbi.nlm.nih.gov/pubmed/28443981.

318. Moser W, Labhardt ND, Cheleboi M, Muhairwe J, Keiser J. Unexpected low soil-transmitted helminth prevalence in the Butha-Buthe district in Lesotho, results from a cross-sectional survey. Parasit Vectors. 2017;10 1:72; doi: 10.1186/s13071-017-1995-x. https://www.ncbi.nlm.nih.gov/pubmed/28179008.

319. Moreno E, Schwartz J, Calvo A, Blanco L, Larrea E, Irache JM, et al. Skin vaccination using microneedles coated with a plasmid DNA cocktail encoding nucleosomal histones of Leishmania spp. Int J Pharm. 2017;533 1:236-44; doi: 10.1016/j.ijpharm.2017.09.055. https://www.ncbi.nlm.nih.gov/pubmed/28964902.

320. Molyneux DH, Savioli L, Engels D. Neglected tropical diseases: progress towards addressing the chronic pandemic. Lancet. 2017;389 10066:312-25; doi: 10.1016/S0140-6736(16)30171-4. https://www.ncbi.nlm.nih.gov/pubmed/27639954.

321. Molloy SF, Chiller T, Greene GS, Burry J, Govender NP, Kanyama C, et al. Cryptococcal meningitis: A neglected NTD? PLoS Negl Trop Dis. 2017;11 6:e0005575; doi: 10.1371/journal.pntd.0005575. https://www.ncbi.nlm.nih.gov/pubmed/28662028.

322. Mitra AK, Mawson AR. Neglected Tropical Diseases: Epidemiology and Global Burden. Trop Med Infect Dis. 2017;2 3; doi: 10.3390/tropicalmed2030036. https://www.ncbi.nlm.nih.gov/pubmed/30270893.

323. Mitja O, Marks M, Bertran L, Kollie K, Argaw D, Fahal AH, et al. Integrated Control and Management of Neglected Tropical Skin Diseases. PLoS Negl Trop Dis. 2017;11 1:e0005136; doi: 10.1371/journal.pntd.0005136. https://www.ncbi.nlm.nih.gov/pubmed/28103250.

324. Michael E, Singh BK, Mayala BK, Smith ME, Hampton S, Nabrzyski J. Continental-scale, data-driven predictive assessment of eliminating the vector-borne disease, lymphatic filariasis, in sub-Saharan Africa by 2020. BMC Med. 2017;15 1:176; doi: 10.1186/s12916-017-0933-2. https://www.ncbi.nlm.nih.gov/pubmed/28950862.

325. Michael E, Madon S. Socio-ecological dynamics and challenges to the governance of Neglected Tropical Disease control. Infect Dis Poverty. 2017;6 1:35; doi: 10.1186/s40249-016-0235-5. https://www.ncbi.nlm.nih.gov/pubmed/28166826.

326. Meymandi SK, Forsyth CJ, Soverow J, Hernandez S, Sanchez D, Montgomery SP, et al. Prevalence of Chagas Disease in the Latin American-born Population of Los Angeles. Clin Infect Dis. 2017;64 9:1182-8; doi: 10.1093/cid/cix064. https://www.ncbi.nlm.nih.gov/pubmed/28329123.

327. Mehmood K, Zhang H, Sabir AJ, Abbas RZ, Ijaz M, Durrani AZ, et al. A review on epidemiology, global prevalence and economical losses of fasciolosis in ruminants. Microb Pathog. 2017;109:253-62; doi: 10.1016/j.micpath.2017.06.006. https://www.ncbi.nlm.nih.gov/pubmed/28602837.

328. McAtee CP, Seid CA, Hammond M, Hudspeth E, Keegan BP, Liu Z, et al. Expression, purification, immunogenicity and protective efficacy of a recombinant nucleoside hydrolase from Leishmania donovani, a vaccine candidate for preventing cutaneous leishmaniasis. Protein Expr Purif. 2017;130:129-36; doi: 10.1016/j.pep.2016.10.008. https://www.ncbi.nlm.nih.gov/pubmed/27773761.

329. Maxmen A. Global coalition chips away at neglected tropical diseases. Nature. 2017;544 7650:281-2; doi: 10.1038/544281a. https://www.ncbi.nlm.nih.gov/pubmed/28426009.

330. Marks M, Mitja O, Fitzpatrick C, Asiedu K, Solomon AW, Mabey DC, et al. Mathematical Modeling of Programmatic Requirements for Yaws Eradication. Emerg Infect Dis. 2017;23 1:22-8; doi: 10.3201/eid2301.160487. https://www.ncbi.nlm.nih.gov/pubmed/27983500.

331. Lu Y, Gonzales G, Chen SH, Li H, Cai YC, Chu YH, et al. Urgent needs in fostering neglected tropical diseases (NTDs) laboratory capacity in WHO Western Pacific Region: results from the external quality assessment on NTDs diagnosis in 2012-2015. Infect Dis Poverty. 2017;6 1:106; doi: 10.1186/s40249-017-0319-x. https://www.ncbi.nlm.nih.gov/pubmed/28592266.

332. Lorenz C, Azevedo TS, Virginio F, Aguiar BS, Chiaravalloti-Neto F, Suesdek L. Impact of environmental factors on neglected emerging arboviral diseases. PLoS Negl Trop Dis. 2017;11 9:e0005959; doi: 10.1371/journal.pntd.0005959. https://www.ncbi.nlm.nih.gov/pubmed/28953892.

333. Liwanag HJ, Uy J, Bataller R, Gatchalian JR, De La Calzada B, Uy JA, et al. Soil-Transmitted Helminthiasis and Schistosomiasis in Children of Poor Families in Leyte, Philippines: Lessons for Disease Prevention and Control. J Trop Pediatr. 2017;63 5:335-45; doi: 10.1093/tropej/fmw078. https://www.ncbi.nlm.nih.gov/pubmed/28115576.

334. Li H, Wang W. Apropos: critical analysis of molluscicide application in schistosomiasis control programs in Brazil. Infect Dis Poverty. 2017;6 1:54; doi: 10.1186/s40249-017-0246-x. https://www.ncbi.nlm.nih.gov/pubmed/28270227.

335. Lee BY, Bartsch SM. How to determine if a model is right for neglected tropical disease decision making. PLoS Negl Trop Dis. 2017;11 4:e0005457; doi: 10.1371/journal.pntd.0005457. https://www.ncbi.nlm.nih.gov/pubmed/28426672.

336. Le L, Hsieh MH. Diagnosing Urogenital Schistosomiasis: Dealing with Diminishing Returns. Trends Parasitol. 2017;33 5:378-87; doi: 10.1016/j.pt.2016.12.009. https://www.ncbi.nlm.nih.gov/pubmed/28094201.

337. Lavan RP, King AI, Sutton DJ, Tunceli K. Rationale and support for a One Health program for canine vaccination as the most cost-effective means of controlling zoonotic rabies in endemic settings. Vaccine. 2017;35 13:1668-74; doi: 10.1016/j.vaccine.2017.02.014. https://www.ncbi.nlm.nih.gov/pubmed/28216188.

338. Kumari A, Singh KP, Mandal A, Paswan RK, Sinha P, Das P, et al. Intracellular zinc flux causes reactive oxygen species mediated mitochondrial dysfunction leading to cell death in Leishmania donovani. PLoS One. 2017;12 6:e0178800; doi: 10.1371/journal.pone.0178800. https://www.ncbi.nlm.nih.gov/pubmed/28586364.

339. Kulinkina AV, Kosinski KC, Plummer JD, Durant JL, Bosompem KM, Adjei MN, et al. Indicators of improved water access in the context of schistosomiasis transmission in rural Eastern Region, Ghana. Sci Total Environ. 2017;579:1745-55; doi: 10.1016/j.scitotenv.2016.11.140. https://www.ncbi.nlm.nih.gov/pubmed/27939198.

340. Kruithof R, Erard V. [Food-borne trematodiases]. Rev Med Suisse. 2017;13 578:1741-4. https://www.ncbi.nlm.nih.gov/pubmed/29022660.

341. Krentel A, Gyapong M, Mallya S, Boadu NY, Amuyunzu-Nyamongo M, Stephens M, et al. Review of the factors influencing the motivation of community drug distributors towards the control and elimination of neglected tropical diseases (NTDs). PLoS Negl Trop Dis. 2017;11 12:e0006065; doi: 10.1371/journal.pntd.0006065. https://www.ncbi.nlm.nih.gov/pubmed/29211746.

342. Knipes AK, Lemoine JF, Monestime F, Fayette CR, Direny AN, Desir L, et al. Partnering for impact: Integrated transmission assessment surveys for lymphatic filariasis, soil transmitted helminths and malaria in Haiti. PLoS Negl Trop Dis. 2017;11 2:e0005387; doi: 10.1371/journal.pntd.0005387. https://www.ncbi.nlm.nih.gov/pubmed/28207792.

343. Kisoka W, Mushi D, Meyrowitsch DW, Malecela M, Simonsen PE, Tersbol BP. Dilemmas of Community-Directed Mass Drug Administration for Lymphatic Filariasis Control: A Qualitative Study from Urban and Rural Tanzania. J Biosoc Sci. 2017;49 4:447-62; doi: 10.1017/S0021932016000365. https://www.ncbi.nlm.nih.gov/pubmed/27470198.

344. Kirigia JM, Mburugu GN. The monetary value of human lives lost due to neglected tropical diseases in Africa. Infect Dis Poverty. 2017;6 1:165; doi: 10.1186/s40249-017-0379-y. https://www.ncbi.nlm.nih.gov/pubmed/29249201.

345. Kihembo C, Masiira B, Lali WZ, Matwale GK, Matovu JKB, Kaharuza F, et al. Risk Factors for Podoconiosis: Kamwenge District, Western Uganda, September 2015. Am J Trop Med Hyg. 2017;96 6:1490-6; doi: 10.4269/ajtmh.16-0932. https://www.ncbi.nlm.nih.gov/pubmed/28719274.

346. Khowawisetsut L, Sarasombath PT, Thammapalo S, Loymek S, Korbarsa T, Nochote H, et al. Therapeutic trial of doxycyclin plus ivermectin for the treatment of Brugia malayi naturally infected cats. Vet Parasitol. 2017;245:42-7; doi: 10.1016/j.vetpar.2017.08.009. https://www.ncbi.nlm.nih.gov/pubmed/28969836.

347. Kelly-Hope L, Paulo R, Thomas B, Brito M, Unnasch TR, Molyneux D. Loa loa vectors Chrysops spp.: perspectives on research, distribution, bionomics, and implications for elimination of lymphatic filariasis and onchocerciasis. Parasit Vectors. 2017;10 1:172; doi: 10.1186/s13071-017-2103-y. https://www.ncbi.nlm.nih.gov/pubmed/28381279.

348. Kastner RJ, Sicuri E, Stone CM, Matwale G, Onapa A, Tediosi F. How much will it cost to eradicate lymphatic filariasis? An analysis of the financial and economic costs of intensified efforts against lymphatic filariasis. PLoS Negl Trop Dis. 2017;11 9:e0005934; doi: 10.1371/journal.pntd.0005934. https://www.ncbi.nlm.nih.gov/pubmed/28949987.

349. Kassegne K, Zhang T, Chen SB, Xu B, Dang ZS, Deng WP, et al. Study roadmap for high-throughput development of easy to use and affordable biomarkers as diagnostics for tropical diseases: a focus on malaria and schistosomiasis. Infect Dis Poverty. 2017;6 1:130; doi: 10.1186/s40249-017-0344-9. https://www.ncbi.nlm.nih.gov/pubmed/28965490.

350. Kamhawi S. The yin and yang of leishmaniasis control. PLoS Negl Trop Dis. 2017;11 4:e0005529; doi: 10.1371/journal.pntd.0005529. https://www.ncbi.nlm.nih.gov/pubmed/28426716.

351. Kalmobe J, Ndjonka D, Boursou D, Vildina JD, Liebau E. Phytochemical analysis and in vitro anthelmintic activity of Lophira lanceolata (Ochnaceae) on the bovine parasite Onchocerca ochengi and on drug resistant strains of the free-living nematode Caenorhabditis elegans. BMC Complement Altern Med. 2017;17 1:404; doi: 10.1186/s12906-017-1904-z. https://www.ncbi.nlm.nih.gov/pubmed/28806951.

352. Johnston KL, Cook DAN, Berry NG, David Hong W, Clare RH, Goddard M, et al. Identification and prioritization of novel anti-Wolbachia chemotypes from screening a 10,000-compound diversity library. Sci Adv. 2017;3 9:eaao1551; doi: 10.1126/sciadv.aao1551. https://www.ncbi.nlm.nih.gov/pubmed/28959730.

353. Jannin J, Solano P, Quick I, Debre P. The francophone network on neglected tropical diseases. PLoS Negl Trop Dis. 2017;11 8:e0005738; doi: 10.1371/journal.pntd.0005738. https://www.ncbi.nlm.nih.gov/pubmed/28859088.

354. Iltis AS, Matthews KRW. NTD policy priorities: Science, values, and agenda setting. PLoS Negl Trop Dis. 2017;11 5:e0005431; doi: 10.1371/journal.pntd.0005431. https://www.ncbi.nlm.nih.gov/pubmed/28545108.

355. Hotez PJ, Damania A, Barua A, Stanaway J. The first "London Declaration": The Commonwealth and its neglected tropical diseases. PLoS Negl Trop Dis. 2017;11 4:e0005321; doi: 10.1371/journal.pntd.0005321. https://www.ncbi.nlm.nih.gov/pubmed/28448487.

356. Hotez PJ, Aksoy S. Will a new 2017 global leadership commit to NTDs? PLoS Negl Trop Dis. 2017;11 3:e0005309; doi: 10.1371/journal.pntd.0005309. https://www.ncbi.nlm.nih.gov/pubmed/28333927.

357. Hotez PJ. Developing and financing neglected disease vaccines in our new era of "blue marble health" and the anthropocene epoch. Vaccine. 2017;35 40:5403-5; doi: 10.1016/j.vaccine.2017.02.002. https://www.ncbi.nlm.nih.gov/pubmed/28262333.

358. Hotez PJ. Neglected tropical diseases in the time of Dr Tedros. Trans R Soc Trop Med Hyg. 2017;111 5:189-90; doi: 10.1093/trstmh/trx040. https://www.ncbi.nlm.nih.gov/pubmed/28957469.

359. Hotez PJ. New paths for PLOS Neglected Tropical Diseases: Continuing a tradition of innovation and commitment to the poor. PLoS Negl Trop Dis. 2017;11 10:e0005862; doi: 10.1371/journal.pntd.0005862. https://www.ncbi.nlm.nih.gov/pubmed/29073134.

360. Hotez PJ. The poverty-related neglected diseases: Why basic research matters. PLoS Biol. 2017;15 11:e2004186; doi: 10.1371/journal.pbio.2004186. https://www.ncbi.nlm.nih.gov/pubmed/29121043.

361. Hotez PJ. Ten failings in global neglected tropical diseases control. PLoS Negl Trop Dis. 2017;11 12:e0005896; doi: 10.1371/journal.pntd.0005896. https://www.ncbi.nlm.nih.gov/pubmed/29267282.

362. Hotez P, Aksoy S. PLOS Neglected Tropical Diseases: Ten years of progress in neglected tropical disease control and elimination ... More or less. PLoS Negl Trop Dis. 2017;11 4:e0005355; doi: 10.1371/journal.pntd.0005355. https://www.ncbi.nlm.nih.gov/pubmed/28426662.

363. Hosein S, Blake DP, Solano-Gallego L. Insights on adaptive and innate immunity in canine leishmaniosis. Parasitology. 2017;144 1:95-115; doi: 10.1017/S003118201600055X. https://www.ncbi.nlm.nih.gov/pubmed/27094260.

364. Honigsbaum M. Between Securitisation and Neglect: Managing Ebola at the Borders of Global Health. Med Hist. 2017;61 2:270-94; doi: 10.1017/mdh.2017.6. https://www.ncbi.nlm.nih.gov/pubmed/28260567.

365. Holmes KK, Bertozzi S, Bloom BR, Jha P, Gelband H, DeMaria LM, et al. Major Infectious Diseases: Key Messages from Disease Control Priorities, Third Edition. In: rd, Holmes KK, Bertozzi S, Bloom BR, Jha P, editors. Major Infectious Diseases. Washington (DC); 2017.

366. Hessler MJ, Cyrs A, Krenzke SC, Mahmoud ES, Sikasunge C, Mwansa J, et al. Detection of duo-schistosome infection from filtered urine samples from school children in Zambia after MDA. PLoS One. 2017;12 12:e0189400; doi: 10.1371/journal.pone.0189400. https://www.ncbi.nlm.nih.gov/pubmed/29228024.

367. Herricks JR, Hotez PJ, Wanga V, Coffeng LE, Haagsma JA, Basanez MG, et al. The global burden of disease study 2013: What does it mean for the NTDs? PLoS Negl Trop Dis. 2017;11 8:e0005424; doi: 10.1371/journal.pntd.0005424. https://www.ncbi.nlm.nih.gov/pubmed/28771480.

368. He P, Wang W, Sanogo B, Zeng X, Sun X, Lv Z, et al. Molluscicidal activity and mechanism of toxicity of a novel salicylanilide ester derivative against Biomphalaria species. Parasit Vectors. 2017;10 1:383; doi: 10.1186/s13071-017-2313-3. https://www.ncbi.nlm.nih.gov/pubmed/28793917.

369. Hardy M, Engelman D, Steer A. Scabies: A clinical update. Aust Fam Physician. 2017;46 5:264-8. https://www.ncbi.nlm.nih.gov/pubmed/28472570.

370. Gyawali N, Taylor-Robinson AW. Confronting the Emerging Threat to Public Health in Northern Australia of Neglected Indigenous Arboviruses. Trop Med Infect Dis. 2017;2 4; doi: 10.3390/tropicalmed2040055. https://www.ncbi.nlm.nih.gov/pubmed/30270912.

371. Gripper LB, Welburn SC. Neurocysticercosis infection and disease-A review. Acta Trop. 2017;166:218-24; doi: 10.1016/j.actatropica.2016.11.015. https://www.ncbi.nlm.nih.gov/pubmed/27880878.

372. Gower CM, Gehre F, Marques SR, Lamberton PHL, Lwambo NJ, Webster JP. Phenotypic and genotypic monitoring of Schistosoma mansoni in Tanzanian schoolchildren five years into a preventative chemotherapy national control programme. Parasit Vectors. 2017;10 1:593; doi: 10.1186/s13071-017-2533-6. https://www.ncbi.nlm.nih.gov/pubmed/29197426.

373. Globisch D, Eubanks LM, Shirey RJ, Pfarr KM, Wanji S, Debrah AY, et al. Validation of onchocerciasis biomarker N-acetyltyramine-O-glucuronide (NATOG). Bioorg Med Chem Lett. 2017;27 15:3436-40; doi: 10.1016/j.bmcl.2017.05.082. https://www.ncbi.nlm.nih.gov/pubmed/28600214.

374. Gasser RB, Tan P, Teh BT, Wongkham S, Young ND. Genomics of worms, with an emphasis on Opisthorchis viverrini - opportunities for fundamental discovery and biomedical outcomes. Parasitol Int. 2017;66 4:341-5; doi: 10.1016/j.parint.2016.01.005. https://www.ncbi.nlm.nih.gov/pubmed/26792076.

375. Garchitorena A, Sokolow SH, Roche B, Ngonghala CN, Jocque M, Lund A, et al. Disease ecology, health and the environment: a framework to account for ecological and socio-economic drivers in the control of neglected tropical diseases. Philos Trans R Soc Lond B Biol Sci. 2017;372 1722; doi: 10.1098/rstb.2016.0128. https://www.ncbi.nlm.nih.gov/pubmed/28438917.

376. Gao D, Lietman TM, Dong CP, Porco TC. Mass drug administration: the importance of synchrony. Math Med Biol. 2017;34 2:241-60; doi: 10.1093/imammb/dqw005. https://www.ncbi.nlm.nih.gov/pubmed/27118395.

377. Furst T, Salari P, Llamas LM, Steinmann P, Fitzpatrick C, Tediosi F. Global health policy and neglected tropical diseases: Then, now, and in the years to come. PLoS Negl Trop Dis. 2017;11 9:e0005759; doi: 10.1371/journal.pntd.0005759. https://www.ncbi.nlm.nih.gov/pubmed/28910281.

378. Fung IC, Jackson AM, Ahweyevu JO, Grizzle JH, Yin J, Tse ZTH, et al. #Globalhealth Twitter Conversations on #Malaria, #HIV, #TB, #NCDS, and #NTDS: a Cross-Sectional Analysis. Ann Glob Health. 2017;83 3-4:682-90; doi: 10.1016/j.aogh.2017.09.006. https://www.ncbi.nlm.nih.gov/pubmed/29221545.

379. Franco Marcelino PR, da Silva VL, Rodrigues Philippini R, Von Zuben CJ, Contiero J, Dos Santos JC, et al. Biosurfactants produced by Scheffersomyces stipitis cultured in sugarcane bagasse hydrolysate as new green larvicides for the control of Aedes aegypti, a vector of neglected tropical diseases. PLoS One. 2017;12 11:e0187125; doi: 10.1371/journal.pone.0187125. https://www.ncbi.nlm.nih.gov/pubmed/29125845.

380. Fooks AR, Cliquet F, Finke S, Freuling C, Hemachudha T, Mani RS, et al. Rabies. Nat Rev Dis Primers. 2017;3:17091; doi: 10.1038/nrdp.2017.91. https://www.ncbi.nlm.nih.gov/pubmed/29188797.

381. Fitzpatrick C, Sankara DP, Agua JF, Jonnalagedda L, Rumi F, Weiss A, et al. The cost-effectiveness of an eradication programme in the end game: Evidence from guinea worm disease. PLoS Negl Trop Dis. 2017;11 10:e0005922; doi: 10.1371/journal.pntd.0005922. https://www.ncbi.nlm.nih.gov/pubmed/28981510.

382. Fitzpatrick C, Nwankwo U, Lenk E, de Vlas SJ, Bundy DAP. An Investment Case for Ending Neglected Tropical Diseases. In: rd, Holmes KK, Bertozzi S, Bloom BR, Jha P, editors. Major Infectious Diseases. Washington (DC); 2017.

383. Fitzpatrick C, Haines A, Bangert M, Farlow A, Hemingway J, Velayudhan R. An economic evaluation of vector control in the age of a dengue vaccine. PLoS Negl Trop Dis. 2017;11 8:e0005785; doi: 10.1371/journal.pntd.0005785. https://www.ncbi.nlm.nih.gov/pubmed/28806786.

384. Fernandes CFC, Pereira SDS, Luiz MB, Zuliani JP, Furtado GP, Stabeli RG. Camelid Single-Domain Antibodies As an Alternative to Overcome Challenges Related to the Prevention, Detection, and Control of Neglected Tropical Diseases. Front Immunol. 2017;8:653; doi: 10.3389/fimmu.2017.00653. https://www.ncbi.nlm.nih.gov/pubmed/28649245.

385. Faria AR, Pires SDF, Reis AB, Coura-Vital W, Silveira J, Sousa GM, et al. Canine visceral leishmaniasis follow-up: a new anti-IgG serological test more sensitive than ITS-1 conventional PCR. Vet Parasitol. 2017;248:62-7; doi: 10.1016/j.vetpar.2017.10.020. https://www.ncbi.nlm.nih.gov/pubmed/29173543.

386. Fahrion AS, Taylor LH, Torres G, Muller T, Durr S, Knopf L, et al. The Road to Dog Rabies Control and Elimination-What Keeps Us from Moving Faster? Front Public Health. 2017;5:103; doi: 10.3389/fpubh.2017.00103. https://www.ncbi.nlm.nih.gov/pubmed/28555183.

387. Engels D. Neglected tropical diseases: A proxy for equitable development and shared prosperity. PLoS Negl Trop Dis. 2017;11 4:e0005419; doi: 10.1371/journal.pntd.0005419. https://www.ncbi.nlm.nih.gov/pubmed/28426668.

388. Ekanem EE, Akapan FM, Eyong ME. Urinary schistosomiasis in school children of a southern nigerian community 8 years after the provision of potable water. Niger Postgrad Med J. 2017;24 4:201-4; doi: 10.4103/npmj.npmj_136_17. https://www.ncbi.nlm.nih.gov/pubmed/29355157.

389. Eisenstein M. Partnering to promote research where it matters. PLoS Negl Trop Dis. 2017;11 4:e0005530; doi: 10.1371/journal.pntd.0005530. https://www.ncbi.nlm.nih.gov/pubmed/28426750.

390. Dyson L, Stolk WA, Farrell SH, Hollingsworth TD. Measuring and modelling the effects of systematic non-adherence to mass drug administration. Epidemics. 2017;18:56-66; doi: 10.1016/j.epidem.2017.02.002. https://www.ncbi.nlm.nih.gov/pubmed/28279457.

391. Dybing NA, Jacobson C, Irwin P, Algar D, Adams PJ. Leptospira Species in Feral Cats and Black Rats from Western Australia and Christmas Island. Vector Borne Zoonotic Dis. 2017;17 5:319-24; doi: 10.1089/vbz.2016.1992. https://www.ncbi.nlm.nih.gov/pubmed/28437186.

392. Dunn JC, Bettis AA, Wyine NY, Lwin AMM, Lwin ST, Su KK, et al. A cross-sectional survey of soil-transmitted helminthiases in two Myanmar villages receiving mass drug administration: epidemiology of infection with a focus on adults. Parasit Vectors. 2017;10 1:374; doi: 10.1186/s13071-017-2306-2. https://www.ncbi.nlm.nih.gov/pubmed/28778217.

393. Dorny P, Dermauw V, Van Hul A, Trevisan C, Gabriel S. Serological diagnosis of Taenia solium in pigs: No measurable circulating antigens and antibody response following exposure to Taenia saginata oncospheres. Vet Parasitol. 2017;245:39-41; doi: 10.1016/j.vetpar.2017.08.008. https://www.ncbi.nlm.nih.gov/pubmed/28969835.

394. Dichiara M, Marrazzo A, Prezzavento O, Collina S, Rescifina A, Amata E. Repurposing of Human Kinase Inhibitors in Neglected Protozoan Diseases. ChemMedChem. 2017;12 16:1235-53; doi: 10.1002/cmdc.201700259. https://www.ncbi.nlm.nih.gov/pubmed/28590590.

395. Dhawan R, Kumar M, Mohanty AK, Dey G, Advani J, Prasad TS, et al. Mosquito-Borne Diseases and Omics: Salivary Gland Proteome of the Female Aedes aegypti Mosquito. OMICS. 2017;21 1:45-54; doi: 10.1089/omi.2016.0160. https://www.ncbi.nlm.nih.gov/pubmed/28271980.

396. Deribe K, Kebede B, Mengistu B, Negussie H, Sileshi M, Tamiru M, et al. Podoconiosis in Ethiopia: From Neglect to Priority Public Health Problem. Ethiop Med J. 2017;55 Suppl 1:65-74. https://www.ncbi.nlm.nih.gov/pubmed/28878431.

397. DebRoy S, Prosper O, Mishoe A, Mubayi A. Challenges in modeling complexity of neglected tropical diseases: a review of dynamics of visceral leishmaniasis in resource limited settings. Emerg Themes Epidemiol. 2017;14:10; doi: 10.1186/s12982-017-0065-3. https://www.ncbi.nlm.nih.gov/pubmed/28936226.

398. de Wit LA, Croll DA, Tershy B, Newton KM, Spatz DR, Holmes ND, et al. Estimating Burdens of Neglected Tropical Zoonotic Diseases on Islands with Introduced Mammals. Am J Trop Med Hyg. 2017;96 3:749-57; doi: 10.4269/ajtmh.16-0573. https://www.ncbi.nlm.nih.gov/pubmed/28138052.

399. Croke K, Hicks JH, Hsu E, Kremer M, Miguel E. Should the WHO withdraw support for mass deworming? PLoS Negl Trop Dis. 2017;11 6:e0005481; doi: 10.1371/journal.pntd.0005481. https://www.ncbi.nlm.nih.gov/pubmed/28594928.

400. Cortes A, Sotillo J, Munoz-Antoli C, Molina-Duran J, Esteban JG, Toledo R. Antibody trapping: A novel mechanism of parasite immune evasion by the trematode Echinostoma caproni. PLoS Negl Trop Dis. 2017;11 7:e0005773; doi: 10.1371/journal.pntd.0005773. https://www.ncbi.nlm.nih.gov/pubmed/28715423.

401. Conrad NL, Cruz McBride FW, Souza JD, Silveira MM, Felix S, Mendonca KS, et al. LigB subunit vaccine confers sterile immunity against challenge in the hamster model of leptospirosis. PLoS Negl Trop Dis. 2017;11 3:e0005441; doi: 10.1371/journal.pntd.0005441. https://www.ncbi.nlm.nih.gov/pubmed/28301479.

402. Coelho CH, Durigan M, Leal DAG, Schneider AB, Franco RMB, Singer SM. Giardiasis as a neglected disease in Brazil: Systematic review of 20 years of publications. PLoS Negl Trop Dis. 2017;11 10:e0006005; doi: 10.1371/journal.pntd.0006005. https://www.ncbi.nlm.nih.gov/pubmed/29065126.

403. Cleaveland S, Hampson K. Rabies elimination research: juxtaposing optimism, pragmatism and realism. Proc Biol Sci. 2017;284 1869; doi: 10.1098/rspb.2017.1880. https://www.ncbi.nlm.nih.gov/pubmed/29263285.

404. Ciddio M, Mari L, Sokolow SH, De Leo GA, Casagrandi R, Gatto M. The spatial spread of schistosomiasis: A multidimensional network model applied to Saint-Louis region, Senegal. Adv Water Resour. 2017;108:406-15; doi: 10.1016/j.advwatres.2016.10.012. https://www.ncbi.nlm.nih.gov/pubmed/29056816.

405. Chesnais CB, Takougang I, Paguele M, Pion SD, Boussinesq M. Excess mortality associated with loiasis: a retrospective population-based cohort study. Lancet Infect Dis. 2017;17 1:108-16; doi: 10.1016/S1473-3099(16)30405-4. https://www.ncbi.nlm.nih.gov/pubmed/27777031.

406. Chen T, Jiang H, Sun H, Xie Z, Ren P, Zhao L, et al. Sequence analysis and characterization of pyruvate kinase from Clonorchis sinensis, a 53.1-kDa homopentamer, implicated immune protective efficacy against clonorchiasis. Parasit Vectors. 2017;10 1:557; doi: 10.1186/s13071-017-2494-9. https://www.ncbi.nlm.nih.gov/pubmed/29121987.

407. Chattu VK. The rise of global health diplomacy: An interdisciplinary concept linking health and international relations. Indian J Public Health. 2017;61 2:134-6; doi: 10.4103/ijph.IJPH_67_16. https://www.ncbi.nlm.nih.gov/pubmed/28721965.

408. Cha S, Hong ST, Lee YH, Lee KH, Cho DS, Lee J, et al. Nationwide cross-sectional survey of schistosomiasis and soil-transmitted helminthiasis in Sudan: study protocol. BMC Public Health. 2017;17 1:703; doi: 10.1186/s12889-017-4719-4. https://www.ncbi.nlm.nih.gov/pubmed/28899362.

409. Carrizosa Moog J, Kakooza-Mwesige A, Tan CT. Epilepsy in the tropics: Emerging etiologies. Seizure. 2017;44:108-12; doi: 10.1016/j.seizure.2016.11.032. https://www.ncbi.nlm.nih.gov/pubmed/27986419.

410. Brelsford JB, Plieskatt JL, Yakovleva A, Jariwala A, Keegan BP, Peng J, et al. Advances in neglected tropical disease vaccines: Developing relative potency and functional assays for the Na-GST-1/Alhydrogel hookworm vaccine. PLoS Negl Trop Dis. 2017;11 2:e0005385; doi: 10.1371/journal.pntd.0005385. https://www.ncbi.nlm.nih.gov/pubmed/28192438.

411. Bonell A, Lubell Y, Newton PN, Crump JA, Paris DH. Estimating the burden of scrub typhus: A systematic review. PLoS Negl Trop Dis. 2017;11 9:e0005838; doi: 10.1371/journal.pntd.0005838. https://www.ncbi.nlm.nih.gov/pubmed/28945755.

412. Beyene HB, Bekele A, Shifara A, Ebstie YA, Desalegn Z, Kebede Z, et al. Elimination of Guinea Worm Disease in Ethiopia; Current Status of the Disease's, Eradication Strategies and Challenges to the End Game. Ethiop Med J. 2017;55 Suppl 1:15-31. https://www.ncbi.nlm.nih.gov/pubmed/28878428.

413. Bergquist R, Zhou XN, Rollinson D, Reinhard-Rupp J, Klohe K. Elimination of schistosomiasis: the tools required. Infect Dis Poverty. 2017;6 1:158; doi: 10.1186/s40249-017-0370-7. https://www.ncbi.nlm.nih.gov/pubmed/29151362.

414. Bello Corassa R, Aceijas C, Alves PAB, Garelick H. Evolution of Chagas' disease in Brazil. Epidemiological perspective and challenges for the future: a critical review. Perspect Public Health. 2017;137 5:289-95; doi: 10.1177/1757913916671160. https://www.ncbi.nlm.nih.gov/pubmed/27758973.

415. Bangert M, Molyneux DH, Lindsay SW, Fitzpatrick C, Engels D. The cross-cutting contribution of the end of neglected tropical diseases to the sustainable development goals. Infect Dis Poverty. 2017;6 1:73; doi: 10.1186/s40249-017-0288-0. https://www.ncbi.nlm.nih.gov/pubmed/28372566.

416. Bandyopadhyay D, Samano S, Villalobos-Rocha JC, Sanchez-Torres LE, Nogueda-Torres B, Rivera G, et al. A Practical Green Synthesis and Biological Evaluation of Benzimidazoles Against Two Neglected Tropical Diseases: Chagas and Leishmaniasis. Curr Med Chem. 2017;24 41:4714-25; doi: 10.2174/0929867325666171201101807. https://www.ncbi.nlm.nih.gov/pubmed/23317160.

417. Balakrishnan VS. Ending neglected tropical diseases. Lancet Infect Dis. 2017;17 6:584-5; doi: 10.1016/S1473-3099(17)30253-0. https://www.ncbi.nlm.nih.gov/pubmed/28555584.

418. Bakir H, Hadi M, Jurdi M. Towards a renewed public health regulatory and surveillance role in water, sanitation and hygiene (Editorial). East Mediterr Health J. 2017;23 8:525-6. https://www.ncbi.nlm.nih.gov/pubmed/30378670.

419. Ash A, Okello A, Khamlome B, Inthavong P, Allen J, Thompson RCA. Controlling Taenia solium and soil transmitted helminths in a northern Lao PDR village: Impact of a triple dose albendazole regime. Acta Trop. 2017;174:171-8; doi: 10.1016/j.actatropica.2015.05.018. https://www.ncbi.nlm.nih.gov/pubmed/26001973.

420. Amazigo U, Crump A, Godal T. Recognising the role of community-directed treatment and of women in the fight against NTDs. Lancet Glob Health. 2017;5 6:e569-e70; doi: 10.1016/S2214-109X(17)30171-7. https://www.ncbi.nlm.nih.gov/pubmed/28433564.

421. Alpern JD, Lopez-Velez R, Stauffer WM. Access to benznidazole for Chagas disease in the United States-Cautious optimism? PLoS Negl Trop Dis. 2017;11 9:e0005794; doi: 10.1371/journal.pntd.0005794. https://www.ncbi.nlm.nih.gov/pubmed/28910299.

422. Allan F, Sousa-Figueiredo JC, Emery AM, Paulo R, Mirante C, Sebastiao A, et al. Mapping freshwater snails in north-western Angola: distribution, identity and molecular diversity of medically important taxa. Parasit Vectors. 2017;10 1:460; doi: 10.1186/s13071-017-2395-y. https://www.ncbi.nlm.nih.gov/pubmed/29017583.

423. Allan ER, Tennessen JA, Bollmann SR, Hanington PC, Bayne CJ, Blouin MS. Schistosome infectivity in the snail, Biomphalaria glabrata, is partially dependent on the expression of Grctm6, a Guadeloupe Resistance Complex protein. PLoS Negl Trop Dis. 2017;11 2:e0005362; doi: 10.1371/journal.pntd.0005362. https://www.ncbi.nlm.nih.gov/pubmed/28158185.

424. Akinsolu FT, de Paiva VN, Souza SS, Varga O. Patent landscape of neglected tropical diseases: an analysis of worldwide patent families. Global Health. 2017;13 1:82; doi: 10.1186/s12992-017-0306-9. https://www.ncbi.nlm.nih.gov/pubmed/29137663.

425. Aerts C, Sunyoto T, Tediosi F, Sicuri E. Are public-private partnerships the solution to tackle neglected tropical diseases? A systematic review of the literature. Health Policy. 2017;121 7:745-54; doi: 10.1016/j.healthpol.2017.05.005. https://www.ncbi.nlm.nih.gov/pubmed/28579276.

426. Acup C, Bardosh KL, Picozzi K, Waiswa C, Welburn SC. Factors influencing passive surveillance for T. b. rhodesiense human african trypanosomiasis in Uganda. Acta Trop. 2017;165:230-9; doi: 10.1016/j.actatropica.2016.05.009. https://www.ncbi.nlm.nih.gov/pubmed/27212706.

427. Ensuring the timely supply and management of medicines for preventive chemotherapy against neglected tropical diseases. Wkly Epidemiol Rec. 2017;92 13:155-64. https://www.ncbi.nlm.nih.gov/pubmed/28361529.

428. Zijlstra EE. Visceral leishmaniasis: a forgotten epidemic. Arch Dis Child. 2016;101 6:561-7; doi: 10.1136/archdischild-2015-309302. https://www.ncbi.nlm.nih.gov/pubmed/26895806.

429. Zhang SQ, Sun CS, Wang M, Lin DD, Zhou XN, Wang TP. Epidemiological Features and Effectiveness of Schistosomiasis Control Programme in Lake and Marshland Region in The People's Republic of China. Adv Parasitol. 2016;92:39-71; doi: 10.1016/bs.apar.2016.02.018. https://www.ncbi.nlm.nih.gov/pubmed/27137442.

430. Zamanian M, Andersen EC. Prospects and challenges of CRISPR/Cas genome editing for the study and control of neglected vector-borne nematode diseases. FEBS J. 2016;283 17:3204-21; doi: 10.1111/febs.13781. https://www.ncbi.nlm.nih.gov/pubmed/27300487.

431. Yin M, Li H, Blair D, Xu B, Feng Z, Hu W. Temporal genetic diversity of Schistosoma japonicum in two endemic sites in China revealed by microsatellite markers. Parasit Vectors. 2016;9:36; doi: 10.1186/s13071-016-1326-7. https://www.ncbi.nlm.nih.gov/pubmed/26800884.

432. Yamada H, Hirabayashi F, Brunger C. [International Partnership for Therapeutic Drug Development of NTDs by DNDi]. Yakugaku Zasshi. 2016;136 2:213-22; doi: 10.1248/yakushi.15-00233-2. https://www.ncbi.nlm.nih.gov/pubmed/26831796.

433. Woolley RJ, Velink A, Phillips RO, Thompson WA, Abass KM, van der Werf TS, et al. Experiences of Pain and Expectations for Its Treatment Among Former Buruli Ulcer Patients. Am J Trop Med Hyg. 2016;95 5:1011-5; doi: 10.4269/ajtmh.16-0419. https://www.ncbi.nlm.nih.gov/pubmed/27621302.

434. Welburn SC, Bardosh KL, Coleman PG. Novel Financing Model for Neglected Tropical Diseases: Development Impact Bonds Applied to Sleeping Sickness and Rabies Control. PLoS Negl Trop Dis. 2016;10 11:e0005000; doi: 10.1371/journal.pntd.0005000. https://www.ncbi.nlm.nih.gov/pubmed/27855156.

435. Webster JP, Gower CM, Knowles SC, Molyneux DH, Fenton A. One health - an ecological and evolutionary framework for tackling Neglected Zoonotic Diseases. Evol Appl. 2016;9 2:313-33; doi: 10.1111/eva.12341. https://www.ncbi.nlm.nih.gov/pubmed/26834828.

436. Wanji S, Tayong DB, Layland LE, Datchoua Poutcheu FR, Ndongmo WP, Kengne-Ouafo JA, et al. Update on the distribution of Mansonella perstans in the southern part of Cameroon: influence of ecological factors and mass drug administration with ivermectin. Parasit Vectors. 2016;9 1:311; doi: 10.1186/s13071-016-1595-1. https://www.ncbi.nlm.nih.gov/pubmed/27245442.

437. Wangchuk P, Giacomin PR, Pearson MS, Smout MJ, Loukas A. Identification of lead chemotherapeutic agents from medicinal plants against blood flukes and whipworms. Sci Rep. 2016;6:32101; doi: 10.1038/srep32101. https://www.ncbi.nlm.nih.gov/pubmed/27572696.

438. Waite RC, Velleman Y, Woods G, Chitty A, Freeman MC. Integration of water, sanitation and hygiene for the control of neglected tropical diseases: a review of progress and the way forward. Int Health. 2016;8 Suppl 1:i22-7; doi: 10.1093/inthealth/ihw003. https://www.ncbi.nlm.nih.gov/pubmed/26940306.

439. Villanueva Forero M, Soria Moncada J, Cornejo Leon M, Soto Arquinigo L, Arauco Brown R. An Unusual Case of Anaphylaxis after Blunt Abdominal Trauma. J Emerg Med. 2016;50 3:e143-6; doi: 10.1016/j.jemermed.2015.12.001. https://www.ncbi.nlm.nih.gov/pubmed/26818384.

440. Turner HC, Truscott JE, Fleming FM, Hollingsworth TD, Brooker SJ, Anderson RM. Cost-effectiveness of scaling up mass drug administration for the control of soil-transmitted helminths: a comparison of cost function and constant costs analyses. Lancet Infect Dis. 2016;16 7:838-46; doi: 10.1016/S1473-3099(15)00268-6. https://www.ncbi.nlm.nih.gov/pubmed/26897109.

441. Turner HC, Bettis AA, Chu BK, McFarland DA, Hooper PJ, Ottesen EA, et al. The health and economic benefits of the global programme to eliminate lymphatic filariasis (2000-2014). Infect Dis Poverty. 2016;5 1:54; doi: 10.1186/s40249-016-0147-4. https://www.ncbi.nlm.nih.gov/pubmed/27388873.

442. Truscott JE, Turner HC, Farrell SH, Anderson RM. Soil-Transmitted Helminths: Mathematical Models of Transmission, the Impact of Mass Drug Administration and Transmission Elimination Criteria. Adv Parasitol. 2016;94:133-98; doi: 10.1016/bs.apar.2016.08.002. https://www.ncbi.nlm.nih.gov/pubmed/27756454.

443. Trainor-Moss S, Mutapi F. Schistosomiasis therapeutics: whats in the pipeline? Expert Rev Clin Pharmacol. 2016;9 2:157-60; doi: 10.1586/17512433.2015.1102051. https://www.ncbi.nlm.nih.gov/pubmed/26508363.

444. Tora A, Ayode D, Tadele G, Farrell D, Davey G, McBride CM. Interpretations of education about gene-environment influences on health in rural Ethiopia: the context of a neglected tropical disease. Int Health. 2016;8 4:253-60; doi: 10.1093/inthealth/ihw016. https://www.ncbi.nlm.nih.gov/pubmed/27114426.

445. Toledo CE, Jacobson J, Wainwright EC, Ottesen EA, Lammie PJ. RRR for NNN-a rapid research response for the Neglected Tropical Disease NGDO Network: a novel framework to challenges faced by the global programs targeting neglected tropical diseases. Int Health. 2016;8 Suppl 1:i12-4; doi: 10.1093/inthealth/ihv072. https://www.ncbi.nlm.nih.gov/pubmed/26940303.

446. Tambo E, Ugwu CE, Guan Y, Wei D, Xiao N, Xiao-Nong Z. China-Africa Health Development Initiatives: Benefits and Implications for Shaping Innovative and Evidence-informed National Health Policies and Programs in Sub-saharan African Countries. Int J MCH AIDS. 2016;5 2:119-33; doi: 10.21106/ijma.100. https://www.ncbi.nlm.nih.gov/pubmed/28058199.

447. Suroowan S, Mahomoodally F, Ragoo L. Management and Treatment of Dengue and Chikungunya - Natural Products to the Rescue. Comb Chem High Throughput Screen. 2016;19 7:554-64; doi: 10.2174/1386207319666160506123401. https://www.ncbi.nlm.nih.gov/pubmed/27151484.

448. Stolk WA, Kulik MC, le Rutte EA, Jacobson J, Richardus JH, de Vlas SJ, et al. Between-Country Inequalities in the Neglected Tropical Disease Burden in 1990 and 2010, with Projections for 2020. PLoS Negl Trop Dis. 2016;10 5:e0004560; doi: 10.1371/journal.pntd.0004560. https://www.ncbi.nlm.nih.gov/pubmed/27171193.

449. Stanton M, Molineux A, Mackenzie C, Kelly-Hope L. Mobile Technology for Empowering Health Workers in Underserved Communities: New Approaches to Facilitate the Elimination of Neglected Tropical Diseases. JMIR Public Health Surveill. 2016;2 1:e2; doi: 10.2196/publichealth.5064. https://www.ncbi.nlm.nih.gov/pubmed/27227155.

450. Staff PNTD. Correction: Buruli Ulcer in Cameroon: The Development and Impact of the National Control Programme. PLoS Negl Trop Dis. 2016;10 2:e0004438; doi: 10.1371/journal.pntd.0004438. https://www.ncbi.nlm.nih.gov/pubmed/26828205.

451. Srivastava S, Shankar P, Mishra J, Singh S. Possibilities and challenges for developing a successful vaccine for leishmaniasis. Parasit Vectors. 2016;9 1:277; doi: 10.1186/s13071-016-1553-y. https://www.ncbi.nlm.nih.gov/pubmed/27175732.

452. Soriano-Arandes A, Angheben A, Serre-Delcor N, Trevino-Maruri B, Gomez IPJ, Jackson Y. Control and management of congenital Chagas disease in Europe and other non-endemic countries: current policies and practices. Trop Med Int Health. 2016;21 5:590-6; doi: 10.1111/tmi.12687. https://www.ncbi.nlm.nih.gov/pubmed/26932338.

453. Smith J, Taylor EM. What Is Next for NTDs in the Era of the Sustainable Development Goals? PLoS Negl Trop Dis. 2016;10 7:e0004719; doi: 10.1371/journal.pntd.0004719. https://www.ncbi.nlm.nih.gov/pubmed/27387209.

454. Singh K, Muddasiru D, Singh J. Current status of schistosomiasis in Sokoto, Nigeria. Parasite Epidemiol Control. 2016;1 3:239-44; doi: 10.1016/j.parepi.2016.08.003. https://www.ncbi.nlm.nih.gov/pubmed/29988177.

455. Shuford KV, Turner HC, Anderson RM. Compliance with anthelmintic treatment in the neglected tropical diseases control programmes: a systematic review. Parasit Vectors. 2016;9:29; doi: 10.1186/s13071-016-1311-1. https://www.ncbi.nlm.nih.gov/pubmed/26813098.

456. Shiraho EA, Eric AL, Mwangi IN, Maina GM, Kinuthia JM, Mutuku MW, et al. Development of a Loop Mediated Isothermal Amplification for Diagnosis of Ascaris lumbricoides in Fecal Samples. J Parasitol Res. 2016;2016:7376207; doi: 10.1155/2016/7376207. https://www.ncbi.nlm.nih.gov/pubmed/27882242.

457. Samy AM, Annajar BB, Dokhan MR, Boussaa S, Peterson AT. Coarse-resolution Ecology of Etiological Agent, Vector, and Reservoirs of Zoonotic Cutaneous Leishmaniasis in Libya. PLoS Negl Trop Dis. 2016;10 2:e0004381; doi: 10.1371/journal.pntd.0004381. https://www.ncbi.nlm.nih.gov/pubmed/26863317.

458. Rotondo LA, Harrison W, Bush S, Hopkins AD, Koporc K. The Neglected Tropical Disease Non-governmental Development Organization Network (NNN): the value and future of a global network aiming to control and eliminate NTDs. Int Health. 2016;8 Suppl 1:i4-6; doi: 10.1093/inthealth/ihw004. https://www.ncbi.nlm.nih.gov/pubmed/26940309.

459. Rosenberg M, Utzinger J, Addiss DG. Preventive Chemotherapy Versus Innovative and Intensified Disease Management in Neglected Tropical Diseases: A Distinction Whose Shelf Life Has Expired. PLoS Negl Trop Dis. 2016;10 4:e0004521; doi: 10.1371/journal.pntd.0004521. https://www.ncbi.nlm.nih.gov/pubmed/27077916.

460. Roiko MS, Schmitt BH, Relich RF, Meyer TL, Zhang S, Davis TE. An unusual presentation of leishmaniasis in a human immunodeficiency virus-positive individual. JMM Case Rep. 2016;3 1:e005011; doi: 10.1099/jmmcr.0.005011. https://www.ncbi.nlm.nih.gov/pubmed/28348746.

461. Rock KS, Quinnell RJ, Medley GF, Courtenay O. Progress in the Mathematical Modelling of Visceral Leishmaniasis. Adv Parasitol. 2016;94:49-131; doi: 10.1016/bs.apar.2016.08.001. https://www.ncbi.nlm.nih.gov/pubmed/27756459.

462. Ridge A. Diary of a parliamentary intern. Vet Rec. 2016;178 9:ii; doi: 10.1136/vr.i1093. https://www.ncbi.nlm.nih.gov/pubmed/26917852.

463. Rey JL, Milleliri JM. Behind neglected tropical deseases are... neglected people with diseases. Med Sante Trop. 2016;26 2:116-7; doi: 10.1684/mst.2016.0576. https://www.ncbi.nlm.nih.gov/pubmed/27412969.

464. Pohlig G, Bernhard SC, Blum J, Burri C, Mpanya A, Lubaki JP, et al. Efficacy and Safety of Pafuramidine versus Pentamidine Maleate for Treatment of First Stage Sleeping Sickness in a Randomized, Comparator-Controlled, International Phase 3 Clinical Trial. PLoS Negl Trop Dis. 2016;10 2:e0004363; doi: 10.1371/journal.pntd.0004363. https://www.ncbi.nlm.nih.gov/pubmed/26882015.

465. Parker M, Polman K, Allen T. Neglected Tropical Diseases in Biosocial Perspective. J Biosoc Sci. 2016;48 Suppl 1:S1-S15; doi: 10.1017/S0021932016000274. https://www.ncbi.nlm.nih.gov/pubmed/27428062.

466. Overgaard HJ, Alexander N, Matiz MI, Jaramillo JF, Olano VA, Vargas S, et al. A Cluster-Randomized Controlled Trial to Reduce Diarrheal Disease and Dengue Entomological Risk Factors in Rural Primary Schools in Colombia. PLoS Negl Trop Dis. 2016;10 11:e0005106; doi: 10.1371/journal.pntd.0005106. https://www.ncbi.nlm.nih.gov/pubmed/27820821.

467. Ong KI, Araki H, Kano S, Jimba M. The potential of positive deviance approach for the sustainable control of neglected tropical diseases. Trop Med Health. 2016;44:20; doi: 10.1186/s41182-016-0023-8. https://www.ncbi.nlm.nih.gov/pubmed/27436959.

468. Okello AL, Thomas L, Inthavong P, Ash A, Khamlome B, Keokamphet C, et al. Assessing the impact of a joint human-porcine intervention package for Taenia solium control: Results of a pilot study from northern Lao PDR. Acta Trop. 2016;159:185-91; doi: 10.1016/j.actatropica.2016.03.012. https://www.ncbi.nlm.nih.gov/pubmed/26992295.

469. Odhiambo GO, Musuva RM, Odiere MR, Mwinzi PN. Experiences and perspectives of community health workers from implementing treatment for schistosomiasis using the community directed intervention strategy in an informal settlement in Kisumu City, western Kenya. BMC Public Health. 2016;16:986; doi: 10.1186/s12889-016-3662-0. https://www.ncbi.nlm.nih.gov/pubmed/27634152.

470. Norman FF, Fanciulli C, Perez-Molina JA, Monge-Maillo B, Lopez-Velez R. Imported and autochthonous leprosy presenting in Madrid (1989-2015): A case series and review of the literature. Travel Med Infect Dis. 2016;14 4:331-49; doi: 10.1016/j.tmaid.2016.06.008. https://www.ncbi.nlm.nih.gov/pubmed/27393660.

471. Nguyen T, Cheong FW, Liew JW, Lau YL. Seroprevalence of fascioliasis, toxocariasis, strongyloidiasis and cysticercosis in blood samples diagnosed in Medic Medical Center Laboratory, Ho Chi Minh City, Vietnam in 2012. Parasit Vectors. 2016;9 1:486; doi: 10.1186/s13071-016-1780-2. https://www.ncbi.nlm.nih.gov/pubmed/27595647.

472. Netto MJ, Bonfim C, Brandao E, Aguiar-Santos AM, Medeiros Z. Burden of lymphatic filariasis morbidity in an area of low endemicity in Brazil. Acta Trop. 2016;163:54-60; doi: 10.1016/j.actatropica.2016.07.006. https://www.ncbi.nlm.nih.gov/pubmed/27427218.

473. Namatame I. [Activity of NTDs Drug-discovery Research Consortium]. Yakugaku Zasshi. 2016;136 2:231-6; doi: 10.1248/yakushi.15-00233-4. https://www.ncbi.nlm.nih.gov/pubmed/26831798.

474. Mwingira UJ, Means AR, Chikawe M, Kilembe B, Lyimo D, Crowley K, et al. Integrating Neglected Tropical Disease and Immunization Programs: The Experiences of the Tanzanian Ministry of Health. Am J Trop Med Hyg. 2016;95 3:505-7; doi: 10.4269/ajtmh.15-0724. https://www.ncbi.nlm.nih.gov/pubmed/27246449.

475. Muhammad N, Mpyet C, Adamu MD, William A, Umar MM, Goyol M, et al. Mapping Trachoma in Kaduna State, Nigeria: Results of 23 Local Government Area-Level, Population-Based Prevalence Surveys. Ophthalmic Epidemiol. 2016;23 sup1:46-54; doi: 10.1080/09286586.2016.1250918. https://www.ncbi.nlm.nih.gov/pubmed/27918227.

476. Molehin AJ, Rojo JU, Siddiqui SZ, Gray SA, Carter D, Siddiqui AA. Development of a schistosomiasis vaccine. Expert Rev Vaccines. 2016;15 5:619-27; doi: 10.1586/14760584.2016.1131127. https://www.ncbi.nlm.nih.gov/pubmed/26651503.

477. Mohammadpour M, Abrishami M, Masoumi A, Hashemi H. Trachoma: Past, present and future. J Curr Ophthalmol. 2016;28 4:165-9; doi: 10.1016/j.joco.2016.08.011. https://www.ncbi.nlm.nih.gov/pubmed/27830198.

478. Mieras LF, Anand S, van Brakel WH, Hamilton HC, Martin Kollmann KH, Mackenzie C, et al. Neglected Tropical Diseases, Cross-Cutting Issues Workshop, 4-6 February 2015, Utrecht, the Netherlands: meeting report. Int Health. 2016;8 Suppl 1:i7-11; doi: 10.1093/inthealth/ihw001. https://www.ncbi.nlm.nih.gov/pubmed/26940311.

479. Michael E, Singh BK. Heterogeneous dynamics, robustness/fragility trade-offs, and the eradication of the macroparasitic disease, lymphatic filariasis. BMC Med. 2016;14:14; doi: 10.1186/s12916-016-0557-y. https://www.ncbi.nlm.nih.gov/pubmed/26822124.

480. Mensah EO, Aikins MK, Gyapong M, Anto F, Bockarie MJ, Gyapong JO. Extent of Integration of Priority Interventions into General Health Systems: A Case Study of Neglected Tropical Diseases Programme in the Western Region of Ghana. PLoS Negl Trop Dis. 2016;10 5:e0004725; doi: 10.1371/journal.pntd.0004725. https://www.ncbi.nlm.nih.gov/pubmed/27203854.

481. Mengitsu B, Shafi O, Kebede B, Kebede F, Worku DT, Herero M, et al. Ethiopia and its steps to mobilize resources to achieve 2020 elimination and control goals for neglected tropical diseases webs joined can tie a lion. Int Health. 2016;8 Suppl 1:i34-52; doi: 10.1093/inthealth/ihw007. https://www.ncbi.nlm.nih.gov/pubmed/26940308.

482. Martins-Melo FR, Ramos AN, Jr., Alencar CH, Heukelbach J. Mortality from neglected tropical diseases in Brazil, 2000-2011. Bull World Health Organ. 2016;94 2:103-10; doi: 10.2471/BLT.15.152363. https://www.ncbi.nlm.nih.gov/pubmed/26908960.

483. Martins-Melo FR, Ramos AN, Jr., Alencar CH, Heukelbach J. Trends and spatial patterns of mortality related to neglected tropical diseases in Brazil. Parasite Epidemiol Control. 2016;1 2:56-65; doi: 10.1016/j.parepi.2016.03.002. https://www.ncbi.nlm.nih.gov/pubmed/29988194.

484. Marks M, Sokana O, Nachamkin E, Puiahi E, Kilua G, Pillay A, et al. Prevalence of Active and Latent Yaws in the Solomon Islands 18 Months after Azithromycin Mass Drug Administration for Trachoma. PLoS Negl Trop Dis. 2016;10 8:e0004927; doi: 10.1371/journal.pntd.0004927. https://www.ncbi.nlm.nih.gov/pubmed/27551787.

485. Lo Iacono G, Cunningham AA, Fichet-Calvet E, Garry RF, Grant DS, Leach M, et al. A Unified Framework for the Infection Dynamics of Zoonotic Spillover and Spread. PLoS Negl Trop Dis. 2016;10 9:e0004957; doi: 10.1371/journal.pntd.0004957. https://www.ncbi.nlm.nih.gov/pubmed/27588425.

486. Lenk EJ, Redekop WK, Luyendijk M, Rijnsburger AJ, Severens JL. Productivity Loss Related to Neglected Tropical Diseases Eligible for Preventive Chemotherapy: A Systematic Literature Review. PLoS Negl Trop Dis. 2016;10 2:e0004397; doi: 10.1371/journal.pntd.0004397. https://www.ncbi.nlm.nih.gov/pubmed/26890487.

487. Lemoine JF, Desormeaux AM, Monestime F, Fayette CR, Desir L, Direny AN, et al. Controlling Neglected Tropical Diseases (NTDs) in Haiti: Implementation Strategies and Evidence of Their Success. PLoS Negl Trop Dis. 2016;10 10:e0004954; doi: 10.1371/journal.pntd.0004954. https://www.ncbi.nlm.nih.gov/pubmed/27706162.

488. Lagatie O, Njumbe Ediage E, Batsa Debrah L, Diels L, Nolten C, Vinken P, et al. Evaluation of the diagnostic potential of urinary N-Acetyltyramine-O,beta-glucuronide (NATOG) as diagnostic biomarker for Onchocerca volvulus infection. Parasit Vectors. 2016;9 1:302; doi: 10.1186/s13071-016-1582-6. https://www.ncbi.nlm.nih.gov/pubmed/27216752.

489. Lachenal G, Owona Ntsama J, Ze Bekolo D, Kombang Ekodogo T, Manton J. Neglected Actors in Neglected Tropical Diseases Research: Historical Perspectives on Health Workers and Contemporary Buruli Ulcer Research in Ayos, Cameroon. PLoS Negl Trop Dis. 2016;10 4:e0004488; doi: 10.1371/journal.pntd.0004488. https://www.ncbi.nlm.nih.gov/pubmed/27101371.

490. Kuesel AC. Research for new drugs for elimination of onchocerciasis in Africa. Int J Parasitol Drugs Drug Resist. 2016;6 3:272-86; doi: 10.1016/j.ijpddr.2016.04.002. https://www.ncbi.nlm.nih.gov/pubmed/27693536.

491. Kita K, Yamada H. [The Challenge of Developing New Treatments for NTDs Originating from Japan]. Yakugaku Zasshi. 2016;136 2:203-4; doi: 10.1248/yakushi.15-00233-F. https://www.ncbi.nlm.nih.gov/pubmed/26831794.

492. Kita K. [Current Trend of Drug Development for Neglected Tropical Diseases (NTDs)]. Yakugaku Zasshi. 2016;136 2:205-11; doi: 10.1248/yakushi.15-00233-1. https://www.ncbi.nlm.nih.gov/pubmed/26831795.

493. Kisoka WJ, Tersbol BP, Meyrowitsch DW, Simonsen PE, Mushi DL. Community Members' Perceptions of Mass Drug Administration for Control of Lymphatic Filariasis in Rural and Urban Tanzania. J Biosoc Sci. 2016;48 1:94-112; doi: 10.1017/S0021932015000024. https://www.ncbi.nlm.nih.gov/pubmed/25790081.

494. Kassegne K, Abe EM, Chen JH, Zhou XN. Immunomic approaches for antigen discovery of human parasites. Expert Rev Proteomics. 2016;13 12:1091-101; doi: 10.1080/14789450.2016.1252675. https://www.ncbi.nlm.nih.gov/pubmed/27774815.

495. Houweling TA, Karim-Kos HE, Kulik MC, Stolk WA, Haagsma JA, Lenk EJ, et al. Socioeconomic Inequalities in Neglected Tropical Diseases: A Systematic Review. PLoS Negl Trop Dis. 2016;10 5:e0004546; doi: 10.1371/journal.pntd.0004546. https://www.ncbi.nlm.nih.gov/pubmed/27171166.

496. Hotez PJ, Strych U, Lustigman S, Bottazzi ME. Human anthelminthic vaccines: Rationale and challenges. Vaccine. 2016;34 30:3549-55; doi: 10.1016/j.vaccine.2016.03.112. https://www.ncbi.nlm.nih.gov/pubmed/27171753.

497. Hotez PJ, Pecoul B, Rijal S, Boehme C, Aksoy S, Malecela M, et al. Eliminating the Neglected Tropical Diseases: Translational Science and New Technologies. PLoS Negl Trop Dis. 2016;10 3:e0003895; doi: 10.1371/journal.pntd.0003895. https://www.ncbi.nlm.nih.gov/pubmed/26934395.

498. Hotez PJ, Damania A, Naghavi M. Blue Marble Health and the Global Burden of Disease Study 2013. PLoS Negl Trop Dis. 2016;10 10:e0004744; doi: 10.1371/journal.pntd.0004744. https://www.ncbi.nlm.nih.gov/pubmed/27788134.

499. Hotez PJ, Bottazzi ME, Strych U. New Vaccines for the World's Poorest People. Annu Rev Med. 2016;67:405-17; doi: 10.1146/annurev-med-051214-024241. https://www.ncbi.nlm.nih.gov/pubmed/26356803.

500. Hotez PJ, Beaumier CM, Gillespie PM, Strych U, Hayward T, Bottazzi ME. Advancing a vaccine to prevent hookworm disease and anemia. Vaccine. 2016;34 26:3001-5; doi: 10.1016/j.vaccine.2016.03.078. https://www.ncbi.nlm.nih.gov/pubmed/27040400.

501. Hopkins AD. Neglected tropical diseases in Africa: a new paradigm. Int Health. 2016;8 Suppl 1:i28-33; doi: 10.1093/inthealth/ihv077. https://www.ncbi.nlm.nih.gov/pubmed/26940307.

502. Hong Y, Cao X, Han Q, Yuan C, Zhang M, Han Y, et al. Proteome-wide analysis of lysine acetylation in adult Schistosoma japonicum worm. J Proteomics. 2016;148:202-12; doi: 10.1016/j.jprot.2016.08.008. https://www.ncbi.nlm.nih.gov/pubmed/27535354.

503. Hinoshita E. [Industry, Academia and Government Partnership through the Global Health Innovative Technology Fund (GHIT)]. Yakugaku Zasshi. 2016;136 2:237-42; doi: 10.1248/yakushi.15-00233-5. https://www.ncbi.nlm.nih.gov/pubmed/26831799.

504. Henderson RI, Hatfield J, Kutz S, Olemshumba S, Van Der Meer F, Manyama M, et al. 'We Can't Get Worms from Cow Dung': Reported Knowledge of Parasitism among Pastoralist Youth Attending Secondary School in the Ngorongoro Conservation Area, Tanzania. J Biosoc Sci. 2016;48 6:746-66; doi: 10.1017/S0021932015000358. https://www.ncbi.nlm.nih.gov/pubmed/26449343.

505. Hay R. Skin NTDs: an opportunity for integrated care. Trans R Soc Trop Med Hyg. 2016;110 12:679-80; doi: 10.1093/trstmh/trx008. https://www.ncbi.nlm.nih.gov/pubmed/28938054.

506. Hastings J. Rumours, Riots and the Rejection of Mass Drug Administration for the Treatment of Schistosomiasis in Morogoro, Tanzania. J Biosoc Sci. 2016;48 Suppl 1:S16-39; doi: 10.1017/S0021932016000018. https://www.ncbi.nlm.nih.gov/pubmed/27428064.

507. Hall MJ, Wall RL, Stevens JR. Traumatic Myiasis: A Neglected Disease in a Changing World. Annu Rev Entomol. 2016;61:159-76; doi: 10.1146/annurev-ento-010715-023655. https://www.ncbi.nlm.nih.gov/pubmed/26667275.

508. Gustavsen K, Sodahlon Y, Bush S. Cross-border collaboration for neglected tropical disease efforts-Lessons learned from onchocerciasis control and elimination in the Mano River Union (West Africa). Global Health. 2016;12 1:44; doi: 10.1186/s12992-016-0185-5. https://www.ncbi.nlm.nih.gov/pubmed/27549911.

509. Guidi A, Lalli C, Perlas E, Bolasco G, Nibbio M, Monteagudo E, et al. Discovery and Characterization of Novel Anti-schistosomal Properties of the Anti-anginal Drug, Perhexiline and Its Impact on Schistosoma mansoni Male and Female Reproductive Systems. PLoS Negl Trop Dis. 2016;10 8:e0004928; doi: 10.1371/journal.pntd.0004928. https://www.ncbi.nlm.nih.gov/pubmed/27518281.

510. Gray SA, Coler RN, Carter D, Siddiqui AA. Translational Activities to Enable NTD Vaccines. Prog Mol Biol Transl Sci. 2016;142:291-315; doi: 10.1016/bs.pmbts.2016.05.004. https://www.ncbi.nlm.nih.gov/pubmed/27571699.

511. Govindarajan M, Hoti SL, Benelli G. Facile fabrication of eco-friendly nano-mosquitocides: Biophysical characterization and effectiveness on neglected tropical mosquito vectors. Enzyme Microb Technol. 2016;95:155-63; doi: 10.1016/j.enzmictec.2016.05.005. https://www.ncbi.nlm.nih.gov/pubmed/27866611.

512. Gomes C, Pons MJ, Del Valle Mendoza J, Ruiz J. Carrion's disease: an eradicable illness? Infect Dis Poverty. 2016;5 1:105; doi: 10.1186/s40249-016-0197-7. https://www.ncbi.nlm.nih.gov/pubmed/27903286.

513. Golden A, Faulx D, Kalnoky M, Stevens E, Yokobe L, Peck R, et al. Analysis of age-dependent trends in Ov16 IgG4 seroprevalence to onchocerciasis. Parasit Vectors. 2016;9 1:338; doi: 10.1186/s13071-016-1623-1. https://www.ncbi.nlm.nih.gov/pubmed/27296630.

514. Gillespie PM, Beaumier CM, Strych U, Hayward T, Hotez PJ, Bottazzi ME. Status of vaccine research and development of vaccines for leishmaniasis. Vaccine. 2016;34 26:2992-5; doi: 10.1016/j.vaccine.2015.12.071. https://www.ncbi.nlm.nih.gov/pubmed/26973063.

515. Fleming FM, Matovu F, Hansen KS, Webster JP. A mixed methods approach to evaluating community drug distributor performance in the control of neglected tropical diseases. Parasit Vectors. 2016;9 1:345; doi: 10.1186/s13071-016-1606-2. https://www.ncbi.nlm.nih.gov/pubmed/27305942.

516. Flecker RH, O'Neal SE, Townes JM. Evaluating Healthcare Claims for Neurocysticercosis by Using All-Payer All-Claims Data, Oregon, 2010-2013. Emerg Infect Dis. 2016;22 12:2168-70; doi: 10.3201/eid2212.160370. https://www.ncbi.nlm.nih.gov/pubmed/27869593.

517. Fitzpatrick C, Fleming FM, Madin-Warburton M, Schneider T, Meheus F, Asiedu K, et al. Benchmarking the Cost per Person of Mass Treatment for Selected Neglected Tropical Diseases: An Approach Based on Literature Review and Meta-regression with Web-Based Software Application. PLoS Negl Trop Dis. 2016;10 12:e0005037; doi: 10.1371/journal.pntd.0005037. https://www.ncbi.nlm.nih.gov/pubmed/27918573.

518. Fitzpatrick C, Engels D. Leaving no one behind: a neglected tropical disease indicator and tracers for the Sustainable Development Goals. Int Health. 2016;8 Suppl 1:i15-8; doi: 10.1093/inthealth/ihw002. https://www.ncbi.nlm.nih.gov/pubmed/26940304.

519. Fitzpatrick C, Bangert M, Engels D. Sustainable Development Goals: diseases that neglect no goals. Nature. 2016;535 7613:493; doi: 10.1038/535493c. https://www.ncbi.nlm.nih.gov/pubmed/27466114.

520. Figueiredo AB, Souza-Testasicca MC, Afonso LCC. Purinergic signaling and infection by Leishmania: A new approach to evasion of the immune response. Biomed J. 2016;39 4:244-50; doi: 10.1016/j.bj.2016.08.004. https://www.ncbi.nlm.nih.gov/pubmed/27793266.

521. F ESR, Ferreira LF, Hernandes MZ, de Brito ME, de Oliveira BC, da Silva AA, et al. Combination of In Silico Methods in the Search for Potential CD4(+) and CD8(+) T Cell Epitopes in the Proteome of Leishmania braziliensis. Front Immunol. 2016;7:327; doi: 10.3389/fimmu.2016.00327. https://www.ncbi.nlm.nih.gov/pubmed/27621732.

522. Engels D. Neglected tropical diseases in the Sustainable Development Goals. Lancet. 2016;387 10015:223-4; doi: 10.1016/S0140-6736(16)00043-X. https://www.ncbi.nlm.nih.gov/pubmed/26842291.

523. Engels D. The Global Trachoma Mapping Project: A Catalyst for Progress Against Neglected Tropical Diseases. Ophthalmic Epidemiol. 2016;23 sup1:1-2; doi: 10.1080/09286586.2016.1257139. https://www.ncbi.nlm.nih.gov/pubmed/28030282.

524. Engelman D, Fuller LC, Solomon AW, McCarthy JS, Hay RJ, Lammie PJ, et al. Opportunities for Integrated Control of Neglected Tropical Diseases That Affect the Skin. Trends Parasitol. 2016;32 11:843-54; doi: 10.1016/j.pt.2016.08.005. https://www.ncbi.nlm.nih.gov/pubmed/27638231.

525. Elyana FN, Al-Mekhlafi HM, Ithoi I, Abdulsalam AM, Dawaki S, Nasr NA, et al. A tale of two communities: intestinal polyparasitism among Orang Asli and Malay communities in rural Terengganu, Malaysia. Parasit Vectors. 2016;9 1:398; doi: 10.1186/s13071-016-1678-z. https://www.ncbi.nlm.nih.gov/pubmed/27422533.

526. Elshafie AI, Mullazehi M, Ronnelid J. General false positive ELISA reactions in visceral leishmaniasis. Implications for the use of enzyme immunoassay analyses in tropical Africa. J Immunol Methods. 2016;431:66-71; doi: 10.1016/j.jim.2016.02.007. https://www.ncbi.nlm.nih.gov/pubmed/26859242.

527. Eibach D, Krumkamp R, Hahn A, Sarpong N, Adu-Sarkodie Y, Leva A, et al. Application of a multiplex PCR assay for the detection of gastrointestinal pathogens in a rural African setting. BMC Infect Dis. 2016;16:150; doi: 10.1186/s12879-016-1481-7. https://www.ncbi.nlm.nih.gov/pubmed/27080387.

528. Dixit R, Herz J, Dalton R, Booy R. Benefits of using heterologous polyclonal antibodies and potential applications to new and undertreated infectious pathogens. Vaccine. 2016;34 9:1152-61; doi: 10.1016/j.vaccine.2016.01.016. https://www.ncbi.nlm.nih.gov/pubmed/26802604.

529. Dias JC, Ramos AN, Jr., Gontijo ED, Luquetti A, Shikanai-Yasuda MA, Coura JR, et al. [Brazilian Consensus on Chagas Disease, 2015]. Epidemiol Serv Saude. 2016;25 spe:7-86; doi: 10.5123/S1679-49742016000500002. https://www.ncbi.nlm.nih.gov/pubmed/27869914.

530. Devleesschauwer B, Aryal A, Sharma BK, Ale A, Declercq A, Depraz S, et al. Epidemiology, Impact and Control of Rabies in Nepal: A Systematic Review. PLoS Negl Trop Dis. 2016;10 2:e0004461; doi: 10.1371/journal.pntd.0004461. https://www.ncbi.nlm.nih.gov/pubmed/26871689.

531. Deribe K. The Countdown to 2020: measuring progress in neglected tropical diseases. Lancet Glob Health. 2016;4 3:e163; doi: 10.1016/S2214-109X(15)00319-8. https://www.ncbi.nlm.nih.gov/pubmed/26852339.

532. Dean L, Page S, Hawkins K, Stothard R, Thomson R, Wanji S, et al. Tailoring mass drug administration to context: implementation research is critical in achieving equitable progress in the control and elimination of helminth neglected tropical diseases in sub-Saharan Africa. Int Health. 2016;8 4:233-4; doi: 10.1093/inthealth/ihw031. https://www.ncbi.nlm.nih.gov/pubmed/27481833.

533. de Vlas SJ, Stolk WA, le Rutte EA, Hontelez JA, Bakker R, Blok DJ, et al. Concerted Efforts to Control or Eliminate Neglected Tropical Diseases: How Much Health Will Be Gained? PLoS Negl Trop Dis. 2016;10 2:e0004386; doi: 10.1371/journal.pntd.0004386. https://www.ncbi.nlm.nih.gov/pubmed/26890362.

534. de Souza DK, Yirenkyi E, Otchere J, Biritwum NK, Ameme DK, Sackey S, et al. Assessing Lymphatic Filariasis Data Quality in Endemic Communities in Ghana, Using the Neglected Tropical Diseases Data Quality Assessment Tool for Preventive Chemotherapy. PLoS Negl Trop Dis. 2016;10 3:e0004590; doi: 10.1371/journal.pntd.0004590. https://www.ncbi.nlm.nih.gov/pubmed/27028010.

535. Das VN, Pandey RN, Siddiqui NA, Chapman LA, Kumar V, Pandey K, et al. Longitudinal Study of Transmission in Households with Visceral Leishmaniasis, Asymptomatic Infections and PKDL in Highly Endemic Villages in Bihar, India. PLoS Negl Trop Dis. 2016;10 12:e0005196; doi: 10.1371/journal.pntd.0005196. https://www.ncbi.nlm.nih.gov/pubmed/27974858.

536. Corley AG, Thornton CP, Glass NE. The Role of Nurses and Community Health Workers in Confronting Neglected Tropical Diseases in Sub-Saharan Africa: A Systematic Review. PLoS Negl Trop Dis. 2016;10 9:e0004914; doi: 10.1371/journal.pntd.0004914. https://www.ncbi.nlm.nih.gov/pubmed/27631980.

537. Cohen JP, Silva L, Cohen A, Awatin J, Sturgeon R. Progress Report on Neglected Tropical Disease Drug Donation Programs. Clin Ther. 2016;38 5:1193-204; doi: 10.1016/j.clinthera.2016.02.031. https://www.ncbi.nlm.nih.gov/pubmed/27041410.

538. Cocks N, Rainima-Qaniuci M, Yalen C, Macleod C, Nakolinivalu A, Migchelsen S, et al. Community seroprevalence survey for yaws and trachoma in the Western Division of Fiji. Trans R Soc Trop Med Hyg. 2016;110 10:582-7; doi: 10.1093/trstmh/trw069. https://www.ncbi.nlm.nih.gov/pubmed/27852877.

539. Christinet V, Lazdins-Helds JK, Stothard JR, Reinhard-Rupp J. Female genital schistosomiasis (FGS): from case reports to a call for concerted action against this neglected gynaecological disease. Int J Parasitol. 2016;46 7:395-404; doi: 10.1016/j.ijpara.2016.02.006. https://www.ncbi.nlm.nih.gov/pubmed/27063073.

540. Cardoso CS, Sabino EC, Oliveira CD, de Oliveira LC, Ferreira AM, Cunha-Neto E, et al. Longitudinal study of patients with chronic Chagas cardiomyopathy in Brazil (SaMi-Trop project): a cohort profile. BMJ Open. 2016;6 5:e011181; doi: 10.1136/bmjopen-2016-011181. https://www.ncbi.nlm.nih.gov/pubmed/27147390.

541. Canavati SE, Lawford HL, Fatunmbi BS, Lek D, Top-Samphor N, Leang R, et al. Establishing research priorities for malaria elimination in the context of the emergency response to artemisinin resistance framework-the Cambodian approach. Malar J. 2016;15:120; doi: 10.1186/s12936-016-1117-9. https://www.ncbi.nlm.nih.gov/pubmed/26916933.

542. Cai P, Gobert GN, You H, McManus DP. The Tao survivorship of schistosomes: implications for schistosomiasis control. Int J Parasitol. 2016;46 7:453-63; doi: 10.1016/j.ijpara.2016.01.002. https://www.ncbi.nlm.nih.gov/pubmed/26873753.

543. Budge PJ, Sognikin E, Akosa A, Mathieu EM, Deming M. Accuracy of Coverage Survey Recall following an Integrated Mass Drug Administration for Lymphatic Filariasis, Schistosomiasis, and Soil-Transmitted Helminthiasis. PLoS Negl Trop Dis. 2016;10 1:e0004358; doi: 10.1371/journal.pntd.0004358. https://www.ncbi.nlm.nih.gov/pubmed/26766287.

544. Bottomley C, Isham V, Vivas-Martinez S, Kuesel AC, Attah SK, Opoku NO, et al. Modelling Neglected Tropical Diseases diagnostics: the sensitivity of skin snips for Onchocerca volvulus in near elimination and surveillance settings. Parasit Vectors. 2016;9 1:343; doi: 10.1186/s13071-016-1605-3. https://www.ncbi.nlm.nih.gov/pubmed/27301567.

545. Botelho MC, Machado A, Carvalho A, Vilaca M, Conceicao O, Rosa F, et al. Schistosoma haematobium in Guinea-Bissau: unacknowledged morbidity due to a particularly neglected parasite in a particularly neglected country. Parasitol Res. 2016;115 4:1567-72; doi: 10.1007/s00436-015-4891-3. https://www.ncbi.nlm.nih.gov/pubmed/26755362.

546. Boko PM, Ibikounle M, Onzo-Aboki A, Tougoue JJ, Sissinto Y, Batcho W, et al. Schistosomiasis and Soil Transmitted Helminths Distribution in Benin: A Baseline Prevalence Survey in 30 Districts. PLoS One. 2016;11 9:e0162798; doi: 10.1371/journal.pone.0162798. https://www.ncbi.nlm.nih.gov/pubmed/27643795.

547. Boisson S, Engels D, Gordon BA, Medlicott KO, Neira MP, Montresor A, et al. Water, sanitation and hygiene for accelerating and sustaining progress on neglected tropical diseases: a new Global Strategy 2015-20. Int Health. 2016;8 Suppl 1:i19-21; doi: 10.1093/inthealth/ihv073. https://www.ncbi.nlm.nih.gov/pubmed/26940305.

548. Bogus J, Gankpala L, Fischer K, Krentel A, Weil GJ, Fischer PU, et al. Community Attitudes Toward Mass Drug Administration for Control and Elimination of Neglected Tropical Diseases After the 2014 Outbreak of Ebola Virus Disease in Lofa County, Liberia. Am J Trop Med Hyg. 2016;94 3:497-503; doi: 10.4269/ajtmh.15-0591. https://www.ncbi.nlm.nih.gov/pubmed/26666700.

549. Blasco-Hernandez T, Garcia-San Miguel L, Navaza B, Navarro M, Benito A. Knowledge and experiences of Chagas disease in Bolivian women living in Spain: a qualitative study. Glob Health Action. 2016;9:30201; doi: 10.3402/gha.v9.30201. https://www.ncbi.nlm.nih.gov/pubmed/26976265.

550. Benelli G. Plant-mediated biosynthesis of nanoparticles as an emerging tool against mosquitoes of medical and veterinary importance: a review. Parasitol Res. 2016;115 1:23-34; doi: 10.1007/s00436-015-4800-9. https://www.ncbi.nlm.nih.gov/pubmed/26541154.

551. Becker SL, Yap P, Horie NS, Alirol E, Barbe B, Bhatta NK, et al. Experiences and Lessons from a Multicountry NIDIAG Study on Persistent Digestive Disorders in the Tropics. PLoS Negl Trop Dis. 2016;10 11:e0004818; doi: 10.1371/journal.pntd.0004818. https://www.ncbi.nlm.nih.gov/pubmed/27812101.

552. Basanez MG, Walker M, Turner HC, Coffeng LE, de Vlas SJ, Stolk WA. River Blindness: Mathematical Models for Control and Elimination. Adv Parasitol. 2016;94:247-341; doi: 10.1016/bs.apar.2016.08.003. https://www.ncbi.nlm.nih.gov/pubmed/27756456.

553. Basanez MG, Anderson RM. Preface. Adv Parasitol. 2016;94:xi-xvi; doi: 10.1016/S0065-308X(16)30098-7. https://www.ncbi.nlm.nih.gov/pubmed/27756460.

554. Bartsch SM, Hotez PJ, Asti L, Zapf KM, Bottazzi ME, Diemert DJ, et al. The Global Economic and Health Burden of Human Hookworm Infection. PLoS Negl Trop Dis. 2016;10 9:e0004922; doi: 10.1371/journal.pntd.0004922. https://www.ncbi.nlm.nih.gov/pubmed/27607360.

555. Barry MA, Murray KO, Hotez PJ, Jones KM. Impact of vectorborne parasitic neglected tropical diseases on child health. Arch Dis Child. 2016;101 7:640-7; doi: 10.1136/archdischild-2015-308266. https://www.ncbi.nlm.nih.gov/pubmed/26921274.

556. Bardosh KL. Deadly Flies, Poor Profits, and Veterinary Pharmaceuticals: Sustaining the Control of Sleeping Sickness in Uganda. Med Anthropol. 2016;35 4:338-52; doi: 10.1080/01459740.2015.1101461. https://www.ncbi.nlm.nih.gov/pubmed/26457971.

557. Ballesteros C, Tritten L, O'Neill M, Burkman E, Zaky WI, Xia J, et al. The Effects of Ivermectin on Brugia malayi Females In Vitro: A Transcriptomic Approach. PLoS Negl Trop Dis. 2016;10 8:e0004929; doi: 10.1371/journal.pntd.0004929. https://www.ncbi.nlm.nih.gov/pubmed/27529747.

558. Bajiro M, Dana D, Ayana M, Emana D, Mekonnen Z, Zawdie B, et al. Prevalence of Schistosoma mansoni infection and the therapeutic efficacy of praziquantel among school children in Manna District, Jimma Zone, southwest Ethiopia. Parasit Vectors. 2016;9 1:560; doi: 10.1186/s13071-016-1833-6. https://www.ncbi.nlm.nih.gov/pubmed/27772528.

559. Ayode D, Tora A, Farrell D, Tadele G, Davey G, McBride CM. Association Between Causal Beliefs and Shoe Wearing to Prevent Podoconiosis: A Baseline Study. Am J Trop Med Hyg. 2016;94 5:1123-8; doi: 10.4269/ajtmh.15-0342. https://www.ncbi.nlm.nih.gov/pubmed/26928843.

560. Aung AK, Spelman DW. Taenia solium Taeniasis and Cysticercosis in Southeast Asia. Am J Trop Med Hyg. 2016;94 5:947-54; doi: 10.4269/ajtmh.15-0684. https://www.ncbi.nlm.nih.gov/pubmed/26834197.

561. Asada M. [Development and Distribution of Drugs for NTDs: Efforts of One Pharmaceutical Company]. Yakugaku Zasshi. 2016;136 2:223-30; doi: 10.1248/yakushi.15-00233-3. https://www.ncbi.nlm.nih.gov/pubmed/26831797.

562. Armstrong SD, Xia D, Bah GS, Krishna R, Ngangyung HF, LaCourse EJ, et al. Stage-specific Proteomes from Onchocerca ochengi, Sister Species of the Human River Blindness Parasite, Uncover Adaptations to a Nodular Lifestyle. Mol Cell Proteomics. 2016;15 8:2554-75; doi: 10.1074/mcp.M115.055640. https://www.ncbi.nlm.nih.gov/pubmed/27226403.

563. Alirol E, Horie NS, Barbe B, Lejon V, Verdonck K, Gillet P, et al. Diagnosis of Persistent Fever in the Tropics: Set of Standard Operating Procedures Used in the NIDIAG Febrile Syndrome Study. PLoS Negl Trop Dis. 2016;10 11:e0004749; doi: 10.1371/journal.pntd.0004749. https://www.ncbi.nlm.nih.gov/pubmed/27812090.

564. Agbor VN, Njim T, Mbolingong FN. Bladder outlet obstruction; a rare complication of the neglected schistosome, Schistosoma haematobium: two case reports and public health challenges. BMC Res Notes. 2016;9 1:493; doi: 10.1186/s13104-016-2303-0. https://www.ncbi.nlm.nih.gov/pubmed/27876076.

565. Afework Bitew A, Abera B, Seyoum W, Endale B, Kiber T, Goshu G, et al. Soil-Transmitted Helminths and Schistosoma mansoni Infections in Ethiopian Orthodox Church Students around Lake Tana, Northwest Ethiopia. PLoS One. 2016;11 5:e0155915; doi: 10.1371/journal.pone.0155915. https://www.ncbi.nlm.nih.gov/pubmed/27203749.

566. Afakye K, Kenu E, Nyarko KM, Johnson SA, Wongnaah F, Bonsu GK. Household exposure and animal-bite surveillance following human rabies detection in Southern Ghana. Pan Afr Med J. 2016;25 Suppl 1:12; doi: 10.11604/pamj.supp.2016.25.1.6200. https://www.ncbi.nlm.nih.gov/pubmed/28149437.

567. Abdul-Ghani R, Mahdy MAK, Beier JC. Onchocerciasis in Yemen: Time to take action against a neglected tropical parasitic disease. Acta Trop. 2016;162:133-41; doi: 10.1016/j.actatropica.2016.06.017. https://www.ncbi.nlm.nih.gov/pubmed/27325293.

568. Neglected tropical diseases attract attention in the Lords. Vet Rec. 2016;178 7:156; doi: 10.1136/vr.i829. https://www.ncbi.nlm.nih.gov/pubmed/26868232.

569. Neglected Tropical Diseases: Research Bites Back. EBioMedicine. 2016;11:1; doi: 10.1016/j.ebiom.2016.09.009. https://www.ncbi.nlm.nih.gov/pubmed/27692288.

570. Zicker F, Faid M, Reeder J, Aslanyan G. Building coherence and synergy among global health initiatives. Health Res Policy Syst. 2015;13:75; doi: 10.1186/s12961-015-0062-3. https://www.ncbi.nlm.nih.gov/pubmed/26652173.

571. Zhou XN, Olveda R, Sripa B, Yang GJ, Leonardo L, Bergquist R. From gap analysis to solution and action: the RNAS(+) model. Acta Trop. 2015;141 Pt B:146-9; doi: 10.1016/j.actatropica.2013.06.016. https://www.ncbi.nlm.nih.gov/pubmed/23831926.

572. Yamamoto ES, Campos BL, Jesus JA, Laurenti MD, Ribeiro SP, Kallas EG, et al. The Effect of Ursolic Acid on Leishmania (Leishmania) amazonensis Is Related to Programed Cell Death and Presents Therapeutic Potential in Experimental Cutaneous Leishmaniasis. PLoS One. 2015;10 12:e0144946; doi: 10.1371/journal.pone.0144946. https://www.ncbi.nlm.nih.gov/pubmed/26674781.

573. Walz Y, Wegmann M, Dech S, Raso G, Utzinger J. Risk profiling of schistosomiasis using remote sensing: approaches, challenges and outlook. Parasit Vectors. 2015;8:163; doi: 10.1186/s13071-015-0732-6. https://www.ncbi.nlm.nih.gov/pubmed/25890278.

574. Utzinger J, Becker SL, van Lieshout L, van Dam GJ, Knopp S. New diagnostic tools in schistosomiasis. Clin Microbiol Infect. 2015;21 6:529-42; doi: 10.1016/j.cmi.2015.03.014. https://www.ncbi.nlm.nih.gov/pubmed/25843503.

575. Tuhebwe D, Bagonza J, Kiracho EE, Yeka A, Elliott AM, Nuwaha F. Uptake of mass drug administration programme for schistosomiasis control in Koome Islands, Central Uganda. PLoS One. 2015;10 4:e0123673; doi: 10.1371/journal.pone.0123673. https://www.ncbi.nlm.nih.gov/pubmed/25830917.

576. Toledo R, Munoz-Antoli C, Esteban JG. Strongyloidiasis with emphasis on human infections and its different clinical forms. Adv Parasitol. 2015;88:165-241; doi: 10.1016/bs.apar.2015.02.005. https://www.ncbi.nlm.nih.gov/pubmed/25911368.

577. Terry FE, Moise L, Martin RF, Torres M, Pilotte N, Williams SA, et al. Time for T? Immunoinformatics addresses vaccine design for neglected tropical and emerging infectious diseases. Expert Rev Vaccines. 2015;14 1:21-35; doi: 10.1586/14760584.2015.955478. https://www.ncbi.nlm.nih.gov/pubmed/25193104.

578. Tarleton RL. CD8+ T cells in Trypanosoma cruzi infection. Semin Immunopathol. 2015;37 3:233-8; doi: 10.1007/s00281-015-0481-9. https://www.ncbi.nlm.nih.gov/pubmed/25921214.

579. Sutherland CS, Yukich J, Goeree R, Tediosi F. A literature review of economic evaluations for a neglected tropical disease: human African trypanosomiasis ("sleeping sickness"). PLoS Negl Trop Dis. 2015;9 2:e0003397; doi: 10.1371/journal.pntd.0003397. https://www.ncbi.nlm.nih.gov/pubmed/25654605.

580. Stamm LV. Yaws: 110 years after Castellani's discovery of Treponema pallidum subspecies pertenue. Am J Trop Med Hyg. 2015;93 1:4-6; doi: 10.4269/ajtmh.15-0147. https://www.ncbi.nlm.nih.gov/pubmed/25870417.

581. Staff PNTD. Correction: Standardizing visual control devices for tsetse flies: East African species Glossina fuscipes fuscipes and Glossina tachinoides. PLoS Negl Trop Dis. 2015;9 3:e0003582; doi: 10.1371/journal.pntd.0003582. https://www.ncbi.nlm.nih.gov/pubmed/25790438.

582. Staff PNTD. Correction: Pyrethroid Treatment of Cattle for Tsetse Control: Reducing Its Impact on Dung Fauna. PLoS Negl Trop Dis. 2015;9 6:e0003830; doi: 10.1371/journal.pntd.0003830. https://www.ncbi.nlm.nih.gov/pubmed/26090856.

583. Sousa GR, Costa HS, Souza AC, Nunes MC, Lima MM, Rocha MO. Health-related quality of life in patients with Chagas disease: a review of the evidence. Rev Soc Bras Med Trop. 2015;48 2:121-8; doi: 10.1590/0037-8682-0244-2014. https://www.ncbi.nlm.nih.gov/pubmed/25992924.

584. Sol Gaspe M, Provecho YM, Cardinal MV, del Pilar Fernandez M, Gurtler RE. Ecological and sociodemographic determinants of house infestation by Triatoma infestans in indigenous communities of the Argentine Chaco. PLoS Negl Trop Dis. 2015;9 3:e0003614; doi: 10.1371/journal.pntd.0003614. https://www.ncbi.nlm.nih.gov/pubmed/25785439.

585. Smith J, Taylor EM, Kingsley P. One World-One Health and neglected zoonotic disease: elimination, emergence and emergency in Uganda. Soc Sci Med. 2015;129:12-9; doi: 10.1016/j.socscimed.2014.06.044. https://www.ncbi.nlm.nih.gov/pubmed/24984807.

586. Simarro PP, Cecchi G, Franco JR, Paone M, Diarra A, Priotto G, et al. Monitoring the Progress towards the Elimination of Gambiense Human African Trypanosomiasis. PLoS Negl Trop Dis. 2015;9 6:e0003785; doi: 10.1371/journal.pntd.0003785. https://www.ncbi.nlm.nih.gov/pubmed/26056823.

587. Savoia D. Recent updates and perspectives on leishmaniasis. J Infect Dev Ctries. 2015;9 6:588-96; doi: 10.3855/jidc.6833. https://www.ncbi.nlm.nih.gov/pubmed/26142667.

588. Sady H, Al-Mekhlafi HM, Webster BL, Ngui R, Atroosh WM, Al-Delaimy AK, et al. New insights into the genetic diversity of Schistosoma mansoni and S. haematobiumin Yemen. Parasit Vectors. 2015;8:544; doi: 10.1186/s13071-015-1168-8. https://www.ncbi.nlm.nih.gov/pubmed/26482435.

589. Rupprecht CE, Burgess GW. Viral and vector zoonotic exploitation of a homo-sociome memetic complex. Clin Microbiol Infect. 2015;21 5:394-403; doi: 10.1016/j.cmi.2015.02.032. https://www.ncbi.nlm.nih.gov/pubmed/25769428.

590. Ruiz JP, Nyingilili HS, Mbata GH, Malele, II. The role of domestic animals in the epidemiology of human African trypanosomiasis in Ngorongoro conservation area, Tanzania. Parasit Vectors. 2015;8:510; doi: 10.1186/s13071-015-1125-6. https://www.ncbi.nlm.nih.gov/pubmed/26444416.

591. Rojas-Caraballo J, Lopez-Aban J, Fernandez-Soto P, Vicente B, Collia F, Muro A. Gene Expression Profile in the Liver of BALB/c Mice Infected with Fasciola hepatica. PLoS One. 2015;10 8:e0134910; doi: 10.1371/journal.pone.0134910. https://www.ncbi.nlm.nih.gov/pubmed/26247779.

592. Rodrigues MM, Ersching J. Neglected tropical diseases, bioinformatics, and vaccines. J Infect Dis. 2015;211 2:175-7; doi: 10.1093/infdis/jiu420. https://www.ncbi.nlm.nih.gov/pubmed/25070940.

593. Rezza G. Do we need a vaccine against chikungunya? Pathog Glob Health. 2015;109 4:170-3; doi: 10.1179/2047773215Y.0000000017. https://www.ncbi.nlm.nih.gov/pubmed/25971340.

594. Ramos Junior AN, Heukelbach J. Yellow fever risk assessment in the Central African Republic. Trans R Soc Trop Med Hyg. 2015;109 4:231-2; doi: 10.1093/trstmh/trv011. https://www.ncbi.nlm.nih.gov/pubmed/25732754.

595. Polman K, Becker SL, Alirol E, Bhatta NK, Bhattarai NR, Bottieau E, et al. Erratum to: Diagnosis of neglected tropical diseases among patients with persistent digestive disorders (diarrhoea and/or abdominal pain >/=14 days): a multi-country, prospective, non-experimental case-control study. BMC Infect Dis. 2015;15:499; doi: 10.1186/s12879-015-1160-0. https://www.ncbi.nlm.nih.gov/pubmed/26537896.

596. Otabil KB, Tenkorang SB. Filarial hydrocele: a neglected condition of a neglected tropical disease. J Infect Dev Ctries. 2015;9 5:456-62; doi: 10.3855/jidc.5346. https://www.ncbi.nlm.nih.gov/pubmed/25989164.

597. Oluwole AS, Ekpo UF, Karagiannis-Voules DA, Abe EM, Olamiju FO, Isiyaku S, et al. Bayesian geostatistical model-based estimates of soil-transmitted helminth infection in Nigeria, including annual deworming requirements. PLoS Negl Trop Dis. 2015;9 4:e0003740; doi: 10.1371/journal.pntd.0003740. https://www.ncbi.nlm.nih.gov/pubmed/25909633.

598. Okello A, Welburn S, Smith J. Crossing institutional boundaries: mapping the policy process for improved control of endemic and neglected zoonoses in sub-Saharan Africa. Health Policy Plan. 2015;30 6:804-12; doi: 10.1093/heapol/czu059. https://www.ncbi.nlm.nih.gov/pubmed/25000963.

599. Nuss AB, Ejendal KF, Doyle TB, Meyer JM, Lang EG, Watts VJ, et al. Dopamine receptor antagonists as new mode-of-action insecticide leads for control of Aedes and Culex mosquito vectors. PLoS Negl Trop Dis. 2015;9 3:e0003515; doi: 10.1371/journal.pntd.0003515. https://www.ncbi.nlm.nih.gov/pubmed/25793586.

600. Nikolay B, Mwandawiro CS, Kihara JH, Okoyo C, Cano J, Mwanje MT, et al. Understanding Heterogeneity in the Impact of National Neglected Tropical Disease Control Programmes: Evidence from School-Based Deworming in Kenya. PLoS Negl Trop Dis. 2015;9 9:e0004108; doi: 10.1371/journal.pntd.0004108. https://www.ncbi.nlm.nih.gov/pubmed/26421808.

601. Neves BJ, Andrade CH, Cravo PV. Natural products as leads in schistosome drug discovery. Molecules. 2015;20 2:1872-903; doi: 10.3390/molecules20021872. https://www.ncbi.nlm.nih.gov/pubmed/25625682.

602. Negussie H, Kassahun MM, Fegan G, Njuguna P, Enquselassie F, McKay A, et al. Podoconiosis treatment in northern Ethiopia (GoLBet): study protocol for a randomised controlled trial. Trials. 2015;16:307; doi: 10.1186/s13063-015-0818-7. https://www.ncbi.nlm.nih.gov/pubmed/26177812.

603. Nalugwa A, Olsen A, Tukahebwa ME, Nuwaha F. Intestinal schistosomiasis among preschool children along the shores of Lake Victoria in Uganda. Acta Trop. 2015;142:115-21; doi: 10.1016/j.actatropica.2014.11.014. https://www.ncbi.nlm.nih.gov/pubmed/25454166.

604. Nakagawa J, Ehrenberg JP, Nealon J, Furst T, Aratchige P, Gonzales G, et al. Towards effective prevention and control of helminth neglected tropical diseases in the Western Pacific Region through multi-disease and multi-sectoral interventions. Acta Trop. 2015;141 Pt B:407-18; doi: 10.1016/j.actatropica.2013.05.010. https://www.ncbi.nlm.nih.gov/pubmed/23792012.

605. Mugambi RM, Agola EL, Mwangi IN, Kinyua J, Shiraho EA, Mkoji GM. Development and evaluation of a Loop Mediated Isothermal Amplification (LAMP) technique for the detection of hookworm (Necator americanus) infection in fecal samples. Parasit Vectors. 2015;8:574; doi: 10.1186/s13071-015-1183-9. https://www.ncbi.nlm.nih.gov/pubmed/26546069.

606. Mousley E, Deribe K, Tamiru A, Tomczyk S, Hanlon C, Davey G. Mental distress and podoconiosis in Northern Ethiopia: a comparative cross-sectional study. Int Health. 2015;7 1:16-25; doi: 10.1093/inthealth/ihu043. https://www.ncbi.nlm.nih.gov/pubmed/25062906.

607. Moraga P, Cano J, Baggaley RF, Gyapong JO, Njenga SM, Nikolay B, et al. Modelling the distribution and transmission intensity of lymphatic filariasis in sub-Saharan Africa prior to scaling up interventions: integrated use of geostatistical and mathematical modelling. Parasit Vectors. 2015;8:560; doi: 10.1186/s13071-015-1166-x. https://www.ncbi.nlm.nih.gov/pubmed/26496983.

608. Molyneux D. The Theory of Everything and a neglected tropical disease: a Hawking legacy. Lancet. 2015;385 9973:1069-70; doi: 10.1016/S0140-6736(15)60301-4. https://www.ncbi.nlm.nih.gov/pubmed/25749642.

609. Mofid LS, Casapia M, Montresor A, Rahme E, Fraser WD, Marquis GS, et al. Maternal Deworming Research Study (MADRES) protocol: a double-blind, placebo-controlled randomised trial to determine the effectiveness of deworming in the immediate postpartum period. BMJ Open. 2015;5 6:e008560; doi: 10.1136/bmjopen-2015-008560. https://www.ncbi.nlm.nih.gov/pubmed/26084556.

610. Mishra A, Antony JS, Sundaravadivel P, Tong HV, Meyer CG, Jalli RD, et al. Association of Ficolin-2 Serum Levels and FCN2 Genetic Variants with Indian Visceral Leishmaniasis. PLoS One. 2015;10 5:e0125940; doi: 10.1371/journal.pone.0125940. https://www.ncbi.nlm.nih.gov/pubmed/25965808.

611. Meurs L, Brienen E, Mbow M, Ochola EA, Mboup S, Karanja DM, et al. Is PCR the Next Reference Standard for the Diagnosis of Schistosoma in Stool? A Comparison with Microscopy in Senegal and Kenya. PLoS Negl Trop Dis. 2015;9 7:e0003959; doi: 10.1371/journal.pntd.0003959. https://www.ncbi.nlm.nih.gov/pubmed/26217948.

612. Meemon K, Sobhon P. Juvenile-specific cathepsin proteases in Fasciola spp.: their characteristics and vaccine efficacies. Parasitol Res. 2015;114 8:2807-13; doi: 10.1007/s00436-015-4589-6. https://www.ncbi.nlm.nih.gov/pubmed/26099239.

613. McCarthy M. Science academies of G7 nations call for action on antibiotic resistance and neglected tropical diseases. BMJ. 2015;350:h2346; doi: 10.1136/bmj.h2346. https://www.ncbi.nlm.nih.gov/pubmed/25929670.

614. Marks M, Mitja O, Solomon AW, Asiedu KB, Mabey DC. Yaws. Br Med Bull. 2015;113:91-100; doi: 10.1093/bmb/ldu037. https://www.ncbi.nlm.nih.gov/pubmed/25525120.

615. Macpherson EE, Adams ER, Bockarie MJ, Hollingsworth TD, Kelly-Hope LA, Lehane M, et al. Mass Drug Administration and beyond: how can we strengthen health systems to deliver complex interventions to eliminate neglected tropical diseases? BMC Proc. 2015;9 Suppl 10:S7; doi: 10.1186/1753-6561-9-S10-S7. https://www.ncbi.nlm.nih.gov/pubmed/28281705.

616. Li Y, Wu S. Dengue: what it is and why there is more. Sci Bull Sci Found Philipp. 2015;60 7:661-4; doi: 10.1007/s11434-015-0756-5. https://www.ncbi.nlm.nih.gov/pubmed/26640300.

617. Leow CY, Willis C, Hofmann A, Jones MK. Structure-function analysis of apical membrane-associated molecules of the tegument of schistosome parasites of humans: prospects for identification of novel targets for parasite control. Br J Pharmacol. 2015;172 7:1653-63; doi: 10.1111/bph.12898. https://www.ncbi.nlm.nih.gov/pubmed/25176442.

618. Lee BY, Bartsch SM, Gorham KM. Economic and financial evaluation of neglected tropical diseases. Adv Parasitol. 2015;87:329-417; doi: 10.1016/bs.apar.2015.01.002. https://www.ncbi.nlm.nih.gov/pubmed/25765199.

619. Lamberton PH, Crellen T, Cotton JA, Webster JP. Modelling the effects of mass drug administration on the molecular epidemiology of schistosomes. Adv Parasitol. 2015;87:293-327; doi: 10.1016/bs.apar.2014.12.006. https://www.ncbi.nlm.nih.gov/pubmed/25765198.

620. Koporc KM, Strunz E, Holloway C, Addiss DG, Lin W. Assessing "First Mile" Supply Chain Factors Affecting Timeliness of School-Based Deworming Interventions: Supply and Logistics Performance Indicators. PLoS Negl Trop Dis. 2015;9 12:e0004115; doi: 10.1371/journal.pntd.0004115. https://www.ncbi.nlm.nih.gov/pubmed/26657842.

621. Kone S, Baikoro N, N'Guessan Y, Jaeger FN, Silue KD, Furst T, et al. Health & Demographic Surveillance System Profile: The Taabo Health and Demographic Surveillance System, Cote d'Ivoire. Int J Epidemiol. 2015;44 1:87-97; doi: 10.1093/ije/dyu221. https://www.ncbi.nlm.nih.gov/pubmed/25433704.

622. Karagiannis-Voules DA, Odermatt P, Biedermann P, Khieu V, Schar F, Muth S, et al. Geostatistical modelling of soil-transmitted helminth infection in Cambodia: do socioeconomic factors improve predictions? Acta Trop. 2015;141 Pt B:204-12; doi: 10.1016/j.actatropica.2014.09.001. https://www.ncbi.nlm.nih.gov/pubmed/25205492.

623. Karagiannis-Voules DA, Biedermann P, Ekpo UF, Garba A, Langer E, Mathieu E, et al. Spatial and temporal distribution of soil-transmitted helminth infection in sub-Saharan Africa: a systematic review and geostatistical meta-analysis. Lancet Infect Dis. 2015;15 1:74-84; doi: 10.1016/S1473-3099(14)71004-7. https://www.ncbi.nlm.nih.gov/pubmed/25486852.

624. Kalua K, Phiri M, Kumwenda I, Masika M, Pavluck AL, Willis R, et al. Baseline Trachoma Mapping in Malawi with the Global Trachoma Mapping Project (GTMP). Ophthalmic Epidemiol. 2015;22 3:176-83; doi: 10.3109/09286586.2015.1035793. https://www.ncbi.nlm.nih.gov/pubmed/26158575.

625. Kaatano GM, Min DY, Siza JE, Yong TS, Chai JY, Ko Y, et al. Schistosoma mansoni-Related Hepatosplenic Morbidity in Adult Population on Kome Island, Sengerema District, Tanzania. Korean J Parasitol. 2015;53 5:545-51; doi: 10.3347/kjp.2015.53.5.545. https://www.ncbi.nlm.nih.gov/pubmed/26537033.

626. Johnson RC, Boni G, Barogui Y, Sopoh GE, Houndonougbo M, Anagonou E, et al. Assessment of water, sanitation, and hygiene practices and associated factors in a Buruli ulcer endemic district in Benin (West Africa). BMC Public Health. 2015;15:801; doi: 10.1186/s12889-015-2154-y. https://www.ncbi.nlm.nih.gov/pubmed/26286582.

627. Johansen MV, Lier T, Sithithaworn P. Towards improved diagnosis of neglected zoonotic trematodes using a One Health approach. Acta Trop. 2015;141 Pt B:161-9; doi: 10.1016/j.actatropica.2013.07.006. https://www.ncbi.nlm.nih.gov/pubmed/23886849.

628. Jain K, Jain NK. Vaccines for visceral leishmaniasis: A review. J Immunol Methods. 2015;422:1-12; doi: 10.1016/j.jim.2015.03.017. https://www.ncbi.nlm.nih.gov/pubmed/25858230.

629. Hotez PJ, Bottazzi ME, Strych U, Chang LY, Lim YA, Goodenow MM, et al. Neglected tropical diseases among the Association of Southeast Asian Nations (ASEAN): overview and update. PLoS Negl Trop Dis. 2015;9 4:e0003575; doi: 10.1371/journal.pntd.0003575. https://www.ncbi.nlm.nih.gov/pubmed/25880767.

630. Horstick O, Tozan Y, Wilder-Smith A. Reviewing dengue: still a neglected tropical disease? PLoS Negl Trop Dis. 2015;9 4:e0003632; doi: 10.1371/journal.pntd.0003632. https://www.ncbi.nlm.nih.gov/pubmed/25928673.

631. Hollingsworth TD, Pulliam JR, Funk S, Truscott JE, Isham V, Lloyd AL. Seven challenges for modelling indirect transmission: vector-borne diseases, macroparasites and neglected tropical diseases. Epidemics. 2015;10:16-20; doi: 10.1016/j.epidem.2014.08.007. https://www.ncbi.nlm.nih.gov/pubmed/25843376.

632. Hollingsworth TD, Langley I, Nokes DJ, Macpherson EE, McGivern G, Adams ER, et al. Infectious disease and health systems modelling for local decision making to control neglected tropical diseases. BMC Proc. 2015;9 Suppl 10:S6; doi: 10.1186/1753-6561-9-S10-S6. https://www.ncbi.nlm.nih.gov/pubmed/28281704.

633. Hollingsworth TD, Adams ER, Anderson RM, Atkins K, Bartsch S, Basanez MG, et al. Quantitative analyses and modelling to support achievement of the 2020 goals for nine neglected tropical diseases. Parasit Vectors. 2015;8:630; doi: 10.1186/s13071-015-1235-1. https://www.ncbi.nlm.nih.gov/pubmed/26652272.

634. Habib AG, Kuznik A, Hamza M, Abdullahi MI, Chedi BA, Chippaux JP, et al. Snakebite is Under Appreciated: Appraisal of Burden from West Africa. PLoS Negl Trop Dis. 2015;9 9:e0004088; doi: 10.1371/journal.pntd.0004088. https://www.ncbi.nlm.nih.gov/pubmed/26398046.

635. Gurarie D, Yoon N, Li E, Ndeffo-Mbah M, Durham D, Phillips AE, et al. Modelling control of Schistosoma haematobium infection: predictions of the long-term impact of mass drug administration in Africa. Parasit Vectors. 2015;8:529; doi: 10.1186/s13071-015-1144-3. https://www.ncbi.nlm.nih.gov/pubmed/26489408.

636. Gulland A. Progress on eliminating neglected tropical diseases is too slow, report warns. BMJ. 2015;350:h3465; doi: 10.1136/bmj.h3465. https://www.ncbi.nlm.nih.gov/pubmed/26112027.

637. Gordon CA, McManus DP, Acosta LP, Olveda RM, Williams GM, Ross AG, et al. Multiplex real-time PCR monitoring of intestinal helminths in humans reveals widespread polyparasitism in Northern Samar, the Philippines. Int J Parasitol. 2015;45 7:477-83; doi: 10.1016/j.ijpara.2015.02.011. https://www.ncbi.nlm.nih.gov/pubmed/25858090.

638. Geiger A, Ponton F, Simo G. Adult blood-feeding tsetse flies, trypanosomes, microbiota and the fluctuating environment in sub-Saharan Africa. ISME J. 2015;9 7:1496-507; doi: 10.1038/ismej.2014.236. https://www.ncbi.nlm.nih.gov/pubmed/25500509.

639. Gambhir M, Singh BK, Michael E. The Allee effect and elimination of neglected tropical diseases: a mathematical modelling study. Adv Parasitol. 2015;87:1-31; doi: 10.1016/bs.apar.2014.12.001. https://www.ncbi.nlm.nih.gov/pubmed/25765192.

640. Flores-Lopez CA, Machado CA. Differences in inferred genome-wide signals of positive selection during the evolution of Trypanosoma cruzi and Leishmania spp. lineages: A result of disparities in host and tissue infection ranges? Infect Genet Evol. 2015;33:37-46; doi: 10.1016/j.meegid.2015.04.008. https://www.ncbi.nlm.nih.gov/pubmed/25891283.

641. Finkelman J. Innovative community-based ecosystem management for dengue and Chagas disease prevention in low and middle income countries in Latin America and the Caribbean. Trans R Soc Trop Med Hyg. 2015;109 2:89-90; doi: 10.1093/trstmh/tru201. https://www.ncbi.nlm.nih.gov/pubmed/25604758.

642. El-Bahnasawy MM, Morsy AT, Morsy TA. The Arthropod-Borne Onchocerciasis: Is It Deserved to Be Neglected? J Egypt Soc Parasitol. 2015;45 3:639-54; doi: 10.12816/0017932. https://www.ncbi.nlm.nih.gov/pubmed/26939243.

643. Edwards J, Brown M, Peak E, Bartholomew B, Nash RJ, Hoffmann KF. The diterpenoid 7-keto-sempervirol, derived from Lycium chinense, displays anthelmintic activity against both Schistosoma mansoni and Fasciola hepatica. PLoS Negl Trop Dis. 2015;9 3:e0003604; doi: 10.1371/journal.pntd.0003604. https://www.ncbi.nlm.nih.gov/pubmed/25768432.

644. Dunn C, Callahan K, Katabarwa M, Richards F, Hopkins D, Withers PC, Jr., et al. The Contributions of Onchocerciasis Control and Elimination Programs toward the Achievement of the Millennium Development Goals. PLoS Negl Trop Dis. 2015;9 5:e0003703; doi: 10.1371/journal.pntd.0003703. https://www.ncbi.nlm.nih.gov/pubmed/25996946.

645. Duedu KO, Karikari YA, Attah SK, Ayeh-Kumi PF. Prevalence of intestinal parasites among patients of a Ghanaian psychiatry hospital. BMC Res Notes. 2015;8:651; doi: 10.1186/s13104-015-1634-6. https://www.ncbi.nlm.nih.gov/pubmed/26541287.

646. Ding W, Zhou XN, Guan YY, Yao JW, Xiao N. [Gaps on China's Engagement in Global Governance of Neglected Tropical Diseases]. Zhongguo Ji Sheng Chong Xue Yu Ji Sheng Chong Bing Za Zhi. 2015;33 6:407-11. https://www.ncbi.nlm.nih.gov/pubmed/27089768.

647. Deribe K, Wanji S, Shafi O, E MT, Umulisa I, Molyneux DH, et al. The feasibility of eliminating podoconiosis. Bull World Health Organ. 2015;93 10:712-8; doi: 10.2471/BLT.14.150276. https://www.ncbi.nlm.nih.gov/pubmed/26600613.

648. Deribe K. Neglected tropical disease targets must include morbidity. Lancet Glob Health. 2015;3 10:e596; doi: 10.1016/S2214-109X(15)00185-0. https://www.ncbi.nlm.nih.gov/pubmed/26385299.

649. de Morais CG, Castro Lima AK, Terra R, dos Santos RF, Da-Silva SA, Dutra PM. The Dialogue of the Host-Parasite Relationship: Leishmania spp. and Trypanosoma cruzi Infection. Biomed Res Int. 2015;2015:324915; doi: 10.1155/2015/324915. https://www.ncbi.nlm.nih.gov/pubmed/26090399.

650. Datta S, Roy S, Manna M. Therapy with radio-attenuated vaccine in experimental murine visceral leishmaniasis showed enhanced T cell and inducible nitric oxide synthase levels, suppressed tumor growth factor-beta production with higher expression of some signaling molecules. Braz J Infect Dis. 2015;19 1:36-42; doi: 10.1016/j.bjid.2014.10.009. https://www.ncbi.nlm.nih.gov/pubmed/25532783.

651. da Veiga CP, da Veiga CR, Del Corso JM, da Silva WV. Dengue Vaccines: A Perspective from the Point of View of Intellectual Property. Int J Environ Res Public Health. 2015;12 8:9454-74; doi: 10.3390/ijerph120809454. https://www.ncbi.nlm.nih.gov/pubmed/26274968.

652. Currie BJ. Scabies and Global Control of Neglected Tropical Diseases. N Engl J Med. 2015;373 24:2371-2; doi: 10.1056/NEJMe1511805. https://www.ncbi.nlm.nih.gov/pubmed/26650157.

653. Cortes LA, Castro L, Pesce B, Maya JD, Ferreira J, Castro-Castillo V, et al. Novel Gallate Triphenylphosphonium Derivatives with Potent Antichagasic Activity. PLoS One. 2015;10 8:e0136852; doi: 10.1371/journal.pone.0136852. https://www.ncbi.nlm.nih.gov/pubmed/26317199.

654. Coelho EA, Chavez-Fumagalli MA, Costa LE, Tavares CA, Soto M, Goulart LR. Theranostic applications of phage display to control leishmaniasis: selection of biomarkers for serodiagnostics, vaccination, and immunotherapy. Rev Soc Bras Med Trop. 2015;48 4:370-9; doi: 10.1590/0037-8682-0096-2015. https://www.ncbi.nlm.nih.gov/pubmed/26312925.

655. Choi MH, Yu JR, Hong ST. Who Neglects Neglected Tropical Diseases? - Korean Perspective. J Korean Med Sci. 2015;30 Suppl 2:S122-30; doi: 10.3346/jkms.2015.30.S2.S122. https://www.ncbi.nlm.nih.gov/pubmed/26617444.

656. Chikeka I, Dumler JS. Neglected bacterial zoonoses. Clin Microbiol Infect. 2015;21 5:404-15; doi: 10.1016/j.cmi.2015.04.022. https://www.ncbi.nlm.nih.gov/pubmed/25964152.

657. Cartelle Gestal M, Holban AM, Escalante S, Cevallos M. Epidemiology of Tropical Neglected Diseases in Ecuador in the Last 20 Years. PLoS One. 2015;10 9:e0138311; doi: 10.1371/journal.pone.0138311. https://www.ncbi.nlm.nih.gov/pubmed/26394405.

658. Brooker SJ, Nikolay B, Balabanova D, Pullan RL. Global feasibility assessment of interrupting the transmission of soil-transmitted helminths: a statistical modelling study. Lancet Infect Dis. 2015;15 8:941-50; doi: 10.1016/S1473-3099(15)70042-3. https://www.ncbi.nlm.nih.gov/pubmed/25886799.

659. Brooker SJ, Mwandawiro CS, Halliday KE, Njenga SM, McHaro C, Gichuki PM, et al. Interrupting transmission of soil-transmitted helminths: a study protocol for cluster randomised trials evaluating alternative treatment strategies and delivery systems in Kenya. BMJ Open. 2015;5 10:e008950; doi: 10.1136/bmjopen-2015-008950. https://www.ncbi.nlm.nih.gov/pubmed/26482774.

660. Bottazzi ME. The human hookworm vaccine: recent updates and prospects for success. J Helminthol. 2015;89 5:540-4; doi: 10.1017/S0022149X15000206. https://www.ncbi.nlm.nih.gov/pubmed/25850789.

661. Blood-Siegfried J, Zeantoe GC, Evans LJ, Bondo J, Forstner JR, Wood K. The Impact of Nurses on Neglected Tropical Disease Management. Public Health Nurs. 2015;32 6:680-701; doi: 10.1111/phn.12149. https://www.ncbi.nlm.nih.gov/pubmed/25229995.

662. Bergquist R, Yang GJ, Knopp S, Utzinger J, Tanner M. Surveillance and response: Tools and approaches for the elimination stage of neglected tropical diseases. Acta Trop. 2015;141 Pt B:229-34; doi: 10.1016/j.actatropica.2014.09.017. https://www.ncbi.nlm.nih.gov/pubmed/25301340.

663. Belizario VY, Jr., Totanes FI, de Leon WU, Ciro RN, Lumampao YF. Sentinel surveillance of soil-transmitted helminthiasis in preschool-aged and school-aged children in selected local government units in the Philippines: follow-up assessment. Asia Pac J Public Health. 2015;27 2:NP1604-15; doi: 10.1177/1010539513483825. https://www.ncbi.nlm.nih.gov/pubmed/23572379.

664. Basanez MG, Anderson RM. Mathematical Models for Neglected Tropical Diseases: Essential Tools for Control and Elimination, Part A. Adv Parasitol. 2015;87:xiii-xvii; doi: 10.1016/s0065-308x(15)00023-8. https://www.ncbi.nlm.nih.gov/pubmed/25924239.

665. Barbosa JF, de Figueiredo SM, Monteiro FM, Rocha-Silva F, Gaciele-Melo C, Coelho SS, et al. New Approaches on Leishmaniasis Treatment and Prevention: A Review of Recent Patents. Recent Pat Endocr Metab Immune Drug Discov. 2015;9 2:90-102; doi: 10.2174/1872214809666150921111956. https://www.ncbi.nlm.nih.gov/pubmed/26392062.

666. Bailey TC, Merritt MW, Tediosi F. Investing in justice: ethics, evidence, and the eradication investment cases for lymphatic filariasis and onchocerciasis. Am J Public Health. 2015;105 4:629-36; doi: 10.2105/AJPH.2014.302454. https://www.ncbi.nlm.nih.gov/pubmed/25713967.

667. Ashok P, Lathiya H, Murugesan S. Manzamine alkaloids as antileishmanial agents: A review. Eur J Med Chem. 2015;97:928-36; doi: 10.1016/j.ejmech.2014.07.006. https://www.ncbi.nlm.nih.gov/pubmed/25023608.

668. Armah FA, Quansah R, Luginaah I, Chuenpagdee R, Hambati H, Campbell G. Historical Perspective and Risk of Multiple Neglected Tropical Diseases in Coastal Tanzania: Compositional and Contextual Determinants of Disease Risk. PLoS Negl Trop Dis. 2015;9 8:e0003939; doi: 10.1371/journal.pntd.0003939. https://www.ncbi.nlm.nih.gov/pubmed/26241050.

669. Alhassan A, Li Z, Poole CB, Carlow CK. Expanding the MDx toolbox for filarial diagnosis and surveillance. Trends Parasitol. 2015;31 8:391-400; doi: 10.1016/j.pt.2015.04.006. https://www.ncbi.nlm.nih.gov/pubmed/25978936.

670. Albonico M, Levecke B, LoVerde PT, Montresor A, Prichard R, Vercruysse J, et al. Monitoring the efficacy of drugs for neglected tropical diseases controlled by preventive chemotherapy. J Glob Antimicrob Resist. 2015;3 4:229-36; doi: 10.1016/j.jgar.2015.08.004. https://www.ncbi.nlm.nih.gov/pubmed/27842865.

671. Adenowo AF, Oyinloye BE, Ogunyinka BI, Kappo AP. Impact of human schistosomiasis in sub-Saharan Africa. Braz J Infect Dis. 2015;19 2:196-205; doi: 10.1016/j.bjid.2014.11.004. https://www.ncbi.nlm.nih.gov/pubmed/25636189.

672. Addiss DG. The challenge of unreported and unprogrammed deworming for soil-transmitted helminth control programs. Int Health. 2015;7 6:377-9; doi: 10.1093/inthealth/ihv055. https://www.ncbi.nlm.nih.gov/pubmed/26311691.

673. Abou-El-Naga IF. Demographic, socioeconomic and environmental changes affecting circulation of neglected tropical diseases in Egypt. Asian Pac J Trop Med. 2015;8 11:881-8; doi: 10.1016/j.apjtm.2015.10.015. https://www.ncbi.nlm.nih.gov/pubmed/26614986.

674. Comprehensive Guide for Health Promotion and Empowerment Published. Ten Steps Training Guide Addresses Issues Common to Many Neglected Tropical Diseases. Lepr Rev. 2015;86 3:300. https://www.ncbi.nlm.nih.gov/pubmed/26665370.

675. Yang GJ, Liu L, Zhu HR, Griffiths SM, Tanner M, Bergquist R, et al. China's sustained drive to eliminate neglected tropical diseases. Lancet Infect Dis. 2014;14 9:881-92; doi: 10.1016/S1473-3099(14)70727-3. https://www.ncbi.nlm.nih.gov/pubmed/24875936.

676. Wouters OJ, Downs PW, Zoerhoff KL, Crowley KR, Frawley H, Einberg J, et al. Resource planning for neglected tropical disease (NTD) control programs: feasibility study of the Tool for Integrated Planning and Costing (TIPAC). PLoS Negl Trop Dis. 2014;8 2:e2619; doi: 10.1371/journal.pntd.0002619. https://www.ncbi.nlm.nih.gov/pubmed/24587453.

677. Woodhall DM, Mkwanda S, Dembele M, Lwanga H, Drexler N, Dubray C, et al. Exploring innovative ways to conduct coverage surveys for neglected tropical diseases in Malawi, Mali, and Uganda. Acta Trop. 2014;132:119-24; doi: 10.1016/j.actatropica.2014.01.001. https://www.ncbi.nlm.nih.gov/pubmed/24462795.

678. Wilson S, Dunne DW. A pressing need for parasite immunologists to contribute to our understanding of human-neglected tropical diseases. Parasite Immunol. 2014;36 8:325-7; doi: 10.1111/pim.12136. https://www.ncbi.nlm.nih.gov/pubmed/25196455.

679. Webster JP, Molyneux DH, Hotez PJ, Fenwick A. The contribution of mass drug administration to global health: past, present and future. Philos Trans R Soc Lond B Biol Sci. 2014;369 1645:20130434; doi: 10.1098/rstb.2013.0434. https://www.ncbi.nlm.nih.gov/pubmed/24821920.

680. Visser BJ. How soil scientists help combat podoconiosis, a neglected tropical disease. Int J Environ Res Public Health. 2014;11 5:5133-6; doi: 10.3390/ijerph110505133. https://www.ncbi.nlm.nih.gov/pubmed/24828083.

681. Vera-Polania F, Perilla-Gonzalez Y, Martinez-Pulgarin DF, Baquero-Rodriguez JD, Munoz-Urbano M, Lagos-Gallego M, et al. Bibliometric assessment of the Latin-American contributions in dengue. Recent Pat Antiinfect Drug Discov. 2014;9 3:195-201; doi: 10.2174/1574891x10666150410153224. https://www.ncbi.nlm.nih.gov/pubmed/25858260.

682. Vandersmissen A, Welburn SC. Current initiatives in One Health: consolidating the One Health Global Network. Rev Sci Tech. 2014;33 2:421-32; doi: 10.20506/rst.33.2.2297. https://www.ncbi.nlm.nih.gov/pubmed/25707173.

683. Turner HC, Walker M, French MD, Blake IM, Churcher TS, Basanez MG. Neglected tools for neglected diseases: mathematical models in economic evaluations. Trends Parasitol. 2014;30 12:562-70; doi: 10.1016/j.pt.2014.10.001. https://www.ncbi.nlm.nih.gov/pubmed/25455565.

684. Turner HC, Walker M, Churcher TS, Osei-Atweneboana MY, Biritwum NK, Hopkins A, et al. Reaching the london declaration on neglected tropical diseases goals for onchocerciasis: an economic evaluation of increasing the frequency of ivermectin treatment in Africa. Clin Infect Dis. 2014;59 7:923-32; doi: 10.1093/cid/ciu467. https://www.ncbi.nlm.nih.gov/pubmed/24944228.

685. Turner HC, Walker M, Churcher TS, Basanez MG. Modelling the impact of ivermectin on River Blindness and its burden of morbidity and mortality in African Savannah: EpiOncho projections. Parasit Vectors. 2014;7:241; doi: 10.1186/1756-3305-7-241. https://www.ncbi.nlm.nih.gov/pubmed/24886747.

686. Truscott J, Hollingsworth TD, Anderson R. Modeling the interruption of the transmission of soil-transmitted helminths by repeated mass chemotherapy of school-age children. PLoS Negl Trop Dis. 2014;8 12:e3323; doi: 10.1371/journal.pntd.0003323. https://www.ncbi.nlm.nih.gov/pubmed/25474477.

687. Tomczyk S, Deribe K, Brooker SJ, Clark H, Rafique K, Knopp S, et al. Association between footwear use and neglected tropical diseases: a systematic review and meta-analysis. PLoS Negl Trop Dis. 2014;8 11:e3285; doi: 10.1371/journal.pntd.0003285. https://www.ncbi.nlm.nih.gov/pubmed/25393620.

688. Thao NP, No JH, Luyen BT, Yang G, Byun SY, Goo J, et al. Secondary metabolites from Vietnamese marine invertebrates with activity against Trypanosoma brucei and T. cruzi. Molecules. 2014;19 6:7869-80; doi: 10.3390/molecules19067869. https://www.ncbi.nlm.nih.gov/pubmed/24962391.

689. Tambo E, Ai L, Zhou X, Chen JH, Hu W, Bergquist R, et al. Surveillance-response systems: the key to elimination of tropical diseases. Infect Dis Poverty. 2014;3:17; doi: 10.1186/2049-9957-3-17. https://www.ncbi.nlm.nih.gov/pubmed/24971165.

690. Stromme EM, Baeroe K, Norheim OF. Disease control priorities for neglected tropical diseases: lessons from priority ranking based on the quality of evidence, cost effectiveness, severity of disease, catastrophic health expenditures, and loss of productivity. Dev World Bioeth. 2014;14 3:132-41; doi: 10.1111/dewb.12016. https://www.ncbi.nlm.nih.gov/pubmed/23724925.

691. Sime H, Deribe K, Assefa A, Newport MJ, Enquselassie F, Gebretsadik A, et al. Integrated mapping of lymphatic filariasis and podoconiosis: lessons learnt from Ethiopia. Parasit Vectors. 2014;7:397; doi: 10.1186/1756-3305-7-397. https://www.ncbi.nlm.nih.gov/pubmed/25164687.

692. Sesay S, Paye J, Bah MS, McCarthy FM, Conteh A, Sonnie M, et al. Schistosoma mansoni infection after three years of mass drug administration in Sierra Leone. Parasit Vectors. 2014;7:14; doi: 10.1186/1756-3305-7-14. https://www.ncbi.nlm.nih.gov/pubmed/24401567.

693. Sergiev VP, Migliorini L, Litvinov SK, Chernikova EA. [Neglected tropical diseases: a New World Health Organization program]. Med Parazitol (Mosk). 2014; 2:59-63. https://www.ncbi.nlm.nih.gov/pubmed/25296432.

694. Seid A, Gadisa E, Tsegaw T, Abera A, Teshome A, Mulugeta A, et al. Risk map for cutaneous leishmaniasis in Ethiopia based on environmental factors as revealed by geographical information systems and statistics. Geospat Health. 2014;8 2:377-87; doi: 10.4081/gh.2014.27. https://www.ncbi.nlm.nih.gov/pubmed/24893015.

695. Salam RA, Maredia H, Das JK, Lassi ZS, Bhutta ZA. Community-based interventions for the prevention and control of helmintic neglected tropical diseases. Infect Dis Poverty. 2014;3:23; doi: 10.1186/2049-9957-3-23. https://www.ncbi.nlm.nih.gov/pubmed/25114793.

696. Sacks DL. Vaccines against tropical parasitic diseases: a persisting answer to a persisting problem. Nat Immunol. 2014;15 5:403-5; doi: 10.1038/ni.2853. https://www.ncbi.nlm.nih.gov/pubmed/24747701.

697. Rabito MF, Britta EA, Pelegrini BL, Scariot DB, Almeida MB, Nixdorf SL, et al. In vitro and in vivo antileishmania activity of sesquiterpene lactone-rich dichloromethane fraction obtained from Tanacetum parthenium (L.) Schultz-Bip. Exp Parasitol. 2014;143:18-23; doi: 10.1016/j.exppara.2014.04.014. https://www.ncbi.nlm.nih.gov/pubmed/24810433.

698. Qian YL, Wang W, Hong QB, Liang YS. [Bibliometric analysis of literature regarding integrated schistosomiasis control strategy with emphasis on infectious source control]. Zhongguo Xue Xi Chong Bing Fang Zhi Za Zhi. 2014;26 6:626-31. https://www.ncbi.nlm.nih.gov/pubmed/25856887.

699. Qian MB, Chen YD, Zhou XN. [From recognition to practice: The 140th anniversary of the discovery of Clonorchis sinensis]. Zhongguo Ji Sheng Chong Xue Yu Ji Sheng Chong Bing Za Zhi. 2014;32 4:247-52. https://www.ncbi.nlm.nih.gov/pubmed/25518585.

700. Perilla-Gonzalez Y, Gomez-Suta D, Delgado-Osorio N, Hurtado-Hurtado N, Baquero-Rodriguez JD, Lopez-Isaza AF, et al. Study of the scientific production on leishmaniasis in Latin America. Recent Pat Antiinfect Drug Discov. 2014;9 3:216-22; doi: 10.2174/1574891x10666150410165445. https://www.ncbi.nlm.nih.gov/pubmed/25858270.

701. Perbandt M, Ndjonka D, Liebau E. Protective mechanisms of helminths against reactive oxygen species are highly promising drug targets. Curr Med Chem. 2014;21 15:1794-808; doi: 10.2174/0929867320666131119122907. https://www.ncbi.nlm.nih.gov/pubmed/24251574.

702. Peng D, Kurup SP, Yao PY, Minning TA, Tarleton RL. CRISPR-Cas9-mediated single-gene and gene family disruption in Trypanosoma cruzi. mBio. 2014;6 1:e02097-14; doi: 10.1128/mBio.02097-14. https://www.ncbi.nlm.nih.gov/pubmed/25550322.

703. Peeling RW, Mabey D. Diagnostics for the control and elimination of neglected tropical diseases. Parasitology. 2014;141 14:1789-94; doi: 10.1017/S0031182014000973. https://www.ncbi.nlm.nih.gov/pubmed/25248096.

704. Paty MC. [Dengue fever in mainland France]. Arch Pediatr. 2014;21 11:1274-8; doi: 10.1016/j.arcped.2014.07.002. https://www.ncbi.nlm.nih.gov/pubmed/25080833.

705. Parker M, Allen T. De-politicizing parasites: reflections on attempts to control the control of neglected tropical diseases. Med Anthropol. 2014;33 3:223-39; doi: 10.1080/01459740.2013.831414. https://www.ncbi.nlm.nih.gov/pubmed/24761976.

706. Oyinloye B, Adenowo F, Gxaba N, Kappo A. The promise of antimicrobial peptides for treatment of human schistosomiasis. Curr Drug Targets. 2014;15 9:852-9; doi: 10.2174/1389450115666140807154810. https://www.ncbi.nlm.nih.gov/pubmed/25101908.

707. Olamiju OJ, Olamiju FO, Adeniran AA, Mba IC, Ukwunna CC, Okoronkwo C, et al. Public awareness and knowledge of neglected tropical diseases (NTDs) control activities in Abuja, Nigeria. PLoS Negl Trop Dis. 2014;8 9:e3209; doi: 10.1371/journal.pntd.0003209. https://www.ncbi.nlm.nih.gov/pubmed/25254362.

708. Okorie PN, Bockarie MJ, Molyneux DH, Kelly-Hope LA. Neglected tropical diseases: a systematic evaluation of research capacity in Nigeria. PLoS Negl Trop Dis. 2014;8 8:e3078; doi: 10.1371/journal.pntd.0003078. https://www.ncbi.nlm.nih.gov/pubmed/25121582.

709. Nsadha Z, Thomas LF, Fevre EM, Nasinyama G, Ojok L, Waiswa C. Prevalence of porcine cysticercosis in the Lake Kyoga Basin, Uganda. BMC Vet Res. 2014;10:239; doi: 10.1186/s12917-014-0239-y. https://www.ncbi.nlm.nih.gov/pubmed/25286901.

710. Njenga SM, Mutungi FM, Wamae CN, Mwanje MT, Njiru KK, Bockarie MJ. Once a year school-based deworming with praziquantel and albendazole combination may not be adequate for control of urogenital schistosomiasis and hookworm infection in Matuga District, Kwale County, Kenya. Parasit Vectors. 2014;7:74; doi: 10.1186/1756-3305-7-74. https://www.ncbi.nlm.nih.gov/pubmed/24552246.

711. Njelesani J, Dacombe R, Palmer T, Smith H, Koudou B, Bockarie M, et al. A systematic approach to capacity strengthening of laboratory systems for control of neglected tropical diseases in Ghana, Kenya, Malawi and Sri Lanka. PLoS Negl Trop Dis. 2014;8 3:e2736; doi: 10.1371/journal.pntd.0002736. https://www.ncbi.nlm.nih.gov/pubmed/24603407.

712. Nissen S, Nguyen LA, Thamsborg SM, Dalsgaard A, Johansen MV. Reinfection of dogs with fish-borne zoonotic trematodes in northern Vietnam following a single treatment with praziquantel. PLoS Negl Trop Dis. 2014;8 1:e2625; doi: 10.1371/journal.pntd.0002625. https://www.ncbi.nlm.nih.gov/pubmed/24392175.

713. Nie G, Wang T, Lu S, Liu W, Li Y, Lei J. Detection of Clonorchis sinensis circulating antigen in sera from Chinese patients by immunomagnetic bead ELISA based on IgY. PLoS One. 2014;9 12:e113208; doi: 10.1371/journal.pone.0113208. https://www.ncbi.nlm.nih.gov/pubmed/25474577.

714. Nguku P, Oyemakinde A, Sabitu K, Olayinka A, Ajayi I, Fawole O, et al. Training and service in public health, Nigeria Field Epidemiology and Laboratory Training, 2008 - 2014. Pan Afr Med J. 2014;18 Suppl 1:2; doi: 10.11694/pamj.supp.2014.18.1.4930. https://www.ncbi.nlm.nih.gov/pubmed/25328621.

715. Nelson R. Neglected tropical diseases take hold in the USA. Lancet Infect Dis. 2014;14 11:1050-1; doi: 10.1016/S1473-3099(14)70795-9. https://www.ncbi.nlm.nih.gov/pubmed/25541050.

716. Ndayishimiye O, Ortu G, Soares Magalhaes RJ, Clements A, Willems J, Whitton J, et al. Control of neglected tropical diseases in Burundi: partnerships, achievements, challenges, and lessons learned after four years of programme implementation. PLoS Negl Trop Dis. 2014;8 5:e2684; doi: 10.1371/journal.pntd.0002684. https://www.ncbi.nlm.nih.gov/pubmed/24785993.

717. Mugono M, Konje E, Kuhn S, Mpogoro FJ, Morona D, Mazigo HD. Intestinal schistosomiasis and geohelminths of Ukara Island, North-Western Tanzania: prevalence, intensity of infection and associated risk factors among school children. Parasit Vectors. 2014;7:612; doi: 10.1186/s13071-014-0612-5. https://www.ncbi.nlm.nih.gov/pubmed/25533267.

718. Molyneux DH. Neglected tropical diseases: now more than just 'other diseases'--the post-2015 agenda. Int Health. 2014;6 3:172-80; doi: 10.1093/inthealth/ihu037. https://www.ncbi.nlm.nih.gov/pubmed/24969646.

719. Midzi N, Mduluza T, Chimbari MJ, Tshuma C, Charimari L, Mhlanga G, et al. Distribution of schistosomiasis and soil transmitted helminthiasis in Zimbabwe: towards a national plan of action for control and elimination. PLoS Negl Trop Dis. 2014;8 8:e3014; doi: 10.1371/journal.pntd.0003014. https://www.ncbi.nlm.nih.gov/pubmed/25121489.

720. Metzger WG, Mordmuller B. Loa loa-does it deserve to be neglected? Lancet Infect Dis. 2014;14 4:353-7; doi: 10.1016/S1473-3099(13)70263-9. https://www.ncbi.nlm.nih.gov/pubmed/24332895.

721. McCarty TR, Turkeltaub JA, Hotez PJ. Global progress towards eliminating gastrointestinal helminth infections. Curr Opin Gastroenterol. 2014;30 1:18-24; doi: 10.1097/MOG.0000000000000025. https://www.ncbi.nlm.nih.gov/pubmed/24241244.

722. Mbang-Benet DE, Sterkers Y, Morelle C, Kebe NM, Crobu L, Portales P, et al. The bacterial-like HslVU protease complex subunits are involved in the control of different cell cycle events in trypanosomatids. Acta Trop. 2014;131:22-31; doi: 10.1016/j.actatropica.2013.11.017. https://www.ncbi.nlm.nih.gov/pubmed/24299926.

723. Maurice J. Neglected tropical diseases. Oral antibiotic raises hopes of eradicating yaws. Science. 2014;344 6180:142; doi: 10.1126/science.344.6180.142. https://www.ncbi.nlm.nih.gov/pubmed/24723591.

724. Masiga DK, Igweta L, Saini R, Ochieng'-Odero JP, Borgemeister C. Building endogenous capacity for the management of neglected tropical diseases in Africa: the pioneering role of ICIPE. PLoS Negl Trop Dis. 2014;8 5:e2687; doi: 10.1371/journal.pntd.0002687. https://www.ncbi.nlm.nih.gov/pubmed/24830708.

725. Madon S, Amaguru JO, Malecela MN, Michael E. Can mobile phones help control neglected tropical diseases? Experiences from Tanzania. Soc Sci Med. 2014;102:103-10; doi: 10.1016/j.socscimed.2013.11.036. https://www.ncbi.nlm.nih.gov/pubmed/24565147.

726. Mackey TK, Liang BA, Cuomo R, Hafen R, Brouwer KC, Lee DE. Emerging and reemerging neglected tropical diseases: a review of key characteristics, risk factors, and the policy and innovation environment. Clin Microbiol Rev. 2014;27 4:949-79; doi: 10.1128/CMR.00045-14. https://www.ncbi.nlm.nih.gov/pubmed/25278579.

727. Mableson HE, Okello A, Picozzi K, Welburn SC. Neglected zoonotic diseases-the long and winding road to advocacy. PLoS Negl Trop Dis. 2014;8 6:e2800; doi: 10.1371/journal.pntd.0002800. https://www.ncbi.nlm.nih.gov/pubmed/24901769.

728. Liese BH, Houghton N, Teplitskaya L. Development assistance for neglected tropical diseases: progress since 2009. Int Health. 2014;6 3:162-71; doi: 10.1093/inthealth/ihu052. https://www.ncbi.nlm.nih.gov/pubmed/25096331.

729. Liang S, Yang C, Zhong B, Guo J, Li H, Carlton EJ, et al. Surveillance systems for neglected tropical diseases: global lessons from China's evolving schistosomiasis reporting systems, 1949-2014. Emerg Themes Epidemiol. 2014;11:19; doi: 10.1186/1742-7622-11-19. https://www.ncbi.nlm.nih.gov/pubmed/26265928.

730. Leta S, Dao TH, Mesele F, Alemayehu G. Visceral leishmaniasis in Ethiopia: an evolving disease. PLoS Negl Trop Dis. 2014;8 9:e3131; doi: 10.1371/journal.pntd.0003131. https://www.ncbi.nlm.nih.gov/pubmed/25188253.

731. Lee SC, Ngui R, Tan TK, Muhammad Aidil R, Lim YA. Neglected tropical diseases among two indigenous subtribes in peninsular Malaysia: highlighting differences and co-infection of helminthiasis and sarcocystosis. PLoS One. 2014;9 9:e107980; doi: 10.1371/journal.pone.0107980. https://www.ncbi.nlm.nih.gov/pubmed/25248116.

732. Knight JM, Zingales B, Bottazzi ME, Hotez P, Zhan B. Limited antigenic variation in the Trypanosoma cruzi candidate vaccine antigen TSA-1. Parasite Immunol. 2014;36 12:708-12; doi: 10.1111/pim.12130. https://www.ncbi.nlm.nih.gov/pubmed/25040249.

733. Khan MB, Sonaimuthu P, Lau YL, Al-Mekhlafi HM, Mahmud R, Kavana N, et al. High seroprevalence of echinococossis, schistosomiasis and toxoplasmosis among the populations in Babati and Monduli districts, Tanzania. Parasit Vectors. 2014;7:505; doi: 10.1186/s13071-014-0505-7. https://www.ncbi.nlm.nih.gov/pubmed/25388913.

734. Keating J, Yukich JO, Mollenkopf S, Tediosi F. Lymphatic filariasis and onchocerciasis prevention, treatment, and control costs across diverse settings: a systematic review. Acta Trop. 2014;135:86-95; doi: 10.1016/j.actatropica.2014.03.017. https://www.ncbi.nlm.nih.gov/pubmed/24699086.

735. Kappagoda S, Ioannidis JP. Prevention and control of neglected tropical diseases: overview of randomized trials, systematic reviews and meta-analyses. Bull World Health Organ. 2014;92 5:356-66C; doi: 10.2471/BLT.13.129601. https://www.ncbi.nlm.nih.gov/pubmed/24839325.

736. Kabatereine N, Fleming F, Thuo W, Tinkitina B, Tukahebwa EM, Fenwick A. Community perceptions, attitude, practices and treatment seeking behaviour for schistosomiasis in L. Victoria islands in Uganda. BMC Res Notes. 2014;7:900; doi: 10.1186/1756-0500-7-900. https://www.ncbi.nlm.nih.gov/pubmed/25495121.

737. Jones AH, Becknell S, Withers PC, Ruiz-Tiben E, Hopkins DR, Stobbelaar D, et al. Logistics of Guinea worm disease eradication in South Sudan. Am J Trop Med Hyg. 2014;90 3:393-401; doi: 10.4269/ajtmh.13-0110. https://www.ncbi.nlm.nih.gov/pubmed/24445199.

738. Johnston KL, Ford L, Taylor MJ. Overcoming the challenges of drug discovery for neglected tropical diseases: the A.WOL experience. J Biomol Screen. 2014;19 3:335-43; doi: 10.1177/1087057113511270. https://www.ncbi.nlm.nih.gov/pubmed/24241712.

739. Jadhav S, Gautam M, Gairola S. Role of vaccine manufacturers in developing countries towards global healthcare by providing quality vaccines at affordable prices. Clin Microbiol Infect. 2014;20 Suppl 5:37-44; doi: 10.1111/1469-0691.12568. https://www.ncbi.nlm.nih.gov/pubmed/24476201.

740. Jackson Y, Stephenson N. Neglected tropical disease and emerging infectious disease: an analysis of the history, promise and constraints of two worldviews. Glob Public Health. 2014;9 9:995-1007; doi: 10.1080/17441692.2014.941297. https://www.ncbi.nlm.nih.gov/pubmed/25096397.

741. Ito A, Wandra T, Li T, Dekumyoy P, Nkouawa A, Okamoto M, et al. The present situation of human taeniases and cysticercosis in Asia. Recent Pat Antiinfect Drug Discov. 2014;9 3:173-85; doi: 10.2174/1574891x10666150410125711. https://www.ncbi.nlm.nih.gov/pubmed/25858304.

742. Hotez PJ, Velasquez RM, Wolf JE, Jr. Neglected tropical skin diseases: their global elimination through integrated mass drug administration? JAMA Dermatol. 2014;150 5:481-2; doi: 10.1001/jamadermatol.2013.8759. https://www.ncbi.nlm.nih.gov/pubmed/24671756.

743. Hotez PJ. Could nitazoxanide be added to other essential medicines for integrated neglected tropical disease control and elimination? PLoS Negl Trop Dis. 2014;8 3:e2758; doi: 10.1371/journal.pntd.0002758. https://www.ncbi.nlm.nih.gov/pubmed/24675990.

744. Hotez PJ. Global christianity and the control of its neglected tropical diseases. PLoS Negl Trop Dis. 2014;8 11:e3135; doi: 10.1371/journal.pntd.0003135. https://www.ncbi.nlm.nih.gov/pubmed/25412254.

745. Hotez PJ. The medical biochemistry of poverty and neglect. Mol Med. 2014;20 Suppl 1:S31-6; doi: 10.2119/molmed.2014.00169. https://www.ncbi.nlm.nih.gov/pubmed/25549231.

746. Holmes P, Strategic WHO, Advisory Group on Neglected Tropical D. Neglected tropical diseases in the post-2015 health agenda. Lancet. 2014;383 9931:1803; doi: 10.1016/S0140-6736(14)60875-8. https://www.ncbi.nlm.nih.gov/pubmed/24856022.

747. Hagen J, Young ND, Every AL, Pagel CN, Schnoeller C, Scheerlinck JP, et al. Omega-1 knockdown in Schistosoma mansoni eggs by lentivirus transduction reduces granuloma size in vivo. Nat Commun. 2014;5:5375; doi: 10.1038/ncomms6375. https://www.ncbi.nlm.nih.gov/pubmed/25400038.

748. Gurunath U, Joshi R, Agrawal A, Shah V. An overview of visceral leishmaniasis elimination program in India: a picture imperfect. Expert Rev Anti Infect Ther. 2014;12 8:929-35; doi: 10.1586/14787210.2014.928590. https://www.ncbi.nlm.nih.gov/pubmed/24930676.

749. Gungor K, Hotez PJ, Ozdemir V, Aynacioglu S. Glaucomics: a call for systems diagnostics for 21(st) century ophthalmology and personalized visual health. OMICS. 2014;18 5:275-9; doi: 10.1089/omi.2014.0040. https://www.ncbi.nlm.nih.gov/pubmed/24730382.

750. Gulland A. One of world's poorest countries makes "remarkable" progress on tropical diseases, report says. BMJ. 2014;348:g3752; doi: 10.1136/bmj.g3752. https://www.ncbi.nlm.nih.gov/pubmed/24903135.

751. Greenberg RM. Ion channels and drug transporters as targets for anthelmintics. Curr Clin Microbiol Rep. 2014;1 3-4:51-60; doi: 10.1007/s40588-014-0007-6. https://www.ncbi.nlm.nih.gov/pubmed/25554739.

752. Gebrekristos HT, Buekens P. Mother-to-Child Transmission of Trypanosoma cruzi. J Pediatric Infect Dis Soc. 2014;3 Suppl 1:S36-40; doi: 10.1093/jpids/piu059. https://www.ncbi.nlm.nih.gov/pubmed/25232476.

753. Gaze S, Driguez P, Pearson MS, Mendes T, Doolan DL, Trieu A, et al. An immunomics approach to schistosome antigen discovery: antibody signatures of naturally resistant and chronically infected individuals from endemic areas. PLoS Pathog. 2014;10 3:e1004033; doi: 10.1371/journal.ppat.1004033. https://www.ncbi.nlm.nih.gov/pubmed/24675823.

754. Gabrie JA, Rueda MM, Canales M, Gyorkos TW, Sanchez AL. School hygiene and deworming are key protective factors for reduced transmission of soil-transmitted helminths among schoolchildren in Honduras. Parasit Vectors. 2014;7:354; doi: 10.1186/1756-3305-7-354. https://www.ncbi.nlm.nih.gov/pubmed/25091035.

755. Furst T, Ouattara M, Silue KD, N'Goran DN, Adiossan LG, Bogoch, II, et al. Scope and Limits of an anamnestic questionnaire in a control-induced low-endemicity helminthiasis setting in south-central Cote d'Ivoire. PLoS One. 2014;8 6:e64380; doi: 10.1371/journal.pone.0064380. https://www.ncbi.nlm.nih.gov/pubmed/23755120.

756. Fujii Y, Kaneko S, Nzou SM, Mwau M, Njenga SM, Tanigawa C, et al. Serological surveillance development for tropical infectious diseases using simultaneous microsphere-based multiplex assays and finite mixture models. PLoS Negl Trop Dis. 2014;8 7:e3040; doi: 10.1371/journal.pntd.0003040. https://www.ncbi.nlm.nih.gov/pubmed/25078404.

757. Edwards T, Allen E, Harding-Esch EM, Hart J, Burr SE, Holland MJ, et al. Non-participation during azithromycin mass treatment for trachoma in The Gambia: heterogeneity and risk factors. PLoS Negl Trop Dis. 2014;8 8:e3098; doi: 10.1371/journal.pntd.0003098. https://www.ncbi.nlm.nih.gov/pubmed/25165994.

758. Dhimal M, Karki KB. The hidden burden of Neglected Tropical Diseases: a call for inter-sectoral collaboration in Nepal. J Nepal Health Res Counc. 2014;12 28:I-IV. https://www.ncbi.nlm.nih.gov/pubmed/26032064.

759. Del Brutto OH. Neurocysticercosis. Handb Clin Neurol. 2014;121:1445-59; doi: 10.1016/B978-0-7020-4088-7.00097-3. https://www.ncbi.nlm.nih.gov/pubmed/24365429.

760. Dang K, Tribble AC. Strategies in infectious disease prevention and management among US-bound refugee children. Curr Probl Pediatr Adolesc Health Care. 2014;44 7:196-207; doi: 10.1016/j.cppeds.2014.03.004. https://www.ncbi.nlm.nih.gov/pubmed/25042432.

761. Chen JH, Zhang T, Ju C, Xu B, Lu Y, Mo XJ, et al. An integrated immunoproteomics and bioinformatics approach for the analysis of Schistosoma japonicum tegument proteins. J Proteomics. 2014;98:289-99; doi: 10.1016/j.jprot.2014.01.010. https://www.ncbi.nlm.nih.gov/pubmed/24448400.

762. Chammartin F, Guimaraes LH, Scholte RG, Bavia ME, Utzinger J, Vounatsou P. Spatio-temporal distribution of soil-transmitted helminth infections in Brazil. Parasit Vectors. 2014;7:440; doi: 10.1186/1756-3305-7-440. https://www.ncbi.nlm.nih.gov/pubmed/25230810.

763. Cashwell A, Tantri A, Schmidt A, Simon G, Mistry N. BRICS in the response to neglected tropical diseases. Bull World Health Organ. 2014;92 6:461-2; doi: 10.2471/BLT.13.132555. https://www.ncbi.nlm.nih.gov/pubmed/24940024.

764. Cano J, Rebollo MP, Golding N, Pullan RL, Crellen T, Soler A, et al. The global distribution and transmission limits of lymphatic filariasis: past and present. Parasit Vectors. 2014;7:466; doi: 10.1186/s13071-014-0466-x. https://www.ncbi.nlm.nih.gov/pubmed/25303991.

765. Calvopina M, Romero D, Castaneda B, Hashiguchi Y, Sugiyama H. Current status of Paragonimus and paragonimiasis in Ecuador. Mem Inst Oswaldo Cruz. 2014;109 7:849-55; doi: 10.1590/0074-0276140042. https://www.ncbi.nlm.nih.gov/pubmed/25410987.

766. Bottazzi ME. Vaccines against neglected tropical diseases: promising interventions to rescue the poorest populations in the Americas. Immunotherapy. 2014;6 2:117-9; doi: 10.2217/imt.13.159. https://www.ncbi.nlm.nih.gov/pubmed/24491084.

767. Botelho-Junior S, Machado OL, Fernandes KV, Lemos FJ, Perdizio VA, Oliveira AE, et al. Defense response in non-genomic model species: methyl jasmonate exposure reveals the passion fruit leaves' ability to assemble a cocktail of functionally diversified Kunitz-type trypsin inhibitors and recruit two of them against papain. Planta. 2014;240 2:345-56; doi: 10.1007/s00425-014-2085-3. https://www.ncbi.nlm.nih.gov/pubmed/24849173.

768. Bobes RJ, Fragoso G, Fleury A, Garcia-Varela M, Sciutto E, Larralde C, et al. Evolution, molecular epidemiology and perspectives on the research of taeniid parasites with special emphasis on Taenia solium. Infect Genet Evol. 2014;23:150-60; doi: 10.1016/j.meegid.2014.02.005. https://www.ncbi.nlm.nih.gov/pubmed/24560729.

769. Bhutta ZA, Sommerfeld J, Lassi ZS, Salam RA, Das JK. Global burden, distribution, and interventions for infectious diseases of poverty. Infect Dis Poverty. 2014;3:21; doi: 10.1186/2049-9957-3-21. https://www.ncbi.nlm.nih.gov/pubmed/25110585.

770. Bhutta ZA, Salam RA, Das JK, Lassi ZS. Tackling the existing burden of infectious diseases in the developing world: existing gaps and the way forward. Infect Dis Poverty. 2014;3:28; doi: 10.1186/2049-9957-3-28. https://www.ncbi.nlm.nih.gov/pubmed/25105015.

771. Bhattacharya S, Chakraborty M, Mukhopadhyay P, Kundu PP, Mishra R. Viper and cobra venom neutralization by alginate coated multicomponent polyvalent antivenom administered by the oral route. PLoS Negl Trop Dis. 2014;8 8:e3039; doi: 10.1371/journal.pntd.0003039. https://www.ncbi.nlm.nih.gov/pubmed/25102172.

772. Bhakat S, Karubiu W, Jayaprakash V, Soliman ME. A perspective on targeting non-structural proteins to combat neglected tropical diseases: Dengue, West Nile and Chikungunya viruses. Eur J Med Chem. 2014;87:677-702; doi: 10.1016/j.ejmech.2014.10.010. https://www.ncbi.nlm.nih.gov/pubmed/25305334.

773. Betson M, Nejsum P, Bendall RP, Deb RM, Stothard JR. Molecular epidemiology of ascariasis: a global perspective on the transmission dynamics of Ascaris in people and pigs. J Infect Dis. 2014;210 6:932-41; doi: 10.1093/infdis/jiu193. https://www.ncbi.nlm.nih.gov/pubmed/24688073.

774. Bardosh K, Inthavong P, Xayaheuang S, Okello AL. Controlling parasites, understanding practices: the biosocial complexity of a One Health intervention for neglected zoonotic helminths in northern Lao PDR. Soc Sci Med. 2014;120:215-23; doi: 10.1016/j.socscimed.2014.09.030. https://www.ncbi.nlm.nih.gov/pubmed/25261615.

775. Bardosh K. Global aspirations, local realities: the role of social science research in controlling neglected tropical diseases. Infect Dis Poverty. 2014;3 1:35; doi: 10.1186/2049-9957-3-35. https://www.ncbi.nlm.nih.gov/pubmed/25320672.

776. Babu BV, Babu GR. Coverage of, and compliance with, mass drug administration under the programme to eliminate lymphatic filariasis in India: a systematic review. Trans R Soc Trop Med Hyg. 2014;108 9:538-49; doi: 10.1093/trstmh/tru057. https://www.ncbi.nlm.nih.gov/pubmed/24728444.

777. Asiedu K, Fitzpatrick C, Jannin J. Eradication of yaws: historical efforts and achieving WHO's 2020 target. PLoS Negl Trop Dis. 2014;8 9:e3016; doi: 10.1371/journal.pntd.0003016. https://www.ncbi.nlm.nih.gov/pubmed/25254372.

778. Arnold BF, Priest JW, Hamlin KL, Moss DM, Colford JM, Jr., Lammie PJ. Serological measures of malaria transmission in Haiti: comparison of longitudinal and cross-sectional methods. PLoS One. 2014;9 4:e93684; doi: 10.1371/journal.pone.0093684. https://www.ncbi.nlm.nih.gov/pubmed/24691467.

779. Neglected tropical diseases: becoming less neglected. Lancet. 2014;383 9925:1269; doi: 10.1016/S0140-6736(14)60629-2. https://www.ncbi.nlm.nih.gov/pubmed/24725560.

780. Tackling neglected diseases. Vet Rec. 2014;175 6:138; doi: 10.1136/vr.g5043. https://www.ncbi.nlm.nih.gov/pubmed/25103739.

781. Zhou XN, Bergquist R, Tanner M. Elimination of tropical disease through surveillance and response. Infect Dis Poverty. 2013;2 1:1; doi: 10.1186/2049-9957-2-1. https://www.ncbi.nlm.nih.gov/pubmed/23849433.

782. Yao JW, Zhou XN. [To overcome neglected tropical diseases by global health governance]. Zhongguo Xue Xi Chong Bing Fang Zhi Za Zhi. 2013;25 2:190-3. https://www.ncbi.nlm.nih.gov/pubmed/23894844.

783. Xu XL, Zhu R, Zhang LJ, Lu LT, Guo JG. [Information analysis of development of researches on global neglected tropical diseases]. Zhongguo Xue Xi Chong Bing Fang Zhi Za Zhi. 2013;25 2:160-6. https://www.ncbi.nlm.nih.gov/pubmed/23894837.

784. World Health O. WHO Expert Consultation on Rabies. Second report. World Health Organ Tech Rep Ser. 2013; 982:1-139, back cover. https://www.ncbi.nlm.nih.gov/pubmed/24069724.

785. World Health O. Control and surveillance of human African trypanosomiasis. World Health Organ Tech Rep Ser. 2013; 984:1-237. https://www.ncbi.nlm.nih.gov/pubmed/24552089.

786. Wells S, Diap G, Kiechel JR. The story of artesunate-mefloquine (ASMQ), innovative partnerships in drug development: case study. Malar J. 2013;12:68; doi: 10.1186/1475-2875-12-68. https://www.ncbi.nlm.nih.gov/pubmed/23433060.

787. Warrell DA, Gutierrez JM, Calvete JJ, Williams D. New approaches & technologies of venomics to meet the challenge of human envenoming by snakebites in India. Indian J Med Res. 2013;138:38-59. https://www.ncbi.nlm.nih.gov/pubmed/24056555.

788. Wanyua S, Ndemwa M, Goto K, Tanaka J, K'Opiyo J, Okumu S, et al. Profile: the Mbita health and demographic surveillance system. Int J Epidemiol. 2013;42 6:1678-85; doi: 10.1093/ije/dyt180. https://www.ncbi.nlm.nih.gov/pubmed/24415606.

789. Vreysen MJ, Seck MT, Sall B, Bouyer J. Tsetse flies: their biology and control using area-wide integrated pest management approaches. J Invertebr Pathol. 2013;112 Suppl:S15-25; doi: 10.1016/j.jip.2012.07.026. https://www.ncbi.nlm.nih.gov/pubmed/22878217.

790. Vouking MZ, Tamo VC, Mbuagbaw L. The impact of community health workers (CHWs) on Buruli ulcer in sub-Saharan Africa: a systematic review. Pan Afr Med J. 2013;15:19; doi: 10.11604/pamj.2013.15.19.1991. https://www.ncbi.nlm.nih.gov/pubmed/24009795.

791. Vanlerberghe V, Verdonck K. [Inequities in health: the case of dengue]. Rev Peru Med Exp Salud Publica. 2013;30 4:683-6. https://www.ncbi.nlm.nih.gov/pubmed/24448949.

792. Spero D, Levitz L, De Groot AS. Report from the field: Overview of the Sixth Annual Vaccine Renaissance Conference. Hum Vaccin Immunother. 2013;9 7:1555-7; doi: 10.4161/hv.24833. https://www.ncbi.nlm.nih.gov/pubmed/23732897.

793. Spear RC. Commentary by Spear, R. on "Integration of water, sanitation, and hygiene for the prevention and control of neglected tropical diseases: a rationale for inter-sectoral collaboration:" can the control of NTDs profit from a good WASH? PLoS Negl Trop Dis. 2013;7 9:e2473; doi: 10.1371/journal.pntd.0002473. https://www.ncbi.nlm.nih.gov/pubmed/24086791.

794. Soares Magalhaes RJ, Langa A, Pedro JM, Sousa-Figueiredo JC, Clements AC, Vaz Nery S. Role of malnutrition and parasite infections in the spatial variation in children's anaemia risk in northern Angola. Geospat Health. 2013;7 2:341-54; doi: 10.4081/gh.2013.91. https://www.ncbi.nlm.nih.gov/pubmed/23733295.

795. Smith J, Taylor EM. MDGs and NTDs: reshaping the global health agenda. PLoS Negl Trop Dis. 2013;7 12:e2529; doi: 10.1371/journal.pntd.0002529. https://www.ncbi.nlm.nih.gov/pubmed/24349587.

796. Slater H, Michael E. Mapping, bayesian geostatistical analysis and spatial prediction of lymphatic filariasis prevalence in Africa. PLoS One. 2013;8 8:e71574; doi: 10.1371/journal.pone.0071574. https://www.ncbi.nlm.nih.gov/pubmed/23951194.

797. Simarro PP, Franco JR, Diarra A, Ruiz Postigo JA, Jannin J. Diversity of human African trypanosomiasis epidemiological settings requires fine-tuning control strategies to facilitate disease elimination. Res Rep Trop Med. 2013;4:1-6; doi: 10.2147/RRTM.S40157. https://www.ncbi.nlm.nih.gov/pubmed/30100778.

798. Schur N, Hurlimann E, Stensgaard AS, Chimfwembe K, Mushinge G, Simoonga C, et al. Spatially explicit Schistosoma infection risk in eastern Africa using Bayesian geostatistical modelling. Acta Trop. 2013;128 2:365-77; doi: 10.1016/j.actatropica.2011.10.006. https://www.ncbi.nlm.nih.gov/pubmed/22019933.

799. Schar F, Trostdorf U, Giardina F, Khieu V, Muth S, Marti H, et al. Strongyloides stercoralis: Global Distribution and Risk Factors. PLoS Negl Trop Dis. 2013;7 7:e2288; doi: 10.1371/journal.pntd.0002288. https://www.ncbi.nlm.nih.gov/pubmed/23875033.

800. Sady H, Al-Mekhlafi HM, Mahdy MA, Lim YA, Mahmud R, Surin J. Prevalence and associated factors of Schistosomiasis among children in Yemen: implications for an effective control programme. PLoS Negl Trop Dis. 2013;7 8:e2377; doi: 10.1371/journal.pntd.0002377. https://www.ncbi.nlm.nih.gov/pubmed/23991235.

801. Ross AG, Olveda RM, Acosta L, Harn DA, Chy D, Li Y, et al. Road to the elimination of schistosomiasis from Asia: the journey is far from over. Microbes Infect. 2013;15 13:858-65; doi: 10.1016/j.micinf.2013.07.010. https://www.ncbi.nlm.nih.gov/pubmed/23973709.

802. Rilkoff H, Tukahebwa EM, Fleming FM, Leslie J, Cole DC. Exploring gender dimensions of treatment programmes for neglected tropical diseases in Uganda. PLoS Negl Trop Dis. 2013;7 7:e2312; doi: 10.1371/journal.pntd.0002312. https://www.ncbi.nlm.nih.gov/pubmed/23875047.

803. Raoul F, Li T, Sako Y, Chen X, Long C, Yanagida T, et al. Advances in diagnosis and spatial analysis of cysticercosis and taeniasis. Parasitology. 2013;140 13:1578-88; doi: 10.1017/S0031182013001303. https://www.ncbi.nlm.nih.gov/pubmed/23985371.

804. Qian YJ, Li SZ, Wang Q, Zhang L, Liu W, Chen JX, et al. [Rapid risk assessment on the import of American trypanosomiasis to China]. Zhongguo Ji Sheng Chong Xue Yu Ji Sheng Chong Bing Za Zhi. 2013;31 1:57-9. https://www.ncbi.nlm.nih.gov/pubmed/24812840.

805. Qian MB, Chen YD, Zhou XN. [Research priorities for the control and elimination of major helminthiases]. Zhongguo Ji Sheng Chong Xue Yu Ji Sheng Chong Bing Za Zhi. 2013;31 2:155-9. https://www.ncbi.nlm.nih.gov/pubmed/24809202.

806. Pedrique B, Strub-Wourgaft N, Some C, Olliaro P, Trouiller P, Ford N, et al. The drug and vaccine landscape for neglected diseases (2000-11): a systematic assessment. Lancet Glob Health. 2013;1 6:e371-9; doi: 10.1016/S2214-109X(13)70078-0. https://www.ncbi.nlm.nih.gov/pubmed/25104602.

807. O'Neill HG, Mzilahowa T, de Deus N, Njenga SM, Mmbaga EJ, Kariuki TM. Evaluation of the European foundation initiative into African research in neglected tropical diseases by the African fellows. PLoS Negl Trop Dis. 2013;7 3:e2019; doi: 10.1371/journal.pntd.0002019. https://www.ncbi.nlm.nih.gov/pubmed/23516641.

808. Olds GR. Deworming the world. Trans Am Clin Climatol Assoc. 2013;124:265-74. https://www.ncbi.nlm.nih.gov/pubmed/23874034.

809. Noden BH, van der Colf BE. Neglected tropical diseases of Namibia: unsolved mysteries. Acta Trop. 2013;125 1:1-17; doi: 10.1016/j.actatropica.2012.09.007. https://www.ncbi.nlm.nih.gov/pubmed/23006744.

810. Noa Noatina B, Kagmeni G, Mengouo MN, Moungui HC, Tarini A, Zhang Y, et al. Prevalence of trachoma in the Far North region of Cameroon: results of a survey in 27 Health Districts. PLoS Negl Trop Dis. 2013;7 5:e2240; doi: 10.1371/journal.pntd.0002240. https://www.ncbi.nlm.nih.gov/pubmed/23717703.

811. Negussu N, Wali M, Ejigu M, Debebe F, Aden S, Abdi R, et al. Prevalence and distribution of schistosomiasis in afder and gode zone of somali region, ethiopia. J Glob Infect Dis. 2013;5 4:149-52; doi: 10.4103/0974-777X.122007. https://www.ncbi.nlm.nih.gov/pubmed/24672176.

812. Natuzzi E. Neglected tropical diseases: is it time to add Helicobacter pylori to the list? Glob Health Promot. 2013;20 3:47-8; doi: 10.1177/1757975913499037. https://www.ncbi.nlm.nih.gov/pubmed/23986381.

813. Narahari SR, Bose KS, Aggithaya MG, Swamy GK, Ryan TJ, Unnikrishnan B, et al. Community level morbidity control of lymphoedema using self care and integrative treatment in two lymphatic filariasis endemic districts of South India: a non randomized interventional study. Trans R Soc Trop Med Hyg. 2013;107 9:566-77; doi: 10.1093/trstmh/trt054. https://www.ncbi.nlm.nih.gov/pubmed/23832181.

814. Nagpal S, Sinclair D, Garner P. Has the NTD community neglected evidence-based policy? PLoS Negl Trop Dis. 2013;7 7:e2238; doi: 10.1371/journal.pntd.0002238. https://www.ncbi.nlm.nih.gov/pubmed/23875030.

815. Mwangoka G, Ogutu B, Msambichaka B, Mzee T, Salim N, Kafuruki S, et al. Experience and challenges from clinical trials with malaria vaccines in Africa. Malar J. 2013;12:86; doi: 10.1186/1475-2875-12-86. https://www.ncbi.nlm.nih.gov/pubmed/23496910.

816. Mwandawiro CS, Nikolay B, Kihara JH, Ozier O, Mukoko DA, Mwanje MT, et al. Monitoring and evaluating the impact of national school-based deworming in Kenya: study design and baseline results. Parasit Vectors. 2013;6:198; doi: 10.1186/1756-3305-6-198. https://www.ncbi.nlm.nih.gov/pubmed/23829767.

817. Mwanakasale V, Songolo P, Daka V. Challenges in the control of human African trypanosomiasis in the Mpika district of Zambia. BMC Res Notes. 2013;6:180; doi: 10.1186/1756-0500-6-180. https://www.ncbi.nlm.nih.gov/pubmed/23642032.

818. Mullan Z. The good, the bad, and the neglected. Lancet Glob Health. 2013;1 2:e55; doi: 10.1016/S2214-109X(13)70058-5. https://www.ncbi.nlm.nih.gov/pubmed/25104146.

819. Mkupasi EM, Sikasunge CS, Ngowi HA, Johansen MV. Efficacy and safety of anthelmintics tested against Taenia solium cysticercosis in pigs. PLoS Negl Trop Dis. 2013;7 7:e2200; doi: 10.1371/journal.pntd.0002200. https://www.ncbi.nlm.nih.gov/pubmed/23936558.

820. Lozano-Fuentes S, Wedyan F, Hernandez-Garcia E, Sadhu D, Ghosh S, Bieman JM, et al. Cell phone-based system (Chaak) for surveillance of immatures of dengue virus mosquito vectors. J Med Entomol. 2013;50 4:879-89; doi: 10.1603/me13008. https://www.ncbi.nlm.nih.gov/pubmed/23926788.

821. Linder E, Grote A, Varjo S, Linder N, Lebbad M, Lundin M, et al. On-chip imaging of Schistosoma haematobium eggs in urine for diagnosis by computer vision. PLoS Negl Trop Dis. 2013;7 12:e2547; doi: 10.1371/journal.pntd.0002547. https://www.ncbi.nlm.nih.gov/pubmed/24340107.

822. Leslie J, Garba A, Boubacar K, Yaye Y, Sebongou H, Barkire A, et al. Neglected tropical diseases: comparison of the costs of integrated and vertical preventive chemotherapy treatment in Niger. Int Health. 2013;5 1:78-84; doi: 10.1093/inthealth/ihs010. https://www.ncbi.nlm.nih.gov/pubmed/24029850.

823. Lejon V, Bentivoglio M, Franco JR. Human African trypanosomiasis. Handb Clin Neurol. 2013;114:169-81; doi: 10.1016/B978-0-444-53490-3.00011-X. https://www.ncbi.nlm.nih.gov/pubmed/23829907.

824. Lal V. The neglected. Ann Med Health Sci Res. 2013;3 2:255-7; doi: 10.4103/2141-9248.113671. https://www.ncbi.nlm.nih.gov/pubmed/23919199.

825. Kovacic V, Tirados I, Esterhuizen J, Mangwiro CT, Torr SJ, Lehane MJ, et al. Community acceptance of tsetse control baits: a qualitative study in Arua District, North West Uganda. PLoS Negl Trop Dis. 2013;7 12:e2579; doi: 10.1371/journal.pntd.0002579. https://www.ncbi.nlm.nih.gov/pubmed/24349593.

826. Knopp S, Becker SL, Ingram KJ, Keiser J, Utzinger J. Diagnosis and treatment of schistosomiasis in children in the era of intensified control. Expert Rev Anti Infect Ther. 2013;11 11:1237-58; doi: 10.1586/14787210.2013.844066. https://www.ncbi.nlm.nih.gov/pubmed/24127662.

827. Kline K, McCarthy JS, Pearson M, Loukas A, Hotez PJ. Neglected tropical diseases of Oceania: review of their prevalence, distribution, and opportunities for control. PLoS Negl Trop Dis. 2013;7 1:e1755; doi: 10.1371/journal.pntd.0001755. https://www.ncbi.nlm.nih.gov/pubmed/23383349.

828. King JD, Buolamwini J, Cromwell EA, Panfel A, Teferi T, Zerihun M, et al. A novel electronic data collection system for large-scale surveys of neglected tropical diseases. PLoS One. 2013;8 9:e74570; doi: 10.1371/journal.pone.0074570. https://www.ncbi.nlm.nih.gov/pubmed/24066147.

829. Keenan JD, Hotez PJ, Amza A, Stoller NE, Gaynor BD, Porco TC, et al. Elimination and eradication of neglected tropical diseases with mass drug administrations: a survey of experts. PLoS Negl Trop Dis. 2013;7 12:e2562; doi: 10.1371/journal.pntd.0002562. https://www.ncbi.nlm.nih.gov/pubmed/24340111.

830. Hotez PJ, Dumonteil E, Heffernan MJ, Bottazzi ME. Innovation for the 'bottom 100 million': eliminating neglected tropical diseases in the Americas. Adv Exp Med Biol. 2013;764:1-12; doi: 10.1007/978-1-4614-4726-9_1. https://www.ncbi.nlm.nih.gov/pubmed/23654053.

831. Hotez PJ, Diemert D, Bacon KM, Beaumier C, Bethony JM, Bottazzi ME, et al. The Human Hookworm Vaccine. Vaccine. 2013;31 Suppl 2:B227-32; doi: 10.1016/j.vaccine.2012.11.034. https://www.ncbi.nlm.nih.gov/pubmed/23598487.

832. Hotez PJ. NTDs V.2.0: "blue marble health"--neglected tropical disease control and elimination in a shifting health policy landscape. PLoS Negl Trop Dis. 2013;7 11:e2570; doi: 10.1371/journal.pntd.0002570. https://www.ncbi.nlm.nih.gov/pubmed/24278496.

833. Hotez P, Singh SK, Zhou XN. Advancing Sino-Indian cooperation to combat tropical diseases. PLoS Negl Trop Dis. 2013;7 9:e2204; doi: 10.1371/journal.pntd.0002204. https://www.ncbi.nlm.nih.gov/pubmed/24086775.

834. Hooper PJ, Zoerhoff KL, Kyelem D, Chu B, Flueckiger RM, Bamani S, et al. The effects of integration on financing and coverage of neglected tropical disease programs. Am J Trop Med Hyg. 2013;89 3:407-10; doi: 10.4269/ajtmh.13-0018. https://www.ncbi.nlm.nih.gov/pubmed/23836563.

835. Head MG, Fitchett JR, Cooke MK, Wurie FB, Hayward AC, Atun R. UK investments in global infectious disease research 1997-2010: a case study. Lancet Infect Dis. 2013;13 1:55-64; doi: 10.1016/S1473-3099(12)70261-X. https://www.ncbi.nlm.nih.gov/pubmed/23140942.

836. Gupta R, Wise PH. Leveraging information technology to improve control of neglected tropical diseases. PLoS Negl Trop Dis. 2013;7 11:e2353; doi: 10.1371/journal.pntd.0002353. https://www.ncbi.nlm.nih.gov/pubmed/24244759.

837. Guo JG, Xu XL, Zhu R. [Overcoming the global impact of neglected tropical diseases and challenges]. Zhongguo Xue Xi Chong Bing Fang Zhi Za Zhi. 2013;25 2:121-4. https://www.ncbi.nlm.nih.gov/pubmed/23894828.

838. Gulland A. Donated drugs boost protection against neglected tropical diseases. BMJ. 2013;346:f351; doi: 10.1136/bmj.f351. https://www.ncbi.nlm.nih.gov/pubmed/23325895.

839. Greenberg RM. New approaches for understanding mechanisms of drug resistance in schistosomes. Parasitology. 2013;140 12:1534-46; doi: 10.1017/S0031182013000231. https://www.ncbi.nlm.nih.gov/pubmed/23552512.

840. Gilbert IH. Drug discovery for neglected diseases: molecular target-based and phenotypic approaches. J Med Chem. 2013;56 20:7719-26; doi: 10.1021/jm400362b. https://www.ncbi.nlm.nih.gov/pubmed/24015767.

841. Gamboa-Angulo M, Molina-Salinas GM, Chan-Bacab M, Peraza-Sanchez SR, Heredia G, de la Rosa-Garcia SC, et al. Antimycobacterial and antileishmanial effects of microfungi isolated from tropical regions in Mexico. Parasitol Res. 2013;112 2:559-66; doi: 10.1007/s00436-012-3167-4. https://www.ncbi.nlm.nih.gov/pubmed/23086442.

842. Gabrielli AF, Montresor A, Nicholls RS, Ault SK. Progress towards the control and elimination of neglected tropical diseases in Brazil. J Pediatr (Rio J). 2013;89 3:215-6; doi: 10.1016/j.jped.2013.03.017. https://www.ncbi.nlm.nih.gov/pubmed/23684452.

843. Freeman MC, Ogden S, Jacobson J, Abbott D, Addiss DG, Amnie AG, et al. Integration of water, sanitation, and hygiene for the prevention and control of neglected tropical diseases: a rationale for inter-sectoral collaboration. PLoS Negl Trop Dis. 2013;7 9:e2439; doi: 10.1371/journal.pntd.0002439. https://www.ncbi.nlm.nih.gov/pubmed/24086781.

844. Franklin H, Tora A, Deribe K, Reda AA, Davey G. Development of a scale to measure stigma related to podoconiosis in Southern Ethiopia. BMC Public Health. 2013;13:298; doi: 10.1186/1471-2458-13-298. https://www.ncbi.nlm.nih.gov/pubmed/23556435.

845. Fiuza JA, Santiago Hda C, Selvapandiyan A, Gannavaram S, Ricci ND, Bueno LL, et al. Induction of immunogenicity by live attenuated Leishmania donovani centrin deleted parasites in dogs. Vaccine. 2013;31 14:1785-92; doi: 10.1016/j.vaccine.2013.01.048. https://www.ncbi.nlm.nih.gov/pubmed/23398933.

846. Engelman D, Martin DL, Hay RJ, Chosidow O, McCarthy JS, Fuller LC, et al. Opportunities to investigate the effects of ivermectin mass drug administration on scabies. Parasit Vectors. 2013;6:106; doi: 10.1186/1756-3305-6-106. https://www.ncbi.nlm.nih.gov/pubmed/23594459.

847. Dye C, Mertens T, Hirnschall G, Mpanju-Shumbusho W, Newman RD, Raviglione MC, et al. WHO and the future of disease control programmes. Lancet. 2013;381 9864:413-8; doi: 10.1016/S0140-6736(12)61812-1. https://www.ncbi.nlm.nih.gov/pubmed/23374479.

848. Dabo A, Bary B, Kouriba B, Sankare O, Doumbo O. Factors associated with coverage of praziquantel for schistosomiasis control in the community-direct intervention (CDI) approach in Mali (West Africa). Infect Dis Poverty. 2013;2 1:11; doi: 10.1186/2049-9957-2-11. https://www.ncbi.nlm.nih.gov/pubmed/23849481.

849. Curti E, Kwityn C, Zhan B, Gillespie P, Brelsford J, Deumic V, et al. Expression at a 20L scale and purification of the extracellular domain of the Schistosoma mansoni TSP-2 recombinant protein: a vaccine candidate for human intestinal schistosomiasis. Hum Vaccin Immunother. 2013;9 11:2342-50; doi: 10.4161/hv.25787. https://www.ncbi.nlm.nih.gov/pubmed/23899507.

850. Coulibaly YI, Dicko I, Keita M, Keita MM, Doumbia M, Daou A, et al. A cluster randomized study of the safety of integrated treatment of trachoma and lymphatic filariasis in children and adults in Sikasso, Mali. PLoS Negl Trop Dis. 2013;7 5:e2221; doi: 10.1371/journal.pntd.0002221. https://www.ncbi.nlm.nih.gov/pubmed/23675549.

851. Costa DN, Codeco CT, Silva MA, Werneck GL. Culling dogs in scenarios of imperfect control: realistic impact on the prevalence of canine visceral leishmaniasis. PLoS Negl Trop Dis. 2013;7 8:e2355; doi: 10.1371/journal.pntd.0002355. https://www.ncbi.nlm.nih.gov/pubmed/23951375.

852. Chammartin F, Scholte RG, Guimaraes LH, Tanner M, Utzinger J, Vounatsou P. Soil-transmitted helminth infection in South America: a systematic review and geostatistical meta-analysis. Lancet Infect Dis. 2013;13 6:507-18; doi: 10.1016/S1473-3099(13)70071-9. https://www.ncbi.nlm.nih.gov/pubmed/23562238.

853. Chammartin F, Hurlimann E, Raso G, N'Goran EK, Utzinger J, Vounatsou P. Statistical methodological issues in mapping historical schistosomiasis survey data. Acta Trop. 2013;128 2:345-52; doi: 10.1016/j.actatropica.2013.04.012. https://www.ncbi.nlm.nih.gov/pubmed/23648217.

854. Chami GF, Molyneux DH, Kontoleon AA, Dunne DW. Exploring network theory for mass drug administration. Trends Parasitol. 2013;29 8:370-9; doi: 10.1016/j.pt.2013.04.005. https://www.ncbi.nlm.nih.gov/pubmed/23742966.

855. Carbajal-de-la-Fuente AL, Yadon ZE. A scientometric evaluation of the Chagas disease implementation research programme of the PAHO and TDR. PLoS Negl Trop Dis. 2013;7 11:e2445; doi: 10.1371/journal.pntd.0002445. https://www.ncbi.nlm.nih.gov/pubmed/24244761.

856. Carabarin-Lima A, Gonzalez-Vazquez MC, Rodriguez-Morales O, Baylon-Pacheco L, Rosales-Encina JL, Reyes-Lopez PA, et al. Chagas disease (American trypanosomiasis) in Mexico: an update. Acta Trop. 2013;127 2:126-35; doi: 10.1016/j.actatropica.2013.04.007. https://www.ncbi.nlm.nih.gov/pubmed/23643518.

857. Butera JA. Phenotypic screening as a strategic component of drug discovery programs targeting novel antiparasitic and antimycobacterial agents: an editorial. J Med Chem. 2013;56 20:7715-8; doi: 10.1021/jm400443k. https://www.ncbi.nlm.nih.gov/pubmed/23927625.

858. Bossard G, Cuny G, Geiger A. Secreted proteases of Trypanosoma brucei gambiense: possible targets for sleeping sickness control? Biofactors. 2013;39 4:407-14; doi: 10.1002/biof.1100. https://www.ncbi.nlm.nih.gov/pubmed/23553721.

859. Bockarie MJ, Kelly-Hope LA, Rebollo M, Molyneux DH. Preventive chemotherapy as a strategy for elimination of neglected tropical parasitic diseases: endgame challenges. Philos Trans R Soc Lond B Biol Sci. 2013;368 1623:20120144; doi: 10.1098/rstb.2012.0144. https://www.ncbi.nlm.nih.gov/pubmed/23798692.

860. Beaumier CM, Gillespie PM, Hotez PJ, Bottazzi ME. New vaccines for neglected parasitic diseases and dengue. Transl Res. 2013;162 3:144-55; doi: 10.1016/j.trsl.2013.03.006. https://www.ncbi.nlm.nih.gov/pubmed/23578479.

861. Barry MA, Simon GG, Mistry N, Hotez PJ. Global trends in neglected tropical disease control and elimination: impact on child health. Arch Dis Child. 2013;98 8:635-41; doi: 10.1136/archdischild-2012-302338. https://www.ncbi.nlm.nih.gov/pubmed/23793132.

862. Baker MC, Krotki K, Sankara DP, Trofimovich L, Zoerhoff KL, Courtney L, et al. Measuring treatment coverage for neglected tropical disease control programs: analysis of a survey design. Am J Epidemiol. 2013;178 2:268-75; doi: 10.1093/aje/kws468. https://www.ncbi.nlm.nih.gov/pubmed/23860563.

863. Bailey MS. A brief history of British military experiences with infectious and tropical diseases. J R Army Med Corps. 2013;159 3:150-7; doi: 10.1136/jramc-2013-000087. https://www.ncbi.nlm.nih.gov/pubmed/24109135.

864. Bacon KM, Hotez PJ, Kruchten SD, Kamhawi S, Bottazzi ME, Valenzuela JG, et al. The potential economic value of a cutaneous leishmaniasis vaccine in seven endemic countries in the Americas. Vaccine. 2013;31 3:480-6; doi: 10.1016/j.vaccine.2012.11.032. https://www.ncbi.nlm.nih.gov/pubmed/23176979.

865. Ayode D, McBride CM, de Heer HD, Watanabe E, Gebreyesus T, Tora A, et al. A qualitative study exploring barriers related to use of footwear in rural highland ethiopia: implications for neglected tropical disease control. PLoS Negl Trop Dis. 2013;7 4:e2199; doi: 10.1371/journal.pntd.0002199. https://www.ncbi.nlm.nih.gov/pubmed/23638211.

866. Anderson RM, Truscott JE, Pullan RL, Brooker SJ, Hollingsworth TD. How effective is school-based deworming for the community-wide control of soil-transmitted helminths? PLoS Negl Trop Dis. 2013;7 2:e2027; doi: 10.1371/journal.pntd.0002027. https://www.ncbi.nlm.nih.gov/pubmed/23469293.

867. Neglected tropical diseases: progress and priorities. Lancet. 2013;381 9863:268; doi: 10.1016/S0140-6736(13)60115-4. https://www.ncbi.nlm.nih.gov/pubmed/23351795.

868. Rolling out and scaling up integrated preventive chemotherapy for selected neglected tropical diseases. Wkly Epidemiol Rec. 2013;88 16:161-6. https://www.ncbi.nlm.nih.gov/pubmed/23620908.

869. Drug discovery for the treatment of leishmaniasis, African sleeping sickness and Chagas disease. Future Med Chem. 2013;5 15:1709-18; doi: 10.4155/fmc.13.161. https://www.ncbi.nlm.nih.gov/pubmed/24144408.

870. Zhou XN. Prioritizing research for "One health - One world". Infect Dis Poverty. 2012;1 1:1; doi: 10.1186/2049-9957-1-1. https://www.ncbi.nlm.nih.gov/pubmed/23849840.

871. Yajima A, Mikhailov A, Mbabazi PS, Gabrielli AF, Minchiotti S, Montresor A, et al. Preventive Chemotherapy and Transmission Control (PCT) databank: a tool for planning, implementation and monitoring of integrated preventive chemotherapy for control of neglected tropical diseases. Trans R Soc Trop Med Hyg. 2012;106 4:215-22; doi: 10.1016/j.trstmh.2012.01.003. https://www.ncbi.nlm.nih.gov/pubmed/22357399.

872. Xie Z, Nsofor I, Tolhurst R. [Evaluation on implementation of the African programme for onchocerciasis control in Nigeria]. Beijing Da Xue Xue Bao Yi Xue Ban. 2012;44 3:403-6. https://www.ncbi.nlm.nih.gov/pubmed/22692311.

873. Worrell C, Mathieu E. Drug coverage surveys for neglected tropical diseases: 10 years of field experience. Am J Trop Med Hyg. 2012;87 2:216-22; doi: 10.4269/ajtmh.2012.12-0167. https://www.ncbi.nlm.nih.gov/pubmed/22855750.

874. World Health O. Research priorities for zoonoses and marginalized infections. World Health Organ Tech Rep Ser. 2012; 971:ix-xi, 1-119, 2 p following https://www.ncbi.nlm.nih.gov/pubmed/23420951.

875. Welburn SC, Maudlin I. Priorities for the elimination of sleeping sickness. Adv Parasitol. 2012;79:299-337; doi: 10.1016/B978-0-12-398457-9.00004-4. https://www.ncbi.nlm.nih.gov/pubmed/22726645.

876. Wall LL. Obstetric fistula is a "neglected tropical disease". PLoS Negl Trop Dis. 2012;6 8:e1769; doi: 10.1371/journal.pntd.0001769. https://www.ncbi.nlm.nih.gov/pubmed/22953008.

877. van Griensven J. Neglected tropical diseases: operational research for elimination and control. Public Health Action. 2012;2 3:45-6; doi: 10.5588/pha.12.0054. https://www.ncbi.nlm.nih.gov/pubmed/26392949.

878. Utzinger J, Becker SL, Knopp S, Blum J, Neumayr AL, Keiser J, et al. Neglected tropical diseases: diagnosis, clinical management, treatment and control. Swiss Med Wkly. 2012;142:w13727; doi: 10.4414/smw.2012.13727. https://www.ncbi.nlm.nih.gov/pubmed/23180107.

879. Upadhyayula SM, Mutheneni SR, Kadiri MR, Kumaraswamy S, Nelaturu SC. Data base management system for lymphatic filariasis--a neglected tropical disease. PLoS One. 2012;7 7:e39970; doi: 10.1371/journal.pone.0039970. https://www.ncbi.nlm.nih.gov/pubmed/22792200.

880. Tomczyk S, Tamiru A, Davey G. Addressing the neglected tropical disease podoconiosis in Northern Ethiopia: lessons learned from a new community podoconiosis program. PLoS Negl Trop Dis. 2012;6 3:e1560; doi: 10.1371/journal.pntd.0001560. https://www.ncbi.nlm.nih.gov/pubmed/22428078.

881. Tekola Ayele F, Adeyemo A, Finan C, Hailu E, Sinnott P, Burlinson ND, et al. HLA class II locus and susceptibility to podoconiosis. N Engl J Med. 2012;366 13:1200-8; doi: 10.1056/NEJMoa1108448. https://www.ncbi.nlm.nih.gov/pubmed/22455414.

882. Stefanakis R, Robertson AS, Ponder EL, Moree M. Analysis of neglected tropical disease drug and vaccine development pipelines to predict issuance of FDA priority review vouchers over the next decade. PLoS Negl Trop Dis. 2012;6 10:e1803; doi: 10.1371/journal.pntd.0001803. https://www.ncbi.nlm.nih.gov/pubmed/23145186.

883. Ruxin J, Negin J. Removing the neglect from neglected tropical diseases: the Rwandan experience 2008-2010. Glob Public Health. 2012;7 8:812-22; doi: 10.1080/17441692.2012.699535. https://www.ncbi.nlm.nih.gov/pubmed/22812700.

884. Rosales-Mendoza S, Govea-Alonso DO, Monreal-Escalante E, Fragoso G, Sciutto E. Developing plant-based vaccines against neglected tropical diseases: where are we? Vaccine. 2012;31 1:40-8; doi: 10.1016/j.vaccine.2012.10.094. https://www.ncbi.nlm.nih.gov/pubmed/23142588.

885. Parks T, Smeesters PR, Steer AC. Streptococcal skin infection and rheumatic heart disease. Curr Opin Infect Dis. 2012;25 2:145-53; doi: 10.1097/QCO.0b013e3283511d27. https://www.ncbi.nlm.nih.gov/pubmed/22327467.

886. Osei-Atweneboana MY, Lustigman S, Prichard RK, Boatin BA, Basanez MG. A research agenda for helminth diseases of humans: health research and capacity building in disease-endemic countries for helminthiases control. PLoS Negl Trop Dis. 2012;6 4:e1602; doi: 10.1371/journal.pntd.0001602. https://www.ncbi.nlm.nih.gov/pubmed/22545167.

887. Oliveira CR, Rezende CM, Silva MR, Pego AP, Borges O, Goes AM. A new strategy based on SmRho protein loaded chitosan nanoparticles as a candidate oral vaccine against schistosomiasis. PLoS Negl Trop Dis. 2012;6 11:e1894; doi: 10.1371/journal.pntd.0001894. https://www.ncbi.nlm.nih.gov/pubmed/23209848.

888. Montresor A, Gabrielli AF, Chitsulo L, Ichimori K, Mariotti S, Engels D, et al. Preventive chemotherapy and the fight against neglected tropical diseases. Expert Rev Anti Infect Ther. 2012;10 2:237-42; doi: 10.1586/eri.11.165. https://www.ncbi.nlm.nih.gov/pubmed/22339196.

889. Molyneux DH. The 'Neglected Tropical Diseases': now a brand identity; responsibilities, context and promise. Parasit Vectors. 2012;5:23; doi: 10.1186/1756-3305-5-23. https://www.ncbi.nlm.nih.gov/pubmed/22289579.

890. Mensah GA, Mayosi BM. The 2011 United Nations high-level meeting on non-communicable diseases: the Africa agenda calls for a 5-by-5 approach. S Afr Med J. 2012;103 2:77-9; doi: 10.7196/samj.6347. https://www.ncbi.nlm.nih.gov/pubmed/23374298.

891. Malone JB, Bergquist NR. Mapping and modelling neglected tropical diseases and poverty in Latin America and the Caribbean. Geospat Health. 2012;6 3:S1-5; doi: 10.4081/gh.2012.115. https://www.ncbi.nlm.nih.gov/pubmed/23032274.

892. MacNeil A, Rollin PE. Ebola and Marburg hemorrhagic fevers: neglected tropical diseases? PLoS Negl Trop Dis. 2012;6 6:e1546; doi: 10.1371/journal.pntd.0001546. https://www.ncbi.nlm.nih.gov/pubmed/22761967.

893. Mackey TK, Liang BA. Global health policy coordination to address neglected tropical diseases. Trop Med Int Health. 2012;17 9:1053-6; doi: 10.1111/j.1365-3156.2012.03049.x. https://www.ncbi.nlm.nih.gov/pubmed/22845755.

894. Mackey TK, Liang BA. Threats from emerging and re-emerging neglected tropical diseases (NTDs). Infect Ecol Epidemiol. 2012;2; doi: 10.3402/iee.v2i0.18667. https://www.ncbi.nlm.nih.gov/pubmed/22957134.

895. Lindo JF. Chagas: a neglected tropical disease. West Indian Med J. 2012;61 6:557-8. https://www.ncbi.nlm.nih.gov/pubmed/23441346.

896. Lesshafft H, Schuster A, Reichert F, Talhari S, Ignatius R, Feldmeier H. Knowledge, attitudes, perceptions, and practices regarding cutaneous larva migrans in deprived communities in Manaus, Brazil. J Infect Dev Ctries. 2012;6 5:422-9; doi: 10.3855/jidc.2122. https://www.ncbi.nlm.nih.gov/pubmed/22610709.

897. Lemnge MM, Mmbando BP, Segeja MD, Gesase S, Bygbjerg IC. Impact of insecticide treated mosquito nets and low dose monthly diethylcarbamazine on lymphatic filariasis infection between 1999 and 2004 in two endemic communities of north-eastern Tanzania. Tanzan J Health Res. 2012;14 3:166-74; doi: 10.4314/thrb.v14i3.2. https://www.ncbi.nlm.nih.gov/pubmed/26591753.

898. Lee J. Committed to fight. International effort targets neglected tropical diseases. Mod Healthc. 2012;42 6:7, 16. https://www.ncbi.nlm.nih.gov/pubmed/22356080.

899. Lammie PJ, Moss DM, Brook Goodhew E, Hamlin K, Krolewiecki A, West SK, et al. Development of a new platform for neglected tropical disease surveillance. Int J Parasitol. 2012;42 9:797-800; doi: 10.1016/j.ijpara.2012.07.002. https://www.ncbi.nlm.nih.gov/pubmed/22846784.

900. Korevaar DA, Visser BJ. Podoconiosis, a neglected tropical disease. Neth J Med. 2012;70 5:210-4. https://www.ncbi.nlm.nih.gov/pubmed/22744921.

901. Khan MG, Bhaskar KR, Salam MA, Akther T, Pluschke G, Mondal D. Diagnostic accuracy of loop-mediated isothermal amplification (LAMP) for detection of Leishmania DNA in buffy coat from visceral leishmaniasis patients. Parasit Vectors. 2012;5:280; doi: 10.1186/1756-3305-5-280. https://www.ncbi.nlm.nih.gov/pubmed/23206441.

902. Iloh GU, Obikwu CE, Amadi AN. Common geriatric morbidity from communicable diseases in a rural hospital in Eastern Nigeria. Niger J Med. 2012;21 2:231-6. https://www.ncbi.nlm.nih.gov/pubmed/23311198.

903. Hotez PJ, Savioli L, Fenwick A. Neglected tropical diseases of the Middle East and North Africa: review of their prevalence, distribution, and opportunities for control. PLoS Negl Trop Dis. 2012;6 2:e1475; doi: 10.1371/journal.pntd.0001475. https://www.ncbi.nlm.nih.gov/pubmed/22389729.

904. Hotez PJ, Bottazzi ME, Dumonteil E, Valenzuela JG, Kamhawi S, Ortega J, et al. Texas and Mexico: sharing a legacy of poverty and neglected tropical diseases. PLoS Negl Trop Dis. 2012;6 3:e1497; doi: 10.1371/journal.pntd.0001497. https://www.ncbi.nlm.nih.gov/pubmed/22479656.

905. Hotez PJ, Asojo OA, Adesina AM. Nigeria: "Ground Zero" for the high prevalence neglected tropical diseases. PLoS Negl Trop Dis. 2012;6 7:e1600; doi: 10.1371/journal.pntd.0001600. https://www.ncbi.nlm.nih.gov/pubmed/22860138.

906. Hotez PJ. Fighting neglected tropical diseases in the southern United States. BMJ. 2012;345:e6112; doi: 10.1136/bmj.e6112. https://www.ncbi.nlm.nih.gov/pubmed/22977143.

907. Hotez PJ. Engaging a rising China through neglected tropical diseases. PLoS Negl Trop Dis. 2012;6 11:e1599; doi: 10.1371/journal.pntd.0001599. https://www.ncbi.nlm.nih.gov/pubmed/23209845.

908. Horstick O, Farrar J, Lum L, Martinez E, San Martin JL, Ehrenberg J, et al. Reviewing the development, evidence base, and application of the revised dengue case classification. Pathog Glob Health. 2012;106 2:94-101; doi: 10.1179/2047773212Y.0000000017. https://www.ncbi.nlm.nih.gov/pubmed/22943544.

909. Hopkins A. Beyond providing drugs: the Mectizan(R) donation stimulates new strategies in service delivery and in strengthening health systems. Curr Pharm Biotechnol. 2012;13 6:1110-9; doi: 10.2174/138920112800399220. https://www.ncbi.nlm.nih.gov/pubmed/22039801.

910. Holt F, Gillam SJ, Ngondi JM. Improving access to medicines for neglected tropical diseases in developing countries: lessons from three emerging economies. PLoS Negl Trop Dis. 2012;6 2:e1390; doi: 10.1371/journal.pntd.0001390. https://www.ncbi.nlm.nih.gov/pubmed/22389728.

911. Hodges MH, Dada N, Warmsley A, Paye J, Bangura MM, Nyorkor E, et al. Mass drug administration significantly reduces infection of Schistosoma mansoni and hookworm in school children in the national control program in Sierra Leone. BMC Infect Dis. 2012;12:16; doi: 10.1186/1471-2334-12-16. https://www.ncbi.nlm.nih.gov/pubmed/22264258.

912. Hanson C, Weaver A, Zoerhoff KL, Kabore A, Linehan M, Doherty A, et al. Integrated implementation of programs targeting neglected tropical diseases through preventive chemotherapy: identifying best practices to roll out programs at national scale. Am J Trop Med Hyg. 2012;86 3:508-13; doi: 10.4269/ajtmh.2012.11-1589. https://www.ncbi.nlm.nih.gov/pubmed/22403327.

913. Hampton T. Collaborative effort targets 17 tropical diseases for control, elimination. JAMA. 2012;307 8:772; doi: 10.1001/jama.2012.201. https://www.ncbi.nlm.nih.gov/pubmed/22357823.

914. Hall A, Zhang Y, Macarthur C, Baker S. The role of nutrition in integrated programs to control neglected tropical diseases. BMC Med. 2012;10:41; doi: 10.1186/1741-7015-10-41. https://www.ncbi.nlm.nih.gov/pubmed/22533927.

915. Gowen BB, Ennis J, Sefing EJ, Wong MH, Jung KH, Turner JD. Extended protection against phlebovirus infection conferred by recombinant adenovirus expressing consensus interferon (DEF201). Antimicrob Agents Chemother. 2012;56 8:4168-74; doi: 10.1128/AAC.00376-12. https://www.ncbi.nlm.nih.gov/pubmed/22615273.

916. Giri S, Parija SC. A review on diagnostic and preventive aspects of cystic echinococcosis and human cysticercosis. Trop Parasitol. 2012;2 2:99-108; doi: 10.4103/2229-5070.105174. https://www.ncbi.nlm.nih.gov/pubmed/23767016.

917. Foss NT, Motta AC. Leprosy, a neglected disease that causes a wide variety of clinical conditions in tropical countries. Mem Inst Oswaldo Cruz. 2012;107 Suppl 1:28-33; doi: 10.1590/s0074-02762012000900006. https://www.ncbi.nlm.nih.gov/pubmed/23283450.

918. Fenwick A. The global burden of neglected tropical diseases. Public Health. 2012;126 3:233-6; doi: 10.1016/j.puhe.2011.11.015. https://www.ncbi.nlm.nih.gov/pubmed/22325616.

919. Emerson P, Kollmann M, MacArthur C, Bush S, Haddad D. SAFE strategy for blinding trachoma addresses sanitation, the other half of MDG7. Lancet. 2012;380 9836:27-8; doi: 10.1016/S0140-6736(12)61122-2. https://www.ncbi.nlm.nih.gov/pubmed/22770455.

920. El-Moamly A, El-Sweify M, Hafeez M. Performance of rK39 immunochromatography and freeze-dried direct agglutination tests in the diagnosis of imported visceral leishmaniasis. Parasitol Res. 2012;110 1:349-54; doi: 10.1007/s00436-011-2499-9. https://www.ncbi.nlm.nih.gov/pubmed/21710347.

921. Dorlo TP, Ravinetto RM, Beijnen JH, Boelaert M. Commentary: Substandard medicines are the priority for neglected tropical diseases. BMJ. 2012;345:e7518; doi: 10.1136/bmj.e7518. https://www.ncbi.nlm.nih.gov/pubmed/23152570.

922. Dorkenoo AM, Bronzan RN, Ayena KD, Anthony G, Agbo YM, Sognikin KS, et al. Nationwide integrated mapping of three neglected tropical diseases in Togo: countrywide implementation of a novel approach. Trop Med Int Health. 2012;17 7:896-903; doi: 10.1111/j.1365-3156.2012.03004.x. https://www.ncbi.nlm.nih.gov/pubmed/22594642.

923. Deribe K, Meribo K, Gebre T, Hailu A, Ali A, Aseffa A, et al. The burden of neglected tropical diseases in Ethiopia, and opportunities for integrated control and elimination. Parasit Vectors. 2012;5:240; doi: 10.1186/1756-3305-5-240. https://www.ncbi.nlm.nih.gov/pubmed/23095679.

924. Dembele M, Bamani S, Dembele R, Traore MO, Goita S, Traore MN, et al. Implementing preventive chemotherapy through an integrated National Neglected Tropical Disease Control Program in Mali. PLoS Negl Trop Dis. 2012;6 3:e1574; doi: 10.1371/journal.pntd.0001574. https://www.ncbi.nlm.nih.gov/pubmed/22448294.

925. Davey G, Bockarie M, Wanji S, Addiss D, Fuller C, Fox L, et al. Launch of the international podoconiosis initiative. Lancet. 2012;379 9820:1004; doi: 10.1016/S0140-6736(12)60427-9. https://www.ncbi.nlm.nih.gov/pubmed/22423883.

926. Cromwell EA, Ngondi J, McFarland D, King JD, Emerson PM. Methods for estimating population coverage of mass distribution programmes: a review of practices in relation to trachoma control. Trans R Soc Trop Med Hyg. 2012;106 10:588-95; doi: 10.1016/j.trstmh.2012.07.011. https://www.ncbi.nlm.nih.gov/pubmed/22884927.

927. Checchi F, Cox AP, Chappuis F, Priotto G, Chandramohan D, Haydon DT. Prevalence and under-detection of gambiense human African trypanosomiasis during mass screening sessions in Uganda and Sudan. Parasit Vectors. 2012;5:157; doi: 10.1186/1756-3305-5-157. https://www.ncbi.nlm.nih.gov/pubmed/22871103.

928. Carme B. Rapid assessment procedure for loiasis and mapping lymphatic filariasis: two perfect illustrations of "to be in English or not to be". PLoS Negl Trop Dis. 2012;6 12:e1863; doi: 10.1371/journal.pntd.0001863. https://www.ncbi.nlm.nih.gov/pubmed/23272254.

929. Boatin BA, Basanez MG, Prichard RK, Awadzi K, Barakat RM, Garcia HH, et al. A research agenda for helminth diseases of humans: towards control and elimination. PLoS Negl Trop Dis. 2012;6 4:e1547; doi: 10.1371/journal.pntd.0001547. https://www.ncbi.nlm.nih.gov/pubmed/22545161.

930. Bergquist R, Whittaker M. Control of neglected tropical diseases in Asia Pacific: implications for health information priorities. Infect Dis Poverty. 2012;1 1:3; doi: 10.1186/2049-9957-1-3. https://www.ncbi.nlm.nih.gov/pubmed/23849136.

931. Ault SK, Nicholls RS, Saboya MI. The Pan American Health Organization's role and perspectives on the mapping and modeling of the neglected tropical diseases in Latin America and the Caribbean: an overview. Geospat Health. 2012;6 3:S7-9; doi: 10.4081/gh.2012.116. https://www.ncbi.nlm.nih.gov/pubmed/23032287.

932. Amazigo UV, Leak SG, Zoure HG, Njepuome N, Lusamba-Dikassa PS. Community-driven interventions can revolutionise control of neglected tropical diseases. Trends Parasitol. 2012;28 6:231-8; doi: 10.1016/j.pt.2012.03.002. https://www.ncbi.nlm.nih.gov/pubmed/22503153.

933. Allen T, Parker M. Will increased funding for neglected tropical diseases really make poverty history? Lancet. 2012;379 9821:1097-8; author reply 8-100; doi: 10.1016/S0140-6736(12)60159-7. https://www.ncbi.nlm.nih.gov/pubmed/22293367.

934. Ahmed A, Al-Mekhlafi HM, Azam MN, Ithoi I, Al-Adhroey AH, Abdulsalam AM, et al. Soil-transmitted helminthiasis: a critical but neglected factor influencing school participation of Aboriginal children in rural Malaysia. Parasitology. 2012;139 6:802-8; doi: 10.1017/S003118201100237X. https://www.ncbi.nlm.nih.gov/pubmed/22310239.

935. Integrated preventive chemotherapy for neglected tropical diseases: estimation of the number of interventions required and delivered, 2009-2010. Wkly Epidemiol Rec. 2012;87 2:17-27. https://www.ncbi.nlm.nih.gov/pubmed/22242234.

936. Progress in sanitation needed for neglected tropical diseases. Lancet. 2012;379 9820:978; doi: 10.1016/S0140-6736(12)60412-7. https://www.ncbi.nlm.nih.gov/pubmed/22423871.
