# Supplementary material for: Neglected tropical diseases: an effective global response to local poverty-related disease priorities
Source: Infect Dis Poverty. 2020 Jan 28;9:10. doi: 10.1186/s40249-020-0630-9 (PMC6986060; doi:10.1186/s40249-020-0630-9)
Supplement: Supplementary file 2 — Additional file 2. Electronic Literature Searching Strategy and Results. [file 40249_2020_630_MOESM2_ESM.docx]

***S2***

**Electronic Literature Searching Strategy and Results**

**Searching Strategy**

An electronic literature search was performed from 2012 to 2019 throughout the electronic databases of PubMed/MEDLINE. We determined our search strategy with reference to NTD control at global level and country level, with following five components, e.g. policy with strategy formulation, innovative research, programme management, partner engagement, and evaluation or assessment for the programmes. To maintain the search comprehensiveness, the search was restricted to articles with the following terms in their titles, abstracts and keywords: “NTDs”, or “control”, and “policy” or “strategy”, “innovation “or “research”,“programme” or “management”, “engagement” or “partner”, “evaluation” or “assessment”. We conducted additional search on paper publications and grey literature (conference proceedings, abstracts, masters and doctoral theses) available in office of Uniting to Combat NTDs and Department of Neglected Tropical Diseases, WHO. No restrictions were made on population and languages for the literature search.

**Results**

A total of 948 articles were found through the PubMed/Medline from 2012 to 2019, with the 5 fields are listed in Figure 1, showing the peak years for the publications occurred after 2016, while the highest number was in 2017.

Among 5 fields with the searching terms restricted in title of the publication, the fields with higher number of the publications were in programme management and research innovation, followed by evaluation or assessment, strategy or policy, and partner engagement (Table 1, Figure 2).

Among 5 fields with the searching teams from any fields of the publication, two groups occurred, the toper group was occurred in the research innovation and programme management, which the lower group was occurred in strategy or policy, evaluation or assessment, and partner engagement (Table 2, Figure 3).

Therefore, similar patterns were occurred in both strategy either restricted in the title of the publication or from any fields of the publication, when comparing differences at five fields of the publications.

**Figure 1. Total number of the literature searching through PubMed/Medline.**

**Table 1. Results of the literature searching through PubMed/Medline by five fields restricted in title of the publications.**

| Year | PubMed | Deleting duplication | Title: strategy or policy | Title: innovation or research | Title: management or programme | Title: partner or engagement | Title: economy or assessment |
| --- | --- | --- | --- | --- | --- | --- | --- |
| 2012 | 72 | 67 | 3 | 5 | 9 | 0 | 1 |
| 2013 | 99 | 93 | 3 | 7 | 9 | 1 | 2 |
| 2014 | 123 | 106 | 2 | 4 | 7 | 1 | 1 |
| 2015 | 117 | 105 | 1 | 1 | 7 | 1 | 4 |
| 2016 | 158 | 143 | 2 | 8 | 12 | 2 | 1 |
| 2017 | 172 | 167 | 3 | 7 | 12 | 3 | 3 |
| 2018 | 151 | 142 | 2 | 9 | 13 | 1 | 0 |
| 2019 | 123 | 125 | 4 | 5 | 6 | 1 | 2 |
| Total | 1015 | 948 | 20 | 46 | 75 | 10 | 14 |

**Figure 2. Number of the literature searching through PubMed/Medline by five fields in title of the publication.**

**Table 1. Results of the literature searching through PubMed/Medline by five fields from any fieldse of the publications.**

| Year | PubMed | Deleting duplication | Any fields: strategy or policy | Any fields: innovation or research | Any fields: management or programme | Any fields: partner or engagement | Any fields: evaluation or assessment |
| --- | --- | --- | --- | --- | --- | --- | --- |
| 2012 | 72 | 67 | 18 | 39 | 27 | 7 | 10 |
| 2013 | 99 | 93 | 14 | 67 | 47 | 13 | 21 |
| 2014 | 123 | 106 | 23 | 75 | 51 | 6 | 13 |
| 2015 | 117 | 105 | 20 | 77 | 48 | 10 | 21 |
| 2016 | 158 | 143 | 26 | 100 | 78 | 26 | 21 |
| 2017 | 172 | 167 | 45 | 98 | 79 | 20 | 25 |
| 2018 | 151 | 142 | 32 | 109 | 80 | 11 | 29 |
| 2019 | 123 | 125 | 33 | 69 | 70 | 12 | 22 |
| Total | 1015 | 948 | 211 | 634 | 480 | 105 | 162 |

**Figure 3. Number of the literature searching through PubMed/Medline by five fields in any fields of the publication.**
